# Synthesis and biological study of acridine-based imidazolium salts†

**DOI:** 10.1039/c8ra08138g

**Published:** 2018-11-20

**Authors:** Olla Sharhan, Thorsten Heidelberg, Najiahah Mohd Hashim, Abbas Abdulameer Salman, Hapipah Mohd Ali, Soher Nagi Jayash

**Affiliations:** Chemistry Department, Faculty of Science, University of Malaya (UM) 50603 Kuala Lumpur Malaysia heidelberg@um.edu.my olla_sh@yahoo.com; Chemistry Department, Faculty of Education, Thamar University Yemen; Department of Pharmacy, Faculty of Medicine, University of Malaya Malaysia; Centre for Natural Products and Drug Discovery (CENAR), Faculty of Science, University of Malaya Malaysia; College of Pathological Analysis Technologies, Al-Bayan University Baghdad Iraq; Department of Restorative Dentistry, Faculty of Dentistry, University of Malaya Malaysia; Department of Oral Medicine and Periodontology, Faculty of Dentistry, Ibb University Yemen

## Abstract

A new series of acridine based imidazolium salts was synthesized and evaluated for *in vitro* cytotoxicity against human cancer cell lines by an MTT assay. The synthesis applied a coupling of imidazoles with 9-chloroacridines, which originated from an Ullmann condensation of a 2-chloro-benzoic acid with an aniline. The target compounds were obtained in high yields. The DPPH assay indicated considerable antioxidant activity for target compounds with simple and short alkyl chains on the imidazole, while increasing chain length and the introduction of an additional π-electron system in most cases reduced the activity. All compounds exhibited low biotoxicity against non-cancerous cell lines, whereas a few compounds showed promising anticancer activity. Unlike for the reference drugs Tamoxifen and Paclitaxel, the anticancer activity of acridine imidazolium ions is specific for only selected cancer types. Reasonable fluorescent behaviour of the products provide potential for visualization of the distribution of active drugs in tissue.

## Abstract 2

A series of acridine-based imidazolium salts was synthesized and studied on cytotoxicity against human cancer cell lines.

## Graphical abstract

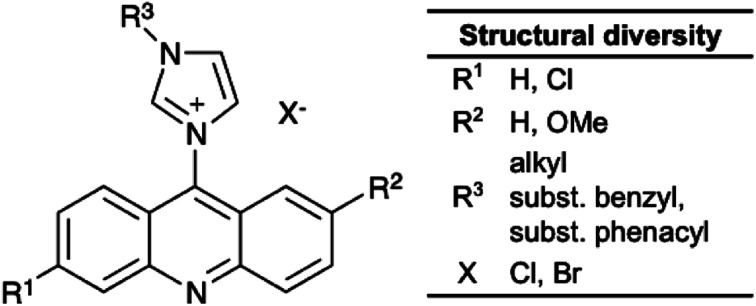

## Introduction

Cancer has become a major cause of mortality, leading to an urgent need for more effective anticancer drugs.^1–6^ The development of new anticancer drugs and efficient treatment strategies for cancer are, hence, of great importance.^7^ 9-Chloroacridine and its derivatives have received interest as a core structure of potential new therapeutics due to a broad biological property spectrum, covering both anticancer and antibacterial activities.^8^ Acridine-based natural products and synthetic derivatives containing imidazole as a second heterocyclic component have found wide use for medicinal applications based on biological and pharmacological activities as antioxidant, antimalarial and antitumor agents.^3^ Acridine and its derivatives are typical intercalative agents for DNA, causing their antitumor activity in chemotherapy. Recently several reviews on acridine derivatives have been published, focusing on their therapeutic potential against cancer and bacteria.^9–11^ They have attracted significant attention as anticancer and antioxidant agents.^2,12^ Natural acridine compounds and synthetic derivatives of 9-chloroacridine and *N*-substituted imidazole have shown a wide range of biological activities, especially antitumor directed. This is matched by reported bioactivities for imidazole and their derivatives,^13^ covering antitumor activity as well.^14^

Basant and co-worker have examined derivatives of acridine with side chains at positions 4 and 5 for inhibitory potential against TAR DNA-binding protein 43 (TDP-43).^15^ Stable N-heterocyclic carbenes (NHCs) are readily accessible from imidazolium compounds.^16^ These secondary derivatives have been reported as effective ligands for certain palladium-catalysed reactions.^17^ The purpose of this study, however, focussed on the synthesis of *N*-substituted acridine-based imidazolium salts and the investigation of their anticancer activities. Introduction of imidazolium ions on acridine can substantially increase the poor water solubility, as recently demonstrates by Raju *et al.*^19^ Several cancer-related patents for 9-amino-substituted acridines^18^ suggest potential of 9-imidazolium-substituted derivatives. This is in line with experiments indicating substantial DNA binding of 9-amino-substituted acridines, although these systems did not contain ionic charges.^1^ In view of the wide medicinal application spectrum of acridine derivatives, we have also studies the antioxidant activity of the compounds using DPPH and FRAP assays, which may be used to guide antimicrobial investigation in future. The fluorescence behaviour of acridine led us to measure UV-Vis absorption and emission spectra as well to evaluate the possibility of potential monitoring of the distribution of acridine-based antitumor reagents in an organism.

## Results and discussion

### Chemistry

**Scheme 1:**
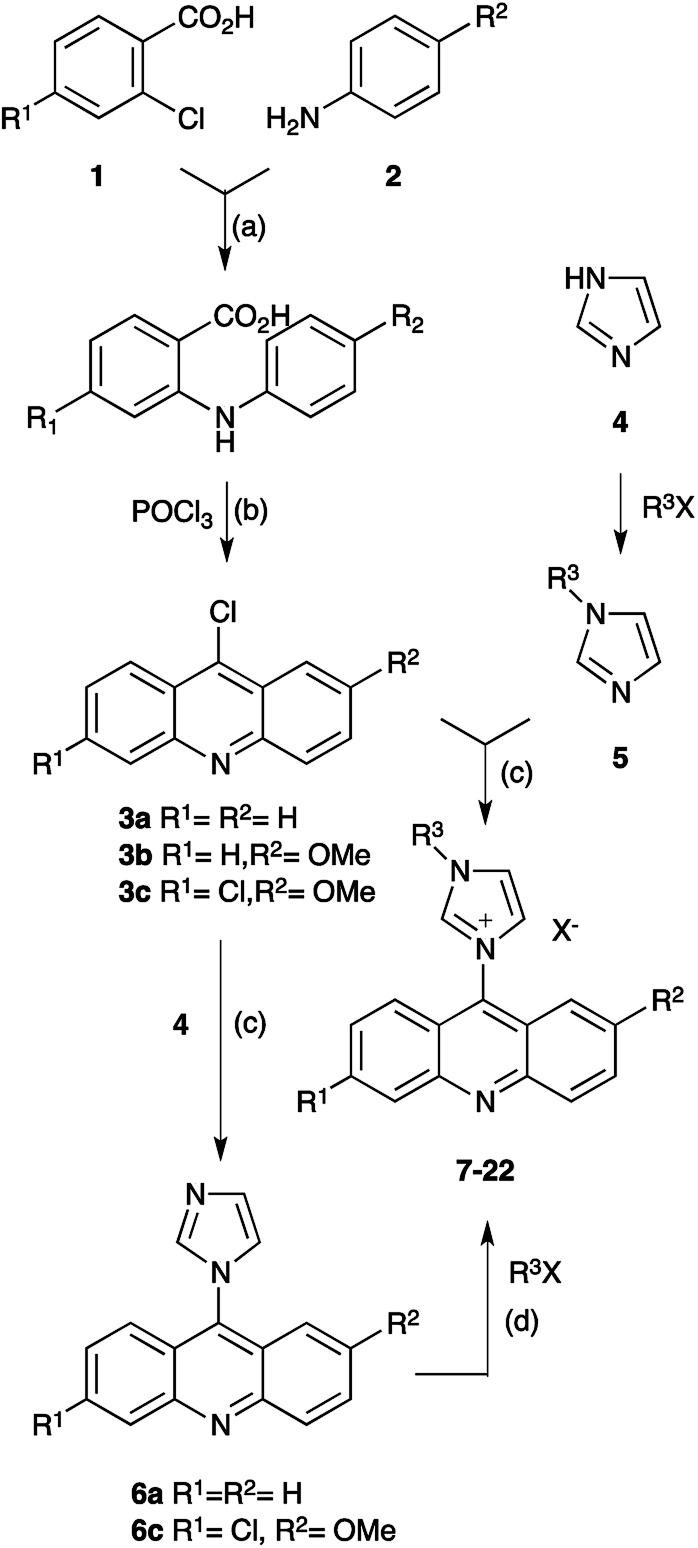
Synthesis of acridine imidazolium salts. (a) KI, Cu, K_2_CO_3_, DMF, reflux, 6–8 h; (b) POCl_3_, 135 °C, 70–85% (a & b); (c) toluene, reflux, 15–18 h, 70–85%: (d) MeCN, reflux, 12–48 h, 85–95%.

The synthesis of the compounds applied two alternative approaches, differing in the alkylation sequence of the imidazole building block. While acridine imidazolium salts with simple saturated hydrocarbon substituents were obtained by condensation of 6-chloroacridines 3 with the respective alkylated imidazole 5,^20–22^ the introduction of more reactive substituents predominately applied derivatives of imidazolyl-acridine 6 and benzyl or phenacyl halides instead. The 6-chloroacridine precursors 3 were obtained by Ullmann condensation of 2-chlorobenzoic acids 1 with anilines 2.^1,2,23,24^ The synthetic scheme is displayed in Scheme 1.

All compounds were obtained in respectable yields, as shown in Table 1. Structure confirmation applied IR and NMR spectroscopy (^1^H & ^13^C), as well as mass spectrometry (ESI in positive mode), while elemental analysis confirmed high purity of the compounds as required for the investigation of biological activity. All compounds exhibited poor solubility in water, but could be well dissolved in DMSO. Therefore tests for biological activity applied DMSO solutions.

**Table 1:** Overview on synthetic compounds

Compd.	Precursor	R^1^	R^2^	R^3^	X	Yield
6a	3a	H	H	—	—	81%
6c	3c	Cl	OCH_3_	—	—	79%
7	3a	H	H	C_6_H_13_	Cl	75%
8	3b	H	OCH_3_	CH_3_	Cl	74%
9	3b	H	OCH_3_	C_4_H_9_	Cl	77%
10	3c	Cl	OCH_3_	CH_3_	Cl	85%
11	3c	Cl	OCH_3_	C_2_H_5_	Cl	81%
12	3c	Cl	OCH_3_	C_4_H_9_	Cl	80%
13	3c	Cl	OCH_3_	C_8_H_17_	Cl	75%
14	6a	H	H	*p*Me-Bn	Cl	85%
15	6a	H	H	*p*Br-Bn	Br	90%
16	6a	H	H	*o*Br-Bn	Br	91%
17	6a	H	H	*p*NO_2_-Bn	Br	91%
18	6a	H	H	BzCH_2_	Br	91%
19	6a	H	H	*p*Br-BzCH_2_	Br	95%
20	3c	Cl	OCH_3_	Bn	Cl	80%
21	6c	Cl	OCH_3_	BzCH_2_	Br	90%
22	6c	Cl	OCH_3_	*p*Br-BzCH_2_	Br	85%

### Antioxidant activity

Antioxidants are substances that may protect cells from the damage caused by unstable molecules known as free radicals. Due to their highly chemical reactivity, free radical damage may lead to cancer.^25,26^ Many biological effects of imidazolium compounds have been related to their antioxidant properties.^27,28^ Therefore the synthesized compounds were evaluated for their antioxidant activity. Antioxidant activity is a complex feature, reflecting different mechanistic pathways.^29^ These cannot be evaluated using a single test; typically two assays with different working principles are, hence, applied.

One of them determines the antioxidant activity by neutralization of coloured DPPH radicals *via* transfer of a hydrogen radical, leading to a discolouration that can be measured photometrically.^30^ The radical-focus of the assay reflects cellular damage processes caused by singlet and triplet oxygen, as well as decomposition of peroxides.^31,32^

The results of the DPPH assay are summarized in Table 2. The data indicate reasonable good radical quenching activity for the non-substituted acridine imidazolium ion 7, comparable with the control compound ascorbic acid. The radical scavenging ability of 7 substantially exceeds that of the non-ionic precursor 6a. Since radicals are species of electron deficiency, it appears surprising that a cationic compound exhibits better radical scavenging potential than the non-ionic compound of a closely related structure. However, the alkylation of imidazole and its related ionization do not affect the conjugated π-electron system, since the utilized free electron pair is perpendicular to the π-system. On the other hand, the imidazolium cation exhibits a more pronounced resonance than the respective non-ionic heterocycle. This is reflected in two resonance structures of almost identical energy, resulting in the distribution of the positive charge over the two ring nitrogen atoms, and probably explains the higher DPPH activity of 7 compared to 6a.

**Table 2:** Antioxidant activity^a^

Compd.	IC_50_ (DPPH) [μg mL^−1^]	FRAP [μg FE mL^−1^]
6a	164 ± 3	<100
6c	144 ± 27	<100
7	49 ± 1	108
8	196 ± 16	<100
9	133 ± 53	<100
10	46 ± 4	<100
11	47 ± 4	<100
12	57 ± 7	<100
13	216 ± 3	<100
14	367 ± 30	211
15	103 ± 42	<100
16	98 ± 6	<100
17	216 ± 41	201
18	367 ± 30	211
19	50 ± 2	<100
20	43 ± 4	<100
21	69 ± 5	<100
22	228 ± 12	170
AA	41 ± 2	337

a Data represent mean of 3 measurements; AA = ascorbic acid, FE = ferric equivalent.

Typically the distribution of radical character and electron deficiency over a compound structure, particularly mediated *via* resonance, stabilizes a radical and, hence potential increases the radical scavenging capacity of the mother-compound of the resulting radical. Therefore increased electron density in a compound is commonly associated with increasing radical stability. However, introduction of a methoxy substituent on the acridine in compounds 8 and 9 significantly lowered the antioxidant activity, whereas the subsequent introduction of a chloride appears to compensate this effect for compounds 10, 11 and 12. The substantially reduced radical scavenging potential of compound 13 cannot be explained by electronic effects, but may reflect solubility issues owing to the significantly increased length of the hydrophobic alkyl chain. The slightly lower activity of 12 compared to 10 and 11 is in line with this explanation.

The introduction of an additional methylene-linked π-electron system at the imidazole in compounds 14–22 potentially increases the conjugation system of radical species, if the H-transfer affects the methylene linker. However, the high DPPH IC_50_ values for compounds 14–18 and 22 suggest either a different preferred site of interaction, *e.g.* the CH in between the two nitrogen atoms of the imidazole ring, or reflect hydrophobic effects as well. Within the compounds that comprised of two methylene-isolated π-electron systems, only 19, 20, and perhaps 21, exhibited highly efficient radical scavenging ability according to the DPPH assay.

The second method for the determination of antioxidant activity applied the FRAP assay. It measures the capacity of a substance to reduce oxidative species, thereby changing the colour, which is determined spectrophotometrically.^29^ Unlike for DPPH, the FRAP assay does not emphasize on radical reactions, but focuses on single electron exchange processes. It measures the antioxidant activity *via* reduction of ferric (Fe^3+^) to ferrous (Fe^2+^) ions, visualized by the coloured ferrous–tripyridyltriazine complex.

Almost all compounds exhibited rather low antioxidant activities according to the FRAP assay compared with the applied reference compound, ascorbic acid. In view of this behaviour and in consideration of the poor correlation of the FRAP and DPPH assay results, the analysis of antioxidant activity was limited to the DPPH assay.

### Biological activity

The potential cytotoxicity of all synthesized substituted acridine-imidazolium salt derivatives was evaluated *in vitro* against a panel of human cancer cell lines. The panel consisted of breast cancer cells (MCF-7), prostate adenocarcinoma cells (PC-3), ovarian cancer cells (CAOV-3), non-tumorigenic ovarian cell line (T1074) and non-tumorigenic breast cell line (MCF-10a). Tamoxifen and Paclitaxel were used as the reference drug for positive control. The results are summarized in Table 3.

**Table 3:** *In vitro* cell toxicity after 24 h exposure^a^

Compd.	IC_50_ [μg mL^−1^]				
	Cancer cell-lines			Non-cancer cell-lines	
	CAOV-3	PC-3	MCF-7	T1074	MCF-10a
6a	12 ± 1	6 ± 1	28 ± 2	40 ± 7	49 ± 9
6c	68 ± 5	77 ± 13	21 ± 1	73 ± 16	66 ± 9
7	80 ± 2	71 ± 7	9 ± 1	90 ± 8	67 ± 3
8	**2.5 ± 0.4**	68 ± 4	41 ± 4	87 ± 12	70 ± 11
9	12 ± 2	62 ± 5	49 ± 3	86 ± 8	50 ± 1
10	77 ± 5	76 ± 18	53 ± 5	66 ± 15	82 ± 5
11	60 ± 1	92 ± 13	23 ± 2	78 ± 10	98 ± 5
12	6 ± 1	75 ± 10	38 ± 5	89 ± 6	>50
13	24 ± 12	28 ± 7	36 ± 7	54 ± 9	>50
14	22 ± 1	9 ± 1	17 ± 2	154 ± 22	97 ± 2
15	4.5 ± 0.7	29 ± 1	24 ± 8	64 ± 9	>50
16	>50	12 ± 6	42 ± 2	46 ± 5	94 ± 26
17	86 ± 10	12 ± 2	56 ± 12	139 ± 21	71 ± 14
18	63 ± 6	93 ± 7	43 ± 7	75 ± 16	>50
19	12 ± 2	87 ± 5	22 ± 5	88 ± 6	50 ± 2
20	27 ± 2	99 ± 7	**5 ± 1**	83 ± 11	83 ± 20
21	5 ± 2	79 ± 9	22 ± 4	75 ± 17	85 ± 10
22	23 ± 5	24 ± 8	20 ± 2	84 ± 5	87 ± 2
Tamoxifen	2 ± 1	2 ± 1	11 ± 1	78 ± 7	85 ± 6
Paclitaxel	4.8 ± 1	5 ± 1	6 ± 1	>50	75 ± 2

a Data represent mean of 3 measurements.

Practically all acridine-based imidazolium salts exhibited unproblematic cell-toxicity against non-cancer cell lines with IC_50_ values above 50 μg mL^−1^. This suggests that the compounds can be safely utilized for cancer therapy, provided that the imidazolium-acridines do not exhibit problematic mutagenic activity, which was not evaluated in the investigation. A potential exception, however, is compound 16, which showed a slightly lower IC_50_ against T1074. Interestingly the activity against cancer cell lines varied significantly for the different cell lines, thereby limiting potential applications to only one specific cancer type. This selectivity, mismatching the more generic profile of Tamoxifen and Paclitaxel, disfavours a pharmaceutical application. The reason for this are separate clinical tests for several drugs with limited application spectrum.

Compound 8 showed high activity against ovarian cancer, surpassing Paclitaxel and practically matching Tamoxifen. However, 8 was practically inactive against prostate and breast cancer. Similar profiles, but with less drastic extremes, were observed for compounds 12, 15 and 21. A comparison of the structures of these active compounds furnished no distinct lead structure, as the active compounds differ in both the acridine core and the imidazole substitution. A structure–activity relation analysis of compounds 7–13, reflecting simple alkyl chain on the imidazole, suggests a medium chain length for the imidazole alkylation and single methoxy-substitution of the acridine as lead structure. The data for compounds 14–22, indicate that the incorporation of aromatic structures at the imidazolium substituent can benefit the activity as well. However, the activity is sensitive towards minor changes in the substitution at the aromatic ring. Overall the activity data for CAOV-3 probably indicate considerable steric constraints for a medium sized substituent at the imidazole, whereas the electronic nature of the alkyl substituent is not of primary relevant.

Varying cytotoxicity has been associated with a variety of rationales, involving charge distribution and water solubility.^33,34^ Particularly effects of chain lengths have been investigated.^35^ However, the obtained data for the CAOV-3 activity of acridine-based imidazolium salts are insufficient to speculate on a rational.

The cytotoxic activity against prostate cancer was rather discouraging. The most active compound was the non-ionic imidazolium-precursor 6a, while only compound 14 exhibited a somehow reasonable, though considerably lower activity compared with the positive controls. The predominately low bioactivities against PC-3 render a structure–activity relation study non-suitable.

Promising breast cancer activity was found for compounds 7 and especially 20, which was more active than any of the positive controls. However, the structural differences of these two compounds do not enable the creation of a lead structure. The same applies for trends, because of the commonly low bioactivity of compounds against MCF-7.

### Fluorescence

**Fig. 1:**
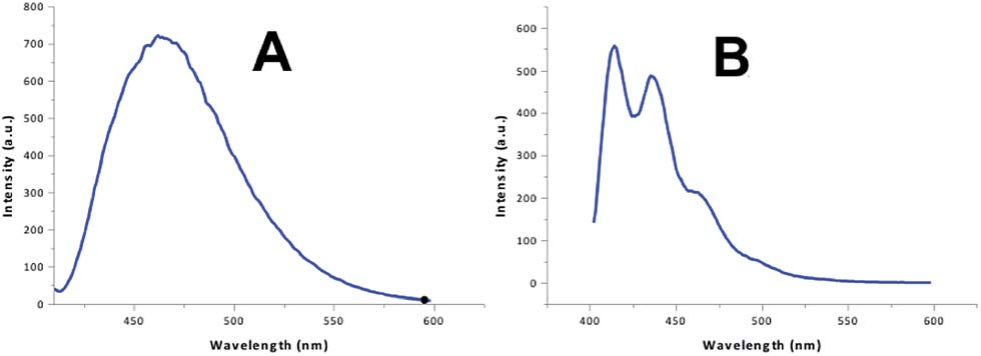
Fluorescence spectra.

Acridine and its derivatives show fluorescence. This feature might be useful to monitor the distribution of acridine based drugs in *in vivo* investigations. Therefore we investigated the fluorescence behaviour of the acridine-based imidazolium salts. The investigation started with the recording of UV-Vis spectra. The wavelength for the maximum at highest wavelength was chosen for the excitation. The fluorescence investigation was conducted at a fixed concentration of 12 μmol L^−1^ in acetonitrile–water to account for the insufficient solubility in water. Two different shapes were found for the fluorescence spectra, which are shown in Fig. 1. The results are summarized in Table 4.

**Table 4:** Fluorescence behaviour^a^

Compd.	*λ*_max abs_ *λ*_exc_ [nm]	Spect_em_	*λ*_max em_ [nm]	Int_em_ [AU]
3c	405	A	451	732
6a	389	B	416, 440	493, 462
6c	400	A	455	774
7	410	A	458	856
8	400	A	465	714
9	401	B	415, 436	553, 488
10	407	A	450	761
11	411	A	471	456
12	410	A	464	640
13	403	A	464	720
14	392	B	420, 435	319, 323
15	387	B	421, 438	571, 582
16	387	A	461	191
17	386	B	416, 448	427, 409
18	384	B	414, 438	608, 540
19	385	B	416, 440	436, 396
20	408	A	465	756
21	409	A	450	798
22	408	A	443	947

a *c* = 12 μmol L^−1^ (acetonitrile/water 1 : 1 v/v).

All compounds only required low energy UV-light for excitation. The blue fluorescence of the sample could easily be visualized using the long wavelength of a standard laboratory UV lamp. Substitution of acridine with an imidazole changes the emission spectrum.^19^ The observed spectra are in line with previously reported 9-amino-substituted acridines,^36–38^ as well as with documented π* → π and π* → n transitions for derivatives of 6,9-dichloro-acridine.^1,39^ Acridine imidazolium cations containing a non-substituted acridine core and substituents involving conjugated systems at the imidazole exhibited double peak fluorescence spectra according to type B in Fig. 1. These peaks have been associated with π* → π transitions on the imidazole and π* → n transitions on both the imidazole and the acridine.^40^ Most of the other compounds, on the other hand, only showed a broad single peak emission, reflecting spectrum type A in Fig. 1. The noticeable red shift of spectra type A may reflect an increased conjugation system due to the acridine substitution. Unlike for *N*-arylated imidazolium cations,^41^ no significant differences were observed between substituents with and without conjugated systems at the imidazole. This can be related to the breaking of conjugation due to the methylene linkage. In terms of fluorescence intensity only a moderate variation was found between the systems. An exception to this, however, is found at compound 16, which exhibited significantly lower fluorescence intensity. A rational for this unusual behaviour could not be found, unless the effect is related to the non-symmetric aromatic at the imidazole (*o*-substitution).

## Experimental

### Materials and methods

Synthesis grade chemicals and solvents of AR grade were obtained from various commercial sources and used without prior treatment. Reactions were monitored by TLC on pre-coated silica 60 aluminum sheets under UV light. Purification of the imidazolium compounds and their precursors applied simple extraction and crystallization. All products were analysed by NMR spectroscopy on 400 MHz spectrometers from Jeol and Bruker. Product purities were confirmed by elemental analysis (CHN). IR spectra were recorded as ATR on a Perkin-Elmer spectrum 400 spectrophotometer. Melting points were determined using an Electrothermal melting point apparatus and are uncorrected. Fluorescence spectra were recorded on an Agilent Cary Eclipse spectrometer at rt in 1 cm quartz cuvettes.

### General procedure A (X = Cl)

A suspension of 3 (1.6 mmol) in toluene (20 mL) was treated with 5 (1.8 mmol) and subsequently heated to reflux overnight. A greenish precipitate developed from the initial solution. After cooling, the solid was collected by filtration, washed with hexane (20 mL) and dried under vacuum to provide the imidazolium salt as greenish solid.

### General procedure B (X = Br)

A solution of 6 (0.25 mmol) and an alkyl or benzyl bromide (0.75 mmol) in acetone or acetonitrile (25 mL) was refluxed for 12–48 h. The imidazolium salt gradually precipitated. The precipitate was filtered after cooling, washed with acetone (2 × 15 mL) and dried to give a yellow solid in a yield of 80–95%.

### 9-(1-Imidazolyl) acridine (6a)

The synthesis followed a modified approach of a previous report.^42^ A solution of 3a (1.0 g, 4.7 mmol) in toluene (60 mL) was treated with imidazole (369 mg, 5.4 mmol) and the reaction mixture was heated to reflux for 2 d, when TLC indicated complete conversion. After cooling, the greenish solid was collected by filtration, washed with hexane (3 × 20 mL), and dried under vacuum to provide 6a (930 mg, 81%) as yellow powder. Mp 210–220 °C.^36^

### 6-Chloro-9-(1-imidazolyl)-2-methoxyacridine (6c)

The synthesis followed a modified approach of a previous report.^42^ A solution of 3c (439 mg, 1.6 mmol) in toluene (20 mL) was treated with imidazole (123 mg, 1.8 mmol) and the reaction mixture was heated to reflux for 2 d, when TLC indicated complete conversion. After cooling, the greenish solid was collected by filtration, washed with hexane (20 mL) and dried under vacuum to provide 6c (389 mg, 79%) as yellow powder. Mp 211–215 °C.^43^

### 1-(Acridin-9-yl)-3-hexylimidazolium chloride (7)

1-Hexyl-imidazole (274 mg, 1.8 mmol) and 3a (337 mg, 1.6 mmol) were reacted according to general method A to give 7 (430 mg, 75%) as off-white solid. Mp 225–230 °C. FTIR: *ν*_max_ (ATR, cm^−1^) 3087, 2954, 2858 (C–H), 1628 (C=N), 1542, 1446, 1410 (C=C), 1267, 1135 (C–O), 766 (C–Cl). ^1^H NMR (400 MHz, DMSO-d_6_): *δ* (ppm) 9.99 (s, 1H, NCHN), 8.40 (d, 2H *J* = 8 Hz), 8.37 (s, 2H), 8.04 (dt ≈ bt, 2H, *J* = 8 Hz), 7.80 (dt ≈ bt, 2H, *J* = 9 Hz), 7.67 (dd ≈ bd, 2H, *J* = 9 Hz), 4.42 (t, 2H, NCH_2_) 2.01 (m_c_, 2H, CH_2_), 1.41–1.31 (m, 6H, CH_2_), 0.89 (t, 3H, CH_3_). ^13^C NMR (100 MHz, DMSO-d_6_): *δ*(ppm) 149.10, 139.21, 132.10, 130.03, 129.55, 125.94, 124.38, 122.55, 122.14, 118.79, 50.35, 31.12, 29.37, 25.83, 22.45, 14.37. Anal. calcd for C_22_H_24_ClN_3_: C 72.22, H 6.61, N 11.84%; found: C 72.19, H 6.59, N 11.80%. HRMS (ESI^+^) *m*/*z* calcd for C_22_H_24_N_3_ [M − Cl]^+^: 330.1970 (100%), 331.2004 (24%); found: 330.1999 (100%), 331.2013 (38%).

### 1-(2-Methoxyacridin-9-yl)-3-methylimidazolium chloride (8)

1-Methylimidazole (441 mg, 5.4 mmol) and 3b^44^ (1.16 g, 4.8 mmol) were reacted according to general method A to give 8 (1.15 g, 74%) as greenish solid. Mp 210–215 °C. FTIR: *ν* (ATR, cm^−1^) 3076, 2961 (C–H), 1631 (C=N), 1561, 1477, 1431 (C=C), 1226, 1136, 1023 (C–O), 758 (C–Cl). ^1^H NMR (400 MHz, DMSO-d_6_): *δ* (ppm) 9.82 (s, 1H, NCHN), 8.31 (d, 1H, *J* = 8 Hz), 8.29–8.26 (m, 3H), 7.95 (dt, 1H, *J*_t_ = 9 Hz, *J*_d_ = 1 Hz), 7.75 (dt, 1H, *J*_t_ = 9 Hz, *J*_d_ = 1 Hz), 7.70 (dd, 1H, *J*_t_ = 9 Hz, *J*_d_ = 3 Hz), 7.62 (d, 1H, *J* = 8 Hz), 6.80 (d, 1H, *J* = 3 Hz), 4.12 (s, 3H, NCH_3_), 3.90 (m, 3H, OCH_3_). ^13^C NMR (100 MHz, DMSO-d_6_): *δ* (ppm) 159.44, 147.10, 146.50, 139.79, 133.66, 132.01, 130.71, 129.97, 129.50, 126.93, 125.67, 125.18, 123.47, 122.57, 122.09, 98.15, 56.60, 37.21. Anal. calcd for C_18_H_16_ClN_3_O: C 66.36, H 4.59, N 12.90%; found: C 66.31, H 4.55, N 12.92%. HRMS (ESI^+^) *m*/*z* calcd for C_18_H_16_N_3_O [M − Cl]^+^: 290.1293 (100%), 291.1327 (20%); found: 290.1302 (100%), 291.1325 (28%).

### 1-(2-Methoxyacridin-9-yl)-3-butylimidazolium chloride (9)

1-Butylimidazole (448 mg, 3.6 mmol) and 3b^44^ (770 mg, 3.2 mmol) were reacted according to general method A to give 9 (890 mg, 77%) as greenish solid. Mp 212–220 °C. FTIR: *ν* (ATR, cm^−1^) 3080, 2965, 2953, 2875 (C–H), 1632 (C=N), 1563, 1478, 1435 (C=C), 1227, 1136, 1022 (C–O), 759 (C–Cl). ^1^H NMR (400 MHz, DMSO-d_6_) *δ* (ppm) 9.92 (s, 1H, NCHN), 8.37 (t, 1H, *J* = 1 Hz), 8.34–8.30 (m, 2H), 8.30 (d, 1H, *J* = 9 Hz), 7.95 (ddd, 1H, *J* = 8/7/1 Hz), 7.78 (ddd, 1H, *J* = 9/8/1 Hz), 7.74 (dd, 1H, *J* = 9/3 Hz), 7.59 (bd, 1H, *J* = 9 Hz), 6.70 (d, 1H, *J* = 3 Hz), 4.40 (t, 2H, NCH_2_), 3.84 (s, 3H, OCH_3_), 1.98 (m, 2H, CH_2_), 1.37 (m, 2H, CH_2_), 0.95 (t, 3H, CH_3_). ^13^C NMR (100 MHz, DMSO-d_6_): *δ* (ppm) 159.47, 147.13, 146.49, 139.23, 132.10, 130.77, 130.05, 129.65, 126.99, 125.62, 124.54, 123.39, 122.45, 121.94, 121.95, 97.79, 56.40, 50.05, 31.39, 19.49, 13.93. Anal. calcd for C_21_H_22_ClN_3_O: C 68.56, H 6.03, N 11.42%; found: C 68.62, H 6.08, N 11.32%. HRMS (ESI^+^) *m*/*z* calcd for C_21_H_22_N_3_O [M − Cl]^+^: 332.1763 (100%), 333.1796 (23%); found: 332.1790 (100%), 333.1803 (37%).

### 1-(6-Chloro-2-methoxyacridin-9-yl)-3-methylimidazolium chloride (10)

1-Methylimidazole (441 mg, 5.4 mmol) and 3c (1.31 g, 4.7 mmol) were reacted according to general procedure A to give 10 (1.44 g, 85%) as greenish solid. Mp 240–245 °C. FTIR: *ν* (ATR, cm^−1^) 3053 (C–H), 1631 (C=N), 1564, 1476, 1423 (C=C), 1216 (C–O), 832 (C–Cl). ^1^H NMR (400 MHz, DMSO-d_6_): *δ* (ppm) 9.77 (bs, 1H, NCHN), 8.35 (dd ≈ bd, 2H, *J* = 5 Hz), 8.27 (bs, 1H), 8.22 (d, 1H, *J* = 9 Hz), 7.75 (dd, 1H, *J* = 9/3 Hz), 7.71–7.67 (m, 2H), 6.80 (d, 1H, *J* = 3 Hz), 4.07 (s, 3H, NCH_3_), 3.86 (s, 3H, OCH_3_). ^13^C NMR (100 MHz, DMSO-d_6_): *δ*(ppm) 159.67, 147.30, 146.93, 139.38, 135.93, 135.43, 134.17, 131.89, 129.98, 128.14, 127.66, 125.47, 124.59, 123.67, 121.24, 98.35, 56.69, 37.24. Anal. calcd for C_18_H_15_Cl_2_N_3_O: C 60.01, H 4.20, N 11.66%; found: C 59.98, H 4.35, N 11.61%. HRMS (ESI^+^) *m*/*z* calcd for C_18_H_15_ClN_3_O [M−Cl]^+^: 324.0904 (100%), 325.0937 (20%), 326.0875 (34%), 327.0908 (7%); found: 324.0898 (100%), 325.0938 (35%), 326.0886 (52%), 327.0906 (10%).

### 1-(6-Chloro-2-methoxyacridin-9-yl)-3-ethylimidazolium chloride (11)

1-Ethylimidazole (519 mg, 5.4 mmol) and 3c (1.3 g, 4.7 mmol) were reacted according to general method A to give 11 (1.41 g, 81%) as greenish solid. Mp 214–216 °C. FTIR: *ν* (ATR, cm^−1^) 3053, 2988 (C–H), 1629 (C=N), 1547, 1432, 1408 (C=C), 1268, 1140 (C–O), 758 (C–Cl). ^1^H NMR (400 MHz, DMSO-d_6_): *δ* (ppm) 9.88 (s, 1H, NCHN), 8.35 (d, 2H, *J* = 2 Hz), 8.30 (bs, 1H), 8.27 (d, 2H, 10 Hz), 7.78–7.66 (m, 3H), 6.73 (d, 1H, 2 Hz), 4.42 (t, 2H, NCH_2_), 3.86 (s, 3H, OCH_3_), 1.60 (t, 3H, CH_3_). ^13^C NMR (100 MHz, DMSO-d_6_): *δ* (ppm) 159.69, 147.32, 146.98, 138.98, 135.46, 134.72, 131.94, 130.07, 128.18, 127.66, 125.40, 124.66, 124.33, 123.63, 121.19, 98.36, 56.62, 45.80, 15.02. Anal. calcd for C_19_H_17_Cl_2_N_3_O: C 60.97, H 4.58, N 11.23%; found: C 60.93, H 4.55, N 11.30%. HRMS (ESI^+^) *m*/*z* calcd for C_19_H_17_ClN_3_O [M − Cl]^+^: 338.1060 (100%), 339.1094 (21%), 340.1031 (34%), 341.1065 (7%); found: 338.1064 (100%), 339.1095 (44%), 340.1045 (56%), 341.1062 (10%).

### 1-(6-Chloro-2-methoxyacridin-9-yl)-3-butylimidazolium chloride (12)

1-Butylimidazole (672 mg, 5.4 mmol) and 3c (1.3 g, 4.7 mmol) were reacted according to general method A to give 12 (1.52 g, 80%) as greenish solid. Mp 220–225 °C. FTIR: *ν* (ATR, cm^−1^) 3079, 2956 (C–H), 1631 (C=N), 1542, 1480, 1426 (C=C), 1238, 1127 (C–O), 830 (C–Cl). ^1^H NMR (400 MHz, DMSO-d_6_): *δ* (ppm) 10.00 (s, 1H, NCHN), 8.42 (bs, 1H), 8.38 (d, 1H, *J* = 2 Hz), 8.33 (d, 1H, *J* = 1 Hz), 8.25 (d, 1H, *J* = 9 Hz), 7.80 (dd, 1H, *J* = 10/2 Hz), 7.75 (dd, 1H, *J* = 10/3 Hz), 7.68 (d, 1H, *J* = 9 Hz), 6.69 (d, 1H, *J* = 3 Hz), 4.46 (t, 2H, NCH_2_), 3.89 (s, 3H, OCH_3_), 2.02 (m, 2H, CH_2_), 1.42 (m, 2H, CH_2_), 0.99 (t, 3H, CH_3_). ^13^C NMR (100 MHz, DMSO-d_6_): *δ* (ppm) 159.47, 147.30, 146.99, 139.23, 135.49, 134.10, 131.99, 130.15, 128.23, 127.71, 125.59, 124.59, 124.49, 123.60, 121.13, 98.00, 56.48, 50.10, 31.36, 19.50, 13.92. Anal. calcd for C_21_H_21_Cl_2_N_3_O: C 62.69, H 5.26, N 10.44%; found: C 62.51, H 5.25, N 10.31%. HRMS (ESI^+^) *m*/*z* calcd for C_21_H_21_ClN_3_O [M − Cl]^+^: 366.1373 (100%), 367.1407 (23%), 368.1344 (35%), 369.1378 (8%); found: 366.1418 (100%), 367.1409 (53%), 368.1361 (66%), 369.1374 (19%).

### 1-(6-Chloro-2-methoxyacridin-9-yl)-3-octylimidazolium chloride (13)

1-Octylimidazole (324 mg, 1.8 mmol) and 3c (439 mg, 1.6 mmol) were reacted according to general method A to give 13 (545 mg, 75%) as yellow solid. Mp 320–330 °C. FTIR: *ν* (ATR, cm^−1^) 3069, 2954, 2924, 2854 (C–H), 1631 (C=N), 1595, 1561, 1476 (C=C), 1281, 1233, 1154, 1030 (C–O), 818, 747 (C–Cl). ^1^H NMR (400 MHz, DMSO-d_6_): *δ* (ppm) 9.86 (s, 1H, NCHN), 8.33 (bs, 1H), 8.31 (s, 1H), 8.28 (d, 1H, *J* = 10 Hz), 7.95 (dt, 1H, *J*_t_ = 7 Hz, *J*_d_ = 2 Hz), 7.77 (dt, 1H, *J*_t_ = 7 Hz, *J*_d_ = 1 Hz), 7.71 (dd, 1H, *J* = 9/3 Hz), 7.56 (d, 1H, *J* = 9 Hz), 6.69 (d, 1H, *J* = 3 Hz), 4.42 (t, 2H, NCH_2_), 3.87 (s, 3H, OCH_3_), 2.00 (m, 2H, CH_2_), 1.36–1.26 (m, 10H, CH_2_), 0.85 (t, 3H, CH_3_). ^13^C NMR (100 MHz, DMSO-d_6_): *δ* (ppm) 159.47, 147.14, 146.50, 139.22, 133.58, 132.11, 130.76, 130.08, 129.61, 126.96, 125.62, 124.53, 123.40, 122.47, 121.91, 97.86, 56.39, 50.32, 31.70, 29.39, 29.07, 28.91, 26.15, 22.62, 14.49. Anal. calcd for C_25_H_29_Cl_2_N_3_O: C 65.50, H 6.38, N 9.17%; found: C 65.52, H 6.35, N 9.19%. HRMS (ESI^+^) *m*/*z* calcd for C_25_H_29_ClN_3_O [M − Cl]^+^: 422.1999 (100%), 423.2033 (28%), 424.1970 (34%), 425.2004 (10%); found: 422.1992 (100%), 423.2543 (%), 424.1968 (46%).

### 1-(Acridin-9-yl)-3-(4-methylbenzyl)-imidazolium chloride (14)

1-(4-Methylbenzyl)-imidazole (310 mg, 1.8 mmol) and 3a (337 mg, 1.6 mmol) were reacted according to general method A to give 14 (520 mg, 85%) as white solid. Mp 205–210 °C. FTIR: *ν* (ATR, cm^−1^) 3020, 2946 (C–H), 1626 (C=N), 1536, 1520, 1439 (C=C), 1259, 1132 (C–C), 770 (C–Cl). ^1^H NMR (400 MHz, DMSO-d_6_): *δ* (ppm) 9.95 (s, 1H, NCHN), 8.34–8.28 (m, 4H), 7.99 (bt, 2H, *J* = 8 Hz), 7.77 (dt, 2H, *J*_t_ = 6 Hz, *J*_d_ = 1 Hz), 7.60 (d, 2H, *J* = 9 Hz), 7.48 (d, 2H, *J* = 8 Hz), 7.27 (d, 2H, *J* = 8 Hz), 5.59 (s, 2H), 2.30 (s, CH_3_). ^13^C NMR (100 MHz, DMSO-d_6_): *δ* (ppm) 149.06, 139.46, 139.04, 136.02, 132.05, 131.65, 130.25, 130.01, 129.57, 129.39, 126.18, 124.39, 122.51, 122.08, 53.26, 21.32. Anal. calcd for C_24_H_20_ClN_3_: C 74.70, H 5.22, N 10.89%; found: C 74.80, H 5.20, N 10.90%. HRMS (ESI^+^) *m*/*z* calcd for C_24_H_20_N_3_ [M − Cl]^+^: 350.1657 (100%), 351.1691 (27%); found: 350.1650 (100%), 351.1682 (74%).

### 1-(Acridin-9-yl)-3-(4-bromobenzyl)-imidazolium bromide (15)

4-Bromobenzyl bromide (748 mg, 3.0 mmol) and 6a (244 mg, 1.0 mmol) were reacted according to general method B to give 15 (445 mg, 90%) as yellow solid. Mp 280–290 °C. FTIR: *ν* (ATR, cm^−1^) 3010, 2950 (C–H), 1633 (C=N), 1534, 1425 (C=C), 1137, 1030 (C–O), 750 (C–Br). ^1^H NMR (400 MHz, DMSO-d_6_): *δ* (ppm) 9.93 (s, 1H, NCHN), 8.39–8.35 (m, 3H), 8.33 (t, 1H, *J* = 2 Hz), 8.03 (dt, 2H, *J*_t_ = 7 Hz, *J*_d_ = 1 Hz), 7.81 (dt, 2H, *J*_t_ = 6 Hz, *J*_d_ = 1 Hz), 7.72 (d, 2H, *J* = 8 Hz), 7.68 (d, 2H, *J* = 8 Hz), 7.60 (d, 2H, *J* = 8 Hz), 5.67 (s, 2H, NCH_2_). ^13^C NMR (100 MHz, DMSO-d_6_): *δ* (ppm) 149.05, 139.59, 135.98, 133.88, 132.62, 132.07, 131.77, 129.98, 129.57, 126.23, 124.47, 123.01, 122.60, 122.06, 52.76. Anal. calcd for C_23_H_17_Br_2_N_3_: C 55.78, H 3.46, N 8.49%; found: C 55.77, H 3.47, N 8.51%. HRMS (ESI^+^) *m*/*z* calcd for C_23_H_17_BrN_3_ [M − Br]^+^: 414.0606 (100%), 415.0639 (26%), 426.0605 (100%), 417.0638 (26%); found: 414.0626 (100%), 415.0641 (35%), 416.0608 (99%), 417.0621 (35%).

### 1-(Acridin-9-yl)-3-(2-bromobenzyl)-imidazolium bromide (16)

2-Bromobenzyl bromide (748 mg, 3.0 mmol) and 6a (244 mg, 1.0 mmol) were reacted according to general method B to give 16 (449 mg, 91%) as yellow solid. Mp 276–278 °C. FTIR: *ν* (ATR, cm^−1^) 3023, 2960 (C–H), 1632 (C=N), 1518, 1423 (C=C), 1269, 1135 (C–O), 750, 806 (C–Br). ^1^H NMR (400 MHz, DMSO-d_6_): *δ* (ppm) 9.93 (s, 1H, NCHN), 8.43 (t, 1H, *J* = 2 Hz), 8.38 (bd, 2H, *J* = 10 Hz), 8.32 (t, 1H, *J* = 2 Hz), 8.04 (dt, *J*_t_ = 6 Hz, *J*_d_ = 1 Hz), 7.84–7.80 (m, 3H), 7.70 (dd ≈ bd, 1H, *J* = 8 Hz), 7.67 (bd, 2H, *J* = 8 Hz), 7.57 (dt, 1H, *J*_t_ = 7 Hz, *J*_d_ = 2 Hz), 7.45 (dt, *J*_t_ = 7 Hz, *J*_d_ = 2 Hz), 5.77 (s, 2H, NCH_2_). ^13^C NMR (100 MHz, DMSO-d_6_): *δ* (ppm) 149.08, 140.18, 135.93, 133.93, 133.30, 132.40, 132.08, 130.05, 129.60, 129.25, 126.35, 124.69, 124.14, 122.45, 122.15, 100.00, 53.97. Anal. calcd for C_23_H_17_Br_2_N_3_: C 55.78, H 3.46, N 8.49%; found: C 55.79, H 3.49, N 8.52%. HRMS (ESI^+^) *m*/*z* calcd for C_23_H_17_BrN_3_ [M − Br]^+^: 414.0606 (100%), 415.0639 (26%), 426.0605 (100%), 417.0638 (26%); found: 414.0621 (100%), 415.0642 (32%), 416.0603 (98%), 417.0622 (32%).

### 1-(Acridin-9-yl)-3-(4-nitrobenzyl)-imidazolium bromide (17)

4-Nitrobenzyl bromide (486 mg, 2.3 mmol) and 6a (183 mg, 0.75 mmol) were reacted according to general method B to give 17 (315 mg, 91%) as yellow solid. Mp 220–230 °C. FTIR: *ν* (ATR, cm^−1^) 3064, 2928 (C–H), 1605 (C=N), 1542, 1513, 1428, 1349 (C=C), 1271, 1140 (C–O), 757 (C–Br). ^1^H NMR (400 MHz, DMSO-d_6_): *δ* (ppm) 9.97 (s, 1H, NCHN), 8.44–8.35 (m, 6H), 8.04 (bt, 2H, *J* = 8 Hz), 7.89 (d, 2H, *J* = 8 Hz), 7.82 (t, 2H, *J* = 8 Hz), 7.72 (d, 2H, *J* = 8 Hz), 5.85 (s, 2H, NCH_2_). ^13^C NMR (100 MHz, DMSO-d_6_): *δ* (ppm) 149.08, 148.33, 141.87, 140.00, 135.95, 132.08, 130.64, 130.01, 129.58, 126.39, 124.70, 122.67, 122.05, 52.59. Anal. calcd for C_23_H_17_BrN_4_O_2_: C 59.88, H 3.71, N 12.15; found: C 59.90, H 3.79, N 12.20%. HRMS (ESI^+^) *m*/*z* calcd for C_23_H_17_N_4_O_2_ [M − Br]^+^: 381.1352 (100%), 382.1385 (25%); found 381.1383 (100%), 382.1393 (40%).

### 1-(Acridin-9-yl)-3-(phenacyl)-imidazolium bromide (18)

2-Bromo-acetophenone (596 mg, 3.0 mmol) and 6a (244 mg, 1.0 mmol) were reacted according to general method B to give 18 (401 mg, 91%) as yellow solid. Mp 190–200 °C. FTIR: *ν* (ATR, cm^−1^) 3064, 2928 (C–H), 1689 (C=O), 1633 (C=N), 1540, 1426, 1424, 1347 (C=C), 1224, 1146 (C–O), 752 (C–Br). ^1^H NMR (DMSO-d_6_): *δ* (ppm) 9.82 (s, 1H, NCHN), 8.44 (dd ≈ bs, 1H), 8.41 (d, 2H, *J* = 8 Hz), 8.25 (t, 1H, *J* = 3 Hz), 8.16 (d, 2H, *J* = 8 Hz), 8.06 (dt, 2H, *J*_t_ = 9 Hz, *J*_d_ = 1 Hz), 7.88 (bd, 2H, *J* = 9 Hz), 7.82 (t, 1H, *J* = 7 Hz), 7.71–7.67 (m, 4H), 6.32 (s, 2H). ^13^C NMR (DMSO-d_6_): *δ* (ppm) 191.73, 149.06, 140.96, 135.98, 135.22, 134.09, 132.04, 130.07, 129.68, 129.64, 128.83, 125.81, 125.52, 122.17, 122.14, 56.65. Anal. calcd for C_24_H_18_BrN_3_O: C 64.88, H 4.08, N 9.46%; found: C 64.86, H 4.05, N 9.51%. HRMS (ESI^+^) *m*/*z* calcd for C_24_H_18_N_3_O [M − Br]^+^: 364.1450 (100%), 365.1483 (27%); found: 364.1433 (100%), 265.1463 (36%).

### 1-(Acridin-9-yl)-3-(4-bromophenacyl)-imidazolium bromide (19)

2,4′-Dibromo-acetophenone (624 mg, 2.2 mmol) and 6a (183 mg, 0.75 mmol) were reacted according to general method B to give 19 (372 mg, 95%) as colourless solid. Mp 220–230 °C. FTIR: *ν* (ATR, cm^−1^) 3081, 2966 (C–H), 1698 (C=N), 1585, 1423, 1393 (C=C), 1269, 1135 (C–O), 987, 749 (C–Br). ^1^H NMR (DMSO-d_6_): *δ* (ppm) 9.81 (t ≈ bs, 1H, NCHN), 8.44 (d, 1H, *J* = 2 Hz), 8.41 (bd, 2H, *J* = 8 Hz), 8.24 (t, 1H, *J* = 2 Hz), 8.09 (d, 2H, *J* = 8 Hz), 8.08–8.06 (m, 2H), 7.92 (d, 2H, *J* = 8 Hz), 7.91–7.86 (m, 2H), 7.68 (d, 2H, *J* = 8 Hz), 6.29 (s, 2H, NCH_2_). ^13^C NMR (DMSO-d_6_): *δ* (ppm) 191.22, 149.12, 140.99, 136.05, 133.23, 132.84, 132.10, 130.84, 130.13, 129.68, 129.40, 125.85, 125.60, 122.22, 56.65. Anal. calcd for C_24_H_17_Br_2_N_3_O: C 55.09, H 3.27, N 8.03; found: C 55.07, H 3.30, N 8.05%. HRMS (ESI^+^) *m*/*z* calcd for C_24_H_17_BrN_3_O [M − Br]^+^: 442.0555 (100%), 443.0589 (27%), 444.0535 (98%), 445.0569 (26%); found 442.1206 (100%), 443.1237 (27%), 444.1186 (31%), 445.1207 (10%).

### 1-(6-Chloro-2-methoxyacridin-9-yl)-3-benzylimidazolium chloride (20)

1-Benzylimidazole (570 mg, 3.6 mmol) and 3c (878 mg, 3.2 mmol) were reacted according to general method A to give 20 (1.1 g, 80%) as greenish solid. Mp 245–250 °C. FTIR: *ν* (ATR, cm^−1^) 2960 (C–H), 1627 (C=N), 1556, 1440, 1409 (C=C), 1141, 1030 (C–O), 763 (C–Br). ^1^H NMR (DMSO-d_6_): *δ* (ppm) 9.92 (s, 1H, NCHN), 8.43 (d, 1H, *J* = 2 Hz), 8.35 (d, 2H, *J* = 1 Hz), 8.27 (d, 1H, *J* = 10 Hz), 7.82 (dd, 1H, *J* = 10/2 Hz), 7.74 (dd, 1H, *J* = 10/3 Hz), 7.68 (d, 1H, *J* = 10 Hz), 7.60 (d, 2H, *J* = 7 Hz), 7.53–7.46 (m, 3H), 6.58 (d, 1H, *J* = 3 Hz), 5.72–5.63 (AB-syst., 2H, NCH_2_), 3.83 (s, 3H, OCH_3_). ^13^C NMR (DMSO-d_6_): *δ*(ppm) 159.70, 147.26, 146.98, 139.61, 135.48, 134.98, 134.09, 131.99, 130.15, 129.70, 129.53, 129.10, 128.22, 127.69, 125.90, 124.75, 124.46, 123.56, 121.02, 97.76, 56.38, 53.41. Anal. calcd for C_24_H_19_Cl_2_N_3_O: C 66.06. H 4.39, N 9.63%; found: C 66.09, H 4.41, N 9.60%. HRMS (ESI^+^) *m*/*z* calcd for C_24_H_19_ClN_3_O [M − Cl]^+^: 400.1217 (100%), 401.1250 (27%), 402.1188 (35%), 403.1221 (8%); found: 400.1214 (100%), 401.1241 (27%), 402.1189 (33%), 403.1215 (8%).

### 1-(6-Chloro-2-methoxyacridin-9-yl)-3-(phenacyl) imidazolium bromide (21)

2-Bromoacetophenone (298 mg, 1.5 mmol) and 6c (154 mg, 0.50 mmol) were reacted according to general method B to give 21 (227 mg, 90%) as yellow solid. Mp 250–260 °C. FTIR: *ν* (ATR, cm^−1^) 3064, 2996 (C–H), 1683 (C=O), 1633 (C=N), 1562, 1477, 1424 (C=C), 1234, 1074 (C–O), 757 (C–Br). ^1^H NMR (DMSO-d_6_): *δ* (ppm) 9.81 (s, 1H, NCHN), 8.43 (dd ≈ bd, 1H, *J* = 2 Hz), 8.39 (t, 1H, *J* = 1 Hz), 8.28 (d, 1, *J* = 10 Hz), 8.25 (t, 1H, *J* = 3 Hz), 8.16 (dd, 2H, *J* = 8/1 Hz), 7.87 (dd, 1H, *J* = 9/2 Hz), 7.82 (tt, 1H, *J* = 8/1 Hz), 7.76 (dd, 1H, *J* = 8/3 Hz), 7.73 (d, 1H, *J* = 10 Hz), 7.69 (t, 2H, *J* = 8 Hz), 6.78 (d, 1H, *J* = 2 Hz), 6.41–6.26 (AB-syst., 2H, NCH_2_), 3.98 (s, 3H, OCH_3_). ^13^C NMR (DMSO-d_6_): *δ* (ppm) 191.97, 159.91, 147.29, 147.0, 141.23, 135.51, 135.32, 134.09, 132.02, 130.23, 129.75, 128.90, 128.29, 127.78, 126.03, 125.21, 124.21, 123.86, 121.10, 100.01, 97.56, 56.76, 56.54. Anal. calcd for C_25_H_19_BrClN_3_O_2_: C 59.02, H 3.76, N 8.26%; found: C 59.21, H 3.78, N 8.31%. HRMS (ESI^+^) *m*/*z* calcd for C_25_H_19_ClN_3_O_2_ [M − Br]^+^: 428.1166 (100%), 429.1199 (28%), 430.1137 (34%), 431.1170 (10%); found: 428.1194 (100%), 429.1207 (39%), 430.1157 (45%), 431.1178 (13%).

### 1-(6-Chloro-2-methoxyacridin-9-yl)-3-(4-bromophenacyl)-imidazolium bromide (22)

2,4′-Dibromoacetophenone (624 mg, 2.2 mmol) and 6c (231 mg, 0.83 mmol) were reacted according to general method B to give 22 (414 mg, 85%) as yellow solid. Mp 260–270 °C. FTIR: *ν* (ATR, cm^−1^) 3072, 2983 (C–H), 1693 (C=N), 1634, 1584, 1477 (C=C), 1425, 1235, 1067 (C–O), 812 (C–Br). ^1^H NMR (DMSO-d_6_): *δ* (ppm) 9.78 (s, 1H, NCHN), 8.44 (d, 1H, *J* = 2 Hz), 8.39 (t, 1H, *J* = 1 Hz), 8.29 (d, 1H, *J* = 10 Hz), 8.23 (t, 1H, *J* = 2 Hz), 8.08 (d, 2H, *J* = 8 Hz), 7.92 (d, 2H, *J* = 8 Hz), 7.86 (dd, 1H, *J* = 10/2 Hz), 7.76 (dd, 1H, *J* = 8/2 Hz), 7.73 (d, 1H, *J* = 8 Hz), 6.76 (d, 1H, *J* = 3 Hz), 6.36–6.22 (AB-syst., 2H, NCH_2_), 3.97 (s, 3H, OCH_3_). ^13^C NMR (DMSO-d_6_): *δ* (ppm) 191.38, 159.89, 147.30, 147.01, 141.20, 135.50, 134.16, 133.18, 132.84, 132.03, 130.84, 130.84, 129.45, 128.29, 127.76, 126.01, 125.23, 124.22, 123.87, 121.11, 97.56, 56.70, 56.53. Anal. calcd for C_25_H_18_Br_2_ClN_3_O_2_: C 51.09, H 3.09, N 7.15%; found: C 51.11, H 3.05, N 7.31%. HRMS (ESI^+^) *m*/*z* calcd for C_25_H_18_BrClN_3_O_2_ [M − Br]^+^: 506.0271 (75%), 507.0304 (21%), 508.0270 (100%), 509.0294 (27%), 510.0232 (27%), 511.0265 (8%); found: 506.0282 (85%), 507.0306 (28%), 508.0267 (100%), 509.0285 (33%), 510.0238 (31%), 511.0261 (7%).

### Biological activity

#### Cell culture

MCF-7, MCF-10, PC-3, CAOV-3 and T1074 cell line were originally obtained from the American Type Culture Collection (ATCC; Manassas, VA) as shown in Table 5. The cells were cultured in RPMI-1640 medium. The medium was supplemented with 10% fetal bovine serum (FBS) and 1% antibiotics (penicillin–streptomycin) and incubated at 37 °C in humidified CO_2_ incubator with 5% CO_2_. The medium was changed twice a week until confluent cell monolayer was formed and observed under an inverted microscope.^45^

**Table 5:** Normal and cancerous cell lines

Cell lines	Classification	Source
MCF-7	Breast cancer cells	American Type Culture Collection (ATCC)
MCF-10	Normal breast cancer cells	
CAOV-3	Ovarian cancer cells	
T1074	Normal ovarian cancer cells	
PC-3	Prostate adenocarcinoma cells	

#### Cellular viability assay (MTT assay)

The inhibitory effect of compounds was determined by an MTT assay, in which 5 × 10^3^ cells per well were seeded in 96-well plates and kept for 24 hours (h) at 37 °C with 5% CO_2_ saturation. After this incubation, a serial dilution of different concentrations of compounds were prepared and transferred to the 96 well plates containing the seeded cells and incubated for another 24 h at 37 °C and 5% CO_2_. Subsequently, 20 μL of MTT (3-[4,5-dimethylthiazol-2-yl]-2,5-diphenyltetrazolium bromide, 5 mg mL^−1^) was added to the treated cells in a dark place, covered with foil and incubated for 4 h. All media was discharged and a total of 100 μL volume of DMSO was poured into each well until the purple formazan crystals dissolved. The plate was measured using a microplate reader at absorbance 570 nm. The experiment was conducted in triplicate to evaluate the IC_50_. The percentage of cytotoxicity was determined using the following formula:
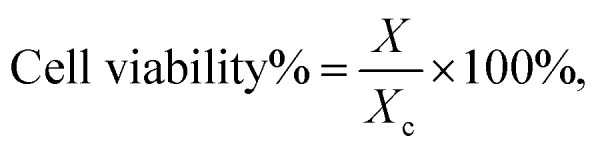
where *X* is the absorbance of treated cells, and *X*_c_ is the absorbance of the control group (untreated cells). Based on the reference, cytotoxicity responses were qualitatively rated as severe, moderate, slight and non-cytotoxic when the cytotoxicity percentage was < 30%, 30–59%, 60–90% and > 90%, respectively.^46^

### Antioxidant activity

#### DPPH assay

The assay was performed in a 96-well microtiter plate according to a modified method by Orhan *et al.*^47^ and Brem *et al.*^48^ A solution of 30 μL DPPH (1.5 mg mL^−1^) in 70 μL DMSO was treated with different concentrations of the compounds, ranging from 15.6 to 1000 μg mL^−1^. Ascorbic acid was used as positive control, while the last row of the plate only contained blank samples of DPPH in DMSO as reference. The plate was incubated for 30 min in the dark and the decrease in absorbance at 517 nm was determined using Tecan micro plate reader (Infinite M200PRO). The radical scavenging activity was calculated using the following formula:
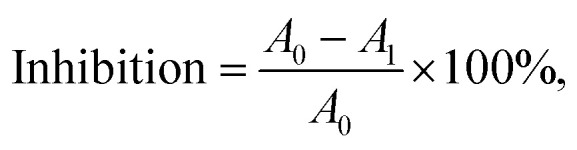
where *A*_0_ is the absorbance of the DPPH radical in the blank sample and *A*_1_ the corresponding absorbance in presence of the sample. The correlation between each concentration and its scavenging was plotted on a graph, and the IC_50_ was determined from the graph as the concentration required to reduce the DPPH absorption by 50%.^49^

#### FRAP assay

The determination of the total antioxidant activity followed a modified method of Benzie and Strain (1999).^50^ The ferric reducing antioxidant power (FRAP) was determined by using freshly prepared reagent based on mixing 300 mM acetate buffer, 10 mM TPTZ (2,4,6-tripyridyl-*s*-triazine) and a solution combining 20 mM iron(iii) chloride (FeCl_3_ × 6H_2_O) and 40 mM HCl in a ratio of 10 : 1 : 1. For measurement, 10 μL of samples (1 mg mL^−1^) and 300 μL FRAP reagent were mixed in multiwell plates and readings of the coloured product (ferrous tripyridyltriazine complex) were taken at 593 nm. Ascorbic acid and ferrous sulphate were used as control and standard, respectively. The FRAP activity was calculated as ferrous equivalents (FE) at a single concentration of 1 mg mL^−1^ and the FE was calculated from the standard curve of FeSO_4_.^51^ A linear calibration curve covering the range of 100 and 1000 mM FeSO_4_ was used to convert the absorption readings to FE. The results were expressed as mM Fe(ii)/g dry weight of the compound.

### Statistical analysis

All analyses were performed in triplicates. Results were expressed as a means ± standard deviation (SD).

## Conclusions

Acridine-based imidazolium cations are easily accessible compounds, which can exhibit promising therapeutic potential for cancer therapy. High reaction yields and simple purification by crystallization provide economic viability, while the Ullmann-based synthesis of the acridine core provides structural diversity for the heterocyclic core. None of the investigated compounds exhibited significant cell toxicity against human non-cancer cell lines. This suggests a safe application of the potential drug. Unlike the cancer drugs Tamoxifen and Paclitaxel, the therapeutic potential of acridine-based imidazolium salts is highly specific for certain cancer types, requesting for different drugs for the therapy of breast, prostate and ovarian cancers. Nonetheless, for all these cancers promising candidates, matching the efficacy of current market drugs Tamoxifen and Paclitaxel, have been identified. The fluorescence of the acridine core might enable studies on the distribution of drug molecules in living tissue, as almost all compounds show visible fluorescence upon low energy UV excitation. However, additional studies are required to evaluate the fluorescence in view of biological background luminescence.

## Conflicts of interest

There are no conflicts to declare.

## Supplementary Material

RA-008-C8RA08138G-s001
