# Supplementary material for: Synthesis and biological study of acridine-based imidazolium salts
Source: RSC Adv. 2018 Nov 20;8(68):38995–9004. doi: 10.1039/c8ra08138g (PMC9090615; doi:10.1039/c8ra08138g)
Supplement: RA-008-C8RA08138G-s001 [file RA-008-C8RA08138G-s001.pdf]

Electronic Supplementary Material (ESI) for RSC Advances.
This journal is © The Royal Society of Chemistry 2018

Electronic Supplementary Information (ESI)

Synthesis and biological studies of acridine derivatives-based imidazolium salt.

Olla Sharhan[a,b*], Thorsten Heidelberg[a*], Najiahah Mohd Hashim[c,d], Abbas Abdulameer Salman[a,e], Hapipah Mohd Ali[a], Soher Nagi Jayash[f,g]

[a] Chemistry Department, Faculty of Science, University of Malaya, Kuala Lumpur, 50603, Malaysia.

[b] Chemistry Department, Faculty of Science, Thamar University, Yemen.

[c] Department of Pharmacy, Faculty of Medicine, University of Malaya, Kuala Lumpur, 50603, Malaysia.

[d] Centre for Natural Products and Drug Discovery (CENAR), Department of Chemistry, Faculty of Science, University of Malaya, Kuala Lumpur, 50603, Malaysia

[e] College of Pathological Analysis Technologies, Al-Bayan University, Baghdad, Iraq.

[f] Department of Restorative Dentistry, Faculty of Dentistry, University of Malaya, Kuala Lumpur, 50603, Malaysia.

[g] Department of Oral Medicine and Periodontology, Faculty of Dentistry, Ibb University, Yemen.

*Corresponding author: Thorsten Heidelberg. E-mail: heidelberg@um.edu.my

Olla Sharhan. E-mail: olla_sh@yahoo.com

TABLE OF CONTENTS

1. General Experimental...S2
2. Experimental Procedures and Analytical Data...S2-S7
3. ^{1}H-NMR and ^{13}C-NMR Spectral of all Compounds...S8-S50
4. UV-Vis data...S 50

1. General Experimental

Melting points were measured on an Electrothermal melting point apparatus. Proton and Carbon-13 nuclear magnetic resonance spectra were recorded on a Bruker Avance and a Joel 400 spectrometer at 400 MHz. Chemical shifts are reported as δ values in parts per million (ppm) relative to tetramethylsilane (TMS). Elemental analyses (%CHN) were conducted on a Perkin Elmer 2400 series. Thin layer chromatography was carried out on pre-coated silica gel plates (silica 60 F_{254}, Merck). Starting materials and reagents used in reactions were obtained from Merck and Sigma Aldrich, and used without purification, unless otherwise indicated.

2. Experimental Procedures and Analytical Data

2.1 2-Phenylamino benzoic acid

A mixture of 2-chlorobenzoic acid or a derivative (38 mmol), aniline or a derivative (49 mmol), potassium iodide (0.25 mmol, 41 mg) and copper powder (2.0 mmol, 120 mg) in 40 mL DMF was slowly treated with dry potassium carbonate (43 mmol, 6.0 g), and the reaction was refluxed for 6 to 8 hours. After cooling to room temperature the reaction mixture was poured into 30 mL of water and decolorized with charcoal. The mixture was filtered through celite and the filtrate was acidified with concentrated hydrochloric acid (HCl, 1 M) (pH was adjusted to 1-2). The precipitating product was filtered and subsequently dissolved in 100 mL of 5% aqueous sodium carbonate solution (stirring 20 min). The solution was filtered through celite and the purified product was obtained by precipitation with acid as described above. **2-Phenylamino benzoic acid** and its derivatives were obtained as off-white solids.

2.2 9-Chloroacridine and derivatives

2-Phenylamino benzoic acid or a derivative (19 mmol) and phosphorus oxy chloride ($POCl_3$) (40 ml) were added into a dried round bottom flask and heated slowly on a water bath to 85-90 ºC for 15 min. The reaction was monitored carefully; if it turned too violent, the reaction was temporary placed in a cold-water bath until the boiling subsided. After the initial exothermic reaction had seized, the flask was immersed into an oil bath. The temperature was raised to 135-140 ºC and the reaction was kept under reflux for 3 h. Excess of phosphorus oxy chloride was subsequently removed by

distillation at 140-150 ºC under vacuum. The cooled reaction mixture was poured into a stirred mixture of 25 ml concentrated aqueous ammonia and 13 g of crushed ice. The 9-chloroacridines precipitated within 30 min. They were filtered, washed with saturated sodium carbonate and with water, dried and recrystallized from ethanol to form needle-shaped crystals (**3a-3c**).

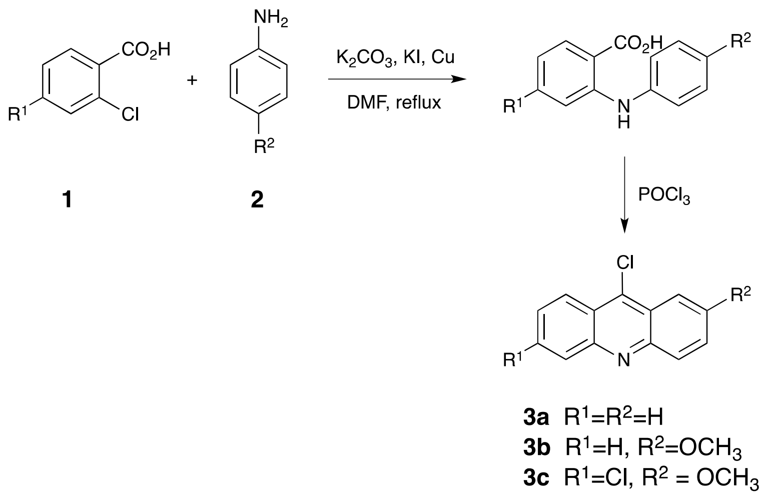

Synthesis of 9-chloroacridines

2.2 Synthesis of N-substituted imidazoles

To a solution of imidazole (16 mmol) in THF (60 mL) was added alkyl bromide (14 mmol) and the reaction was stirred at room temperature for about 48 h. NaOH pellets (18 mmol) were added to the mixture and stirring was continued for another 12 h. The reaction mixture was filtered and extracted twice with CH_2Cl_2 against water. The organic phase was dried over anhydrous $MgSO_4$ and the solvent was removed on a rotary evaporator. The pure N-substituted imidazoles were obtained as yellow liquids.

9-Chloroacridine (3a)

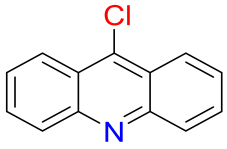

Compounds **3a** was prepared according to previous reports [1-4]. A mixture of 2-phenylamino-benzoic acid (4.0 g, 19 mmol) and $POCl_3$ (40 mL) was heated slowly in an oil bath to 85-90 °C for 15 min. The temperature was subsequently increased to 135-140 °C, and the reaction was kept at reflux for 3 hr. After the completion of the reaction, excess phosphorous oxychloride was removed by vacuum distillation. The reaction mixture was cooled to room temperature and poured into a stirred mixture of 25 ml concentrated ammonia and crushed ice. The crude 9-chloroacridine precipitated within 30 min. The precipitate was filtered by suction, washed three times with saturated $NaHCO_3$ and subsequently with water. The precipitate was dried and recrystallized from ethanol to furnished **3a** as pale brown crystals. Yield: 90 %; mp 115-120 ºC. FTIR (ν cm^{-1}): 3057, 2995 (C-H), 1554, 1467 (C=C), 757 (C-Cl). ^{1}H NMR ($CDCl_3$) δ 8.4 (d, 2H, *J*=8 Hz), 8.26 (d, 2H, *J*=8 Hz), 7.85–7.81 (m, 2H), 7.68-7.64 (m, 2H). ^{13}C NMR ($CDCl_3$) δ 148.9, 141.5, 130.8, 129.8, 127.1, 124.8, 124.4.

9-Chloro-2-methoxyacridine (3b)

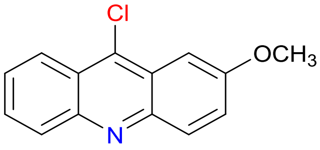

Compound **3b** was prepared according to previous reports [1-4] following the same method that was applied for compound **3a**. A mixture of 2-(4-methoxyphenylamino)-benzoic acid (4.57 g, 19 mmol) and $POCl_3$ (40 mL) was heated slowly in oil bath to 85-90 °C for 15 min. The temperature was subsequently increased to 135-140 °C, and the reaction was kept at reflux for 3 hr. After the completion of the, excess phosphorous oxychloride was removed by vacuum distillation. The reaction mixture was cooled to room temperature and poured into a stirred mixture of 25 ml concentrated ammonia and crushed ice. Crude **3b** precipitated within 30 min. The precipitate was filtered by suction, washed three times with saturated $NaHCO_3$ and subsequently with water. The precipitate was dried over phosphorus pentoxide and recrystallized from ethanol to furnished **3b** as brown crystals. Yield: 95%; mp 160-165 ºC. ^{1}H NMR ($CDCl_3$) δ 8.40(d, 1H, *J*=8 Hz). 8.32 (d, 1H, *J*=12 Hz), 8.14 (d, 1H, *J*=8 Hz), 7.79-7.75

(m, 1H), 7.67-7.63 (m, 1H), 7.54–7.49 (m, 2H), 4.05 (s, 3H, OCH_3). ^{13}CNMR ($CDCl_3$) δ 158.2, 147.3, 146.2, 138.2, 131.5, 129.8, 129.4, 127.1, 126.0, 125.3, 124.5, 124.2, 99.9, 55.7.

6,9-Dichloro-2-methoxyacridine (3c)

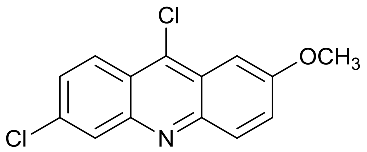

Compound **3c** was prepared according to previous reports [1-4] following same method that was applied for compound **3a**. A mixture of 4-chloro-2-(4-methoxyphenylamino)-benzoic acid (5.21 g, 19 mmol) and $POCl_3$ (40 mL) was heated slowly in oil bath to 85-90 °C for 15 min. The temperature was subsequently increased to 135-140 °C, and the reaction was kept at reflux for 3 hr. After the completion of the reaction, excess phosphorous oxychloride was removed by vacuum distillation. The reaction mixture was cooled to room temperature and poured into a stirred mixture of 25 ml concentrated ammonia and crushed ice. Crude **3c** precipitated within 30 min. The precipitate was filtered by suction, washed three times with saturated $NaHCO_3$ and subsequently with water. It was dried over phosphorus pentoxide and recrystallized from ethanol to furnished **3c** as a yellow powder. Yield: 92%; mp 160-165 ºC [5]. ^{1}H NMR ($CDCl_3$) δ 8.23 (d, 1H, *J*=9 Hz) 8.13 (s, 1H), 8.03 (d, 1H, *J*=8 Hz), 7.47 (t, 2H, *J*=8 Hz), 7.39 (s, 1H), 3.99 (s, 3H, OCH_3). ^{13}C NMR ($CDCl_3$) δ 158.5, 146.6, 146.2, 138.9, 135.9, 131.1, 128.4, 127.8, 126.9, 125.7, 125.3, 122.8, 99.9, 55.9.

9-(1-Imidazolyl) acridine (6a)

FTIR: ν (ATR, cm^{-1}) 3090 (C–H), 1629 (C=N), 1555, 1478, 1421 (C=C). ^{1}H NMR ($CDCl_3$): δ 8.35 (t, 1H, *J*=1 Hz), 8.32 (t, 1H, *J*=1 Hz), 7.88-7.84 (m, 3H), 7.60-7.59 (m, 4H), 7.49 (t, 1H, *J*=1 Hz), 7.36 (t, 1H, *J*=1 Hz). ^{13}C NMR ($CDCl_3$): 149.2, 139.0, 138.3, 130.7, 130.1, 129.8, 127.6, 122.9, 122.5, 122.3. Anal. calcd. for $C_{16}H_{11}N_3$: C 78.35, H 4.52, N 17.13 %, found: C 78.31, H 4.54, N 17.15%.

6-Chloro-9-(1-imidazolyl)-2-methoxyacridine (6c)

FTIR: ν (ATR, cm^{-1}) 3095 (C–H), 1633 (C=N), 1560, 1475, 1421 (C=C). ^{1}H NMR ($CDCl_3$): δ 8.33 (d, 1H, *J*= 1 Hz), 8.22 (d, 1H, *J*=8 Hz), 8.04 (s, 1H), 7.59-7.51(m, 3H), 7.43 (s, 1H), 7.41 (s, 1H), 6.57 (d, 1H, *J*=4 Hz), 3.84 (s, 3H, OCH_3). ^{13}C NMR ($CDCl_3$): δ 159.2, 147.3, 147.2, 136.2, 135.8, 131.5, 130.1, 129.3, 128.3, 127.1, 124.3, 123.4, 121.7, 97.2, 55.8. Anal. calcd. for $C_{17}H_{12}ClN_3O$: C 65.92, H 3.90, N 13.57 %; found: C 65.90, H 3.91, N 13.59 %.

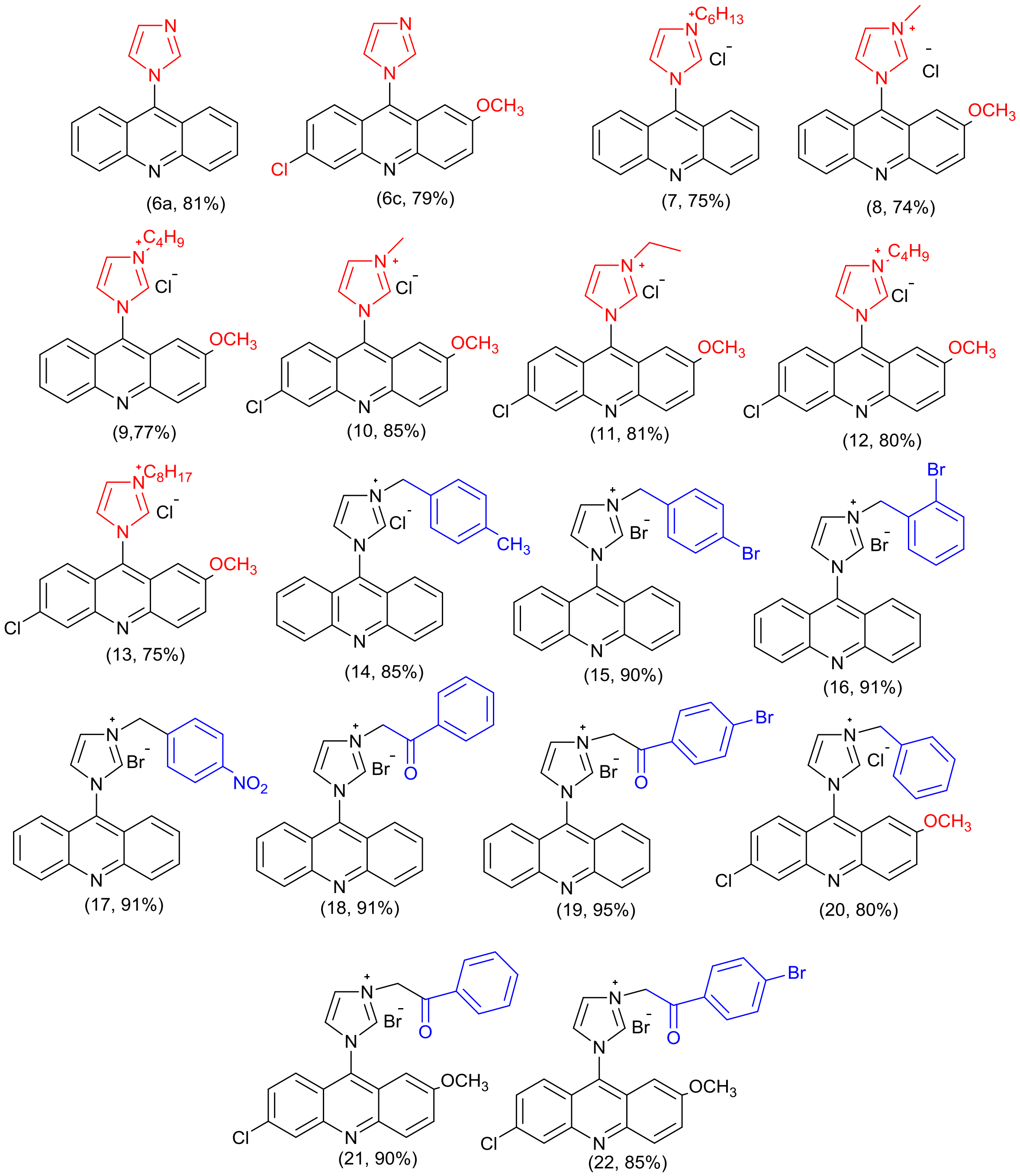

Overview on N-substituted imidazolium-acridine salts

References

1. X. Lang, L. Li, Y. Chen, Q. Sun, Q. Wu, F. Liu, C. Tan, H. Liu, C. Gao, Y. Jiang, Novel synthetic acridine derivatives as potent DNA-binding and apoptosis-inducing antitumor agents. *Bioorganic & Medicinal Chemistry*, 2013. **21**(14), 4170-4177.

2. R. Csuk, A. Barthel, and C. Raschke, Convenient access to substituted acridines by a Buchwald–Hartwig amination. *Tetrahedron*, 2004. **60**(27), 5737-5750.

3. V. Nadaraj, S.T. Selvi, and S. Mohan, Microwave-induced synthesis and anti-microbial activities of 7,10,11,12-tetrahydrobenzo[c]acridin-8(9H)-one derivatives. *European Journal of Medicinal Chemistry*, 2009. **44**(3), 976-980.

4. B. Li, C.-M. Gao, Q.-S. Sun, L.-L. Li, C.-Y. Tan, H.-X. Liu, Y.-Y. Jiang, Novel synthetic acridine-based derivatives as topoisomerase I inhibitors. *Chinese Chemical Letters*, 2014. **25**(7), 1021-1024.

5. M. Staderini, M.L. Bolognesi, and J.C. Menéndez, Lewis acid-catalyzed generation of C-C and C-N bonds on π-deficient heterocyclic substrates. *Advanced Synthesis & Catalysis*, 2015, **357**(1), 185-195.

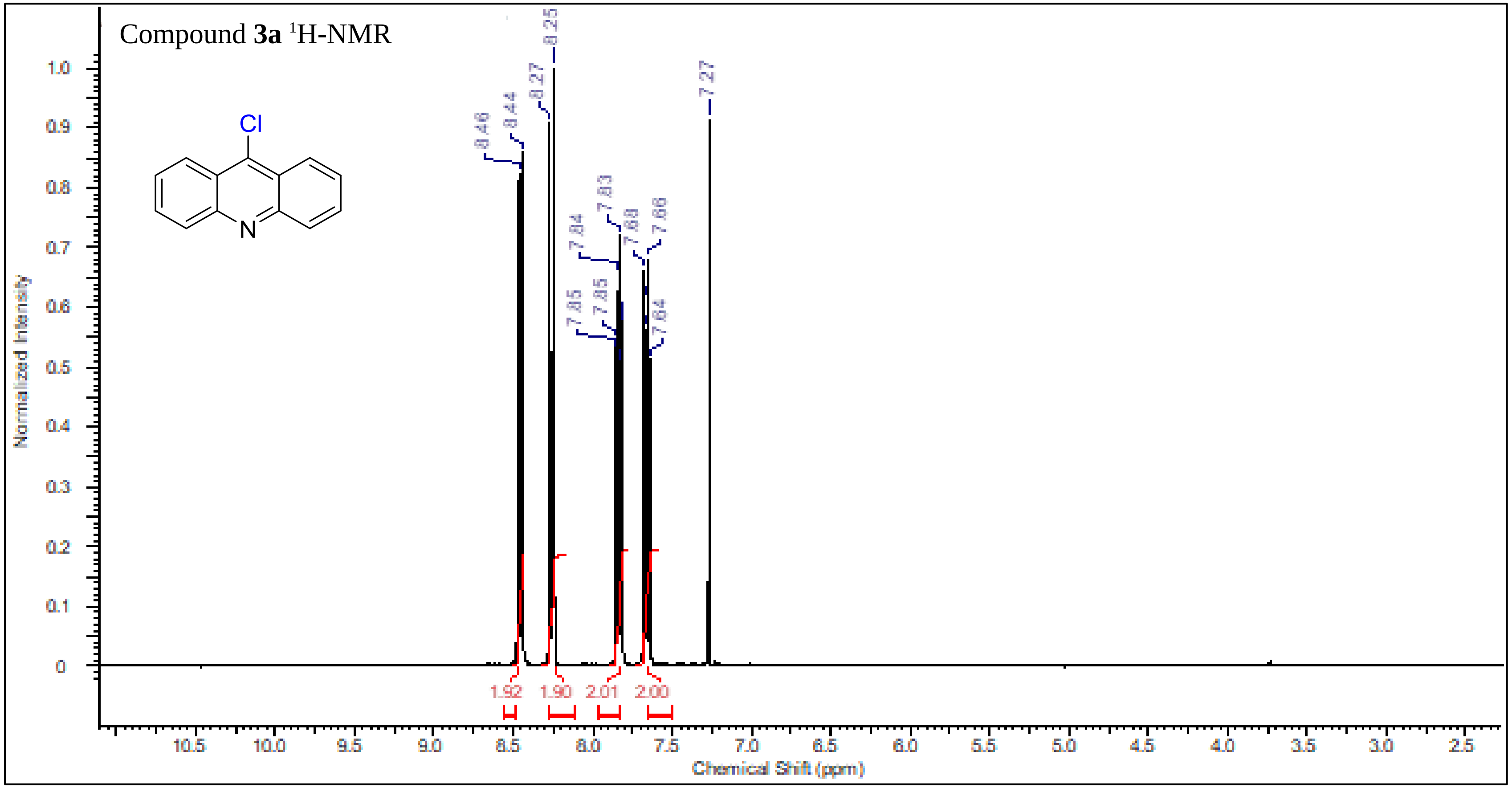
Compound 3a ¹H-NMR
Cl
N
8.46
8.44
8.27
8.25
7.85
7.85
7.84
7.83
7.68
7.66
7.64
7.27
1.92
1.90
2.01
2.00
Normalized Intensity
Chemical Shift (ppm)

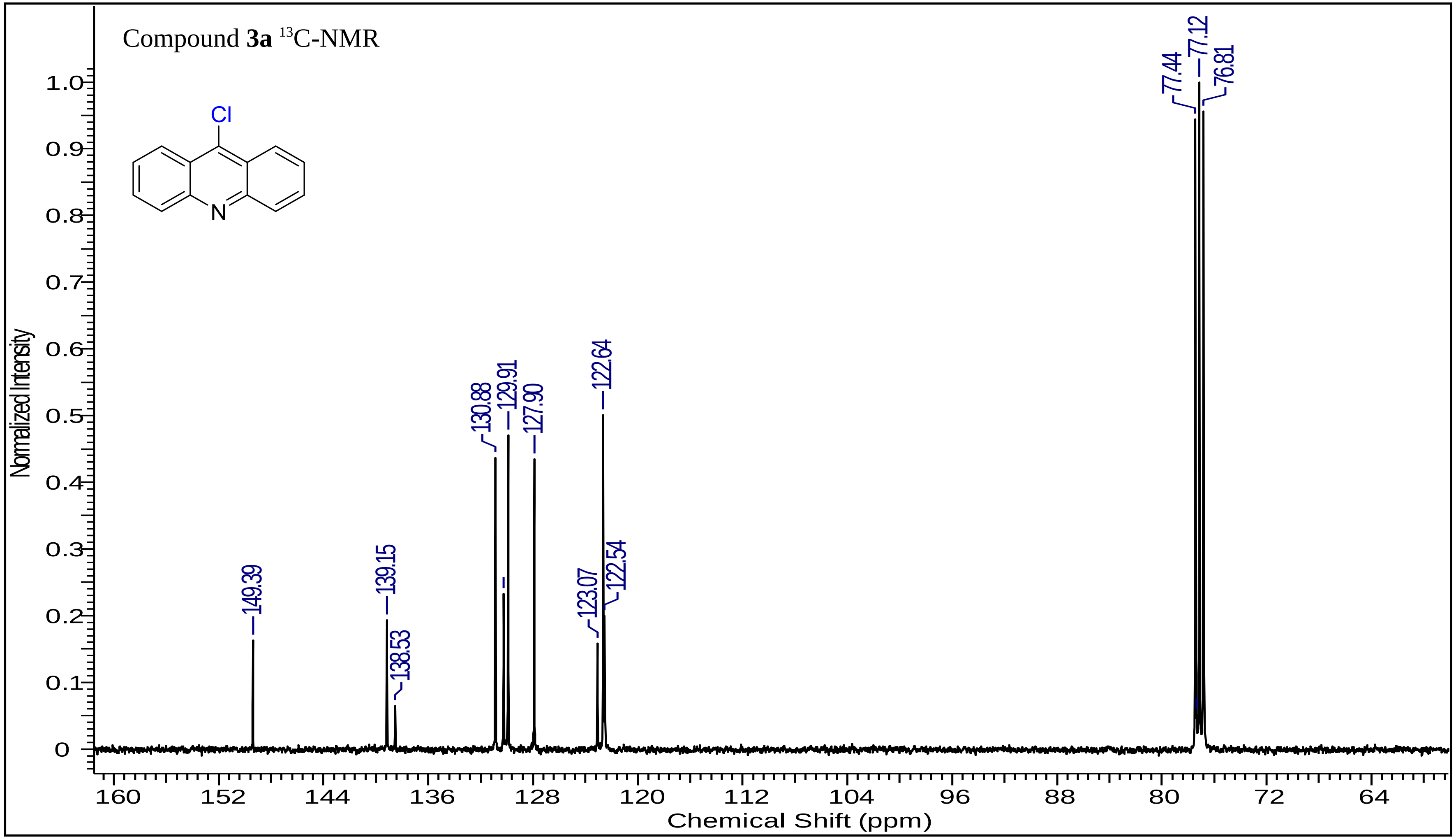
Compound 3a 13C-NMR
Cl
N
149.39
139.15
138.53
130.88
129.91
127.90
123.07
122.64
122.54
77.44
77.12
76.81
Normalized Intensity
1.0
0.9
0.8
0.7
0.6
0.5
0.4
0.3
0.2
0.1
0
160
152
144
136
128
120
112
104
96
88
80
72
64
Chemical Shift (ppm)

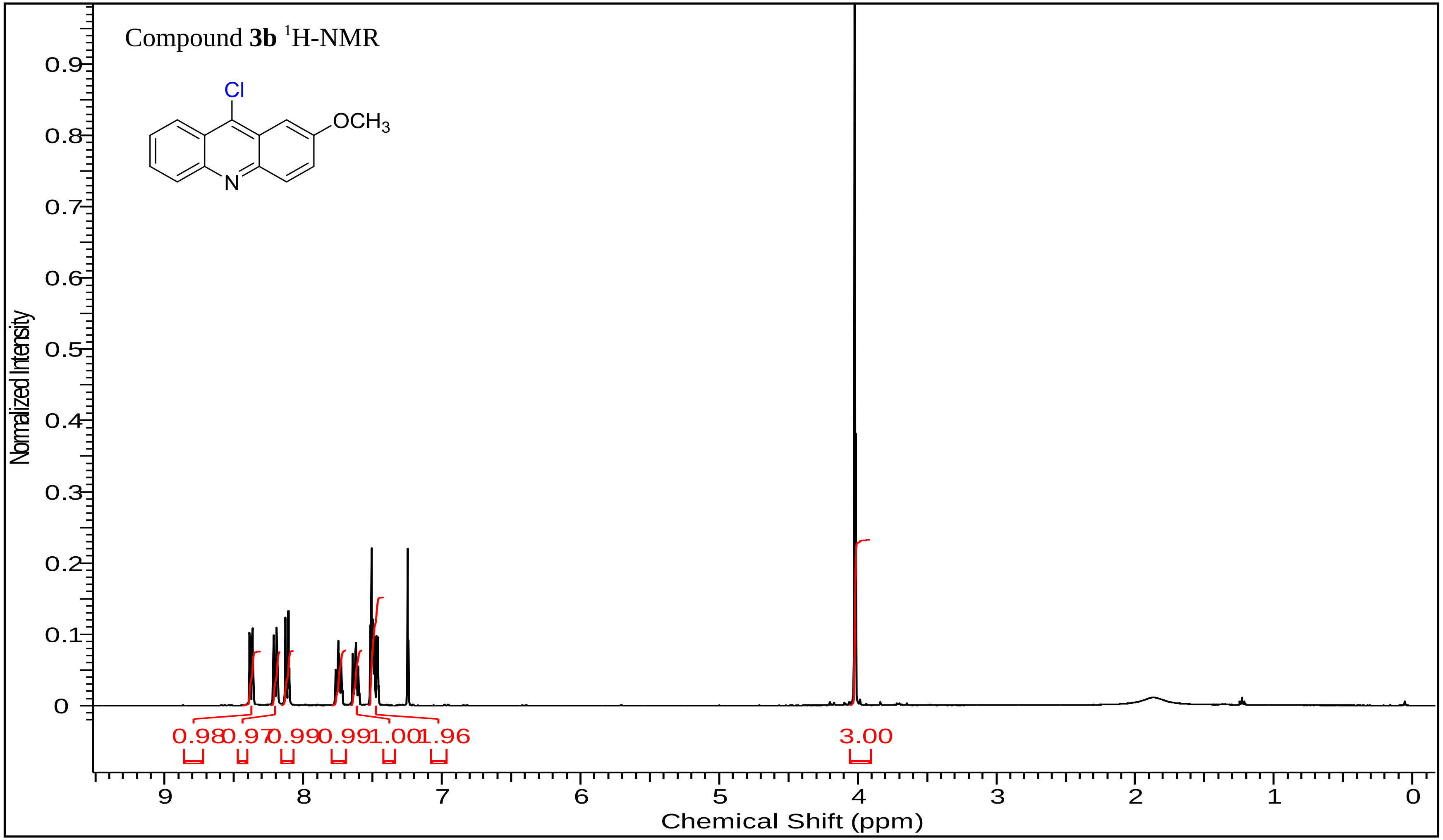
Compound 3b 1H-NMR
Cl
OCH3
N
Normalized Intensity
0.9
0.8
0.7
0.6
0.5
0.4
0.3
0.2
0.1
0
0.98
0.97
0.99
0.99
1.00
1.96
3.00
9
8
7
6
5
4
3
2
1
0
Chemical Shift (ppm)

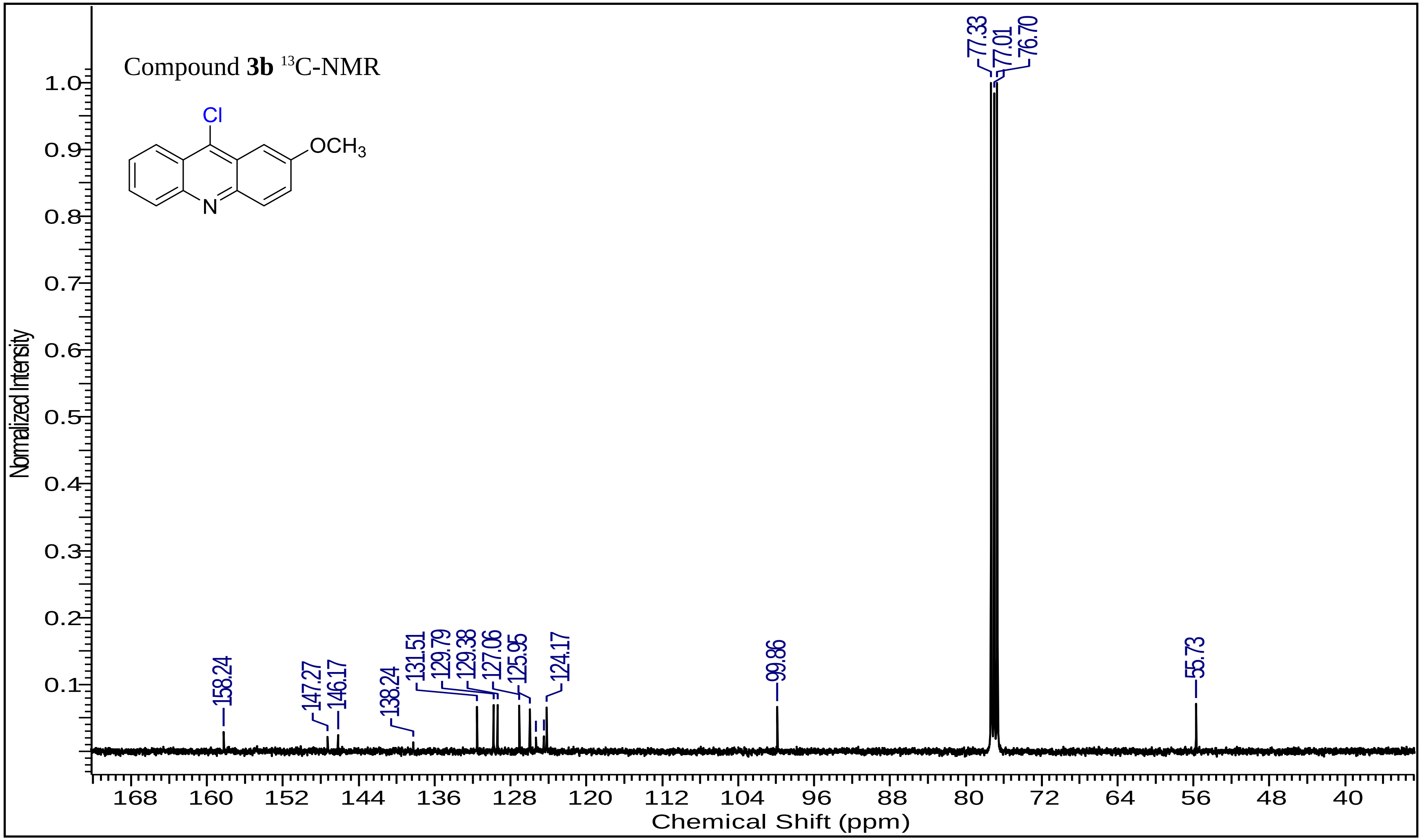

Compound 3b 13C-NMR
Cl
OCH3
N
Normalized Intensity
Chemical Shift (ppm)
158.24
147.27
146.17
138.24
131.51
129.79
129.38
127.06
125.95
124.17
99.86
77.33
77.01
76.70
55.73

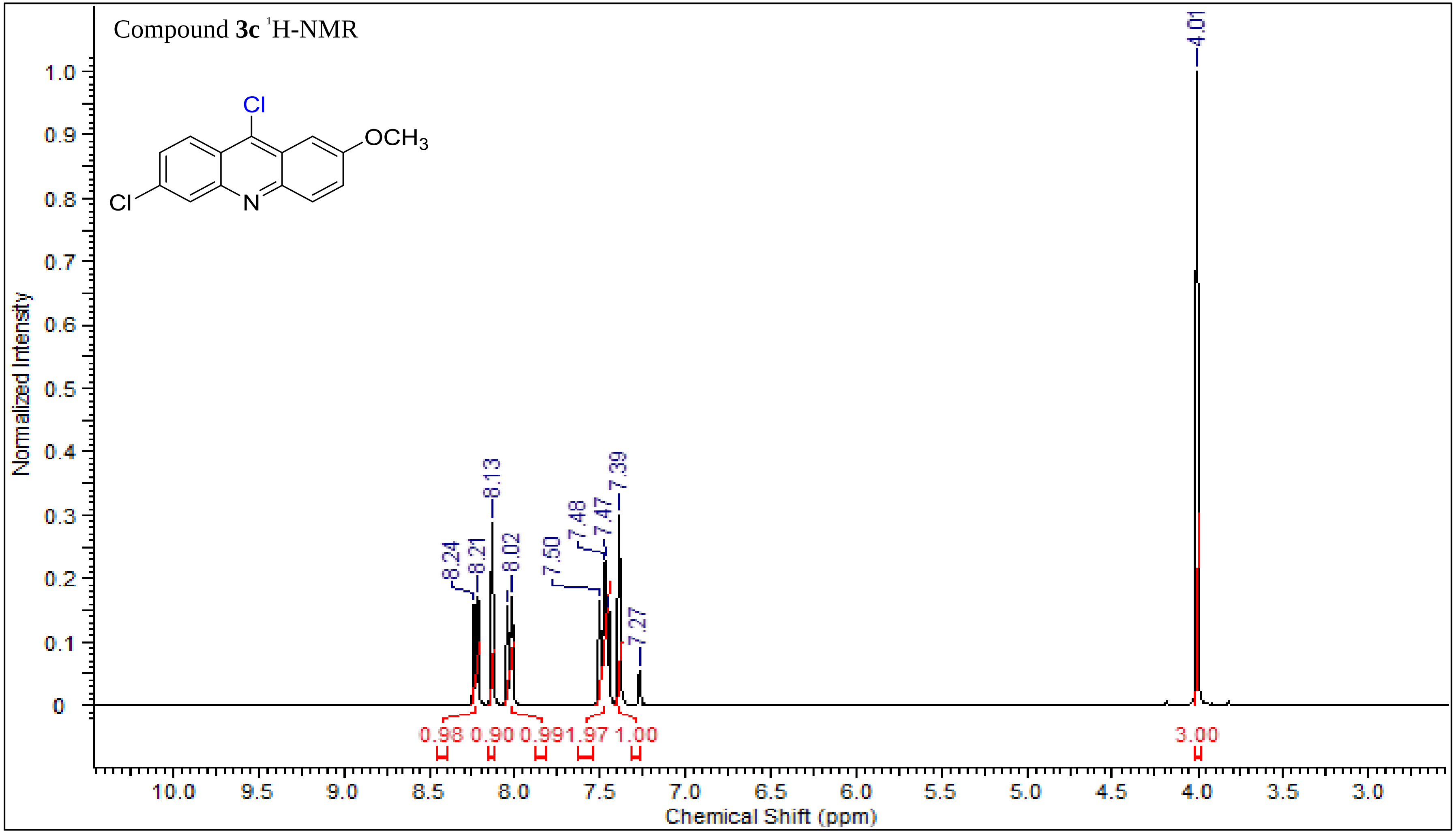
Compound 3c 1H-NMR
Cl
OCH3
Cl
N
Normalized Intensity
1.0
0.9
0.8
0.7
0.6
0.5
0.4
0.3
0.2
0.1
0
8.24
8.21
8.13
8.02
7.50
7.48
7.47
7.39
7.27
4.01
0.98
0.90
0.99
1.97
1.00
3.00
10.0
9.5
9.0
8.5
8.0
7.5
7.0
6.5
6.0
5.5
5.0
4.5
4.0
3.5
3.0
Chemical Shift (ppm)

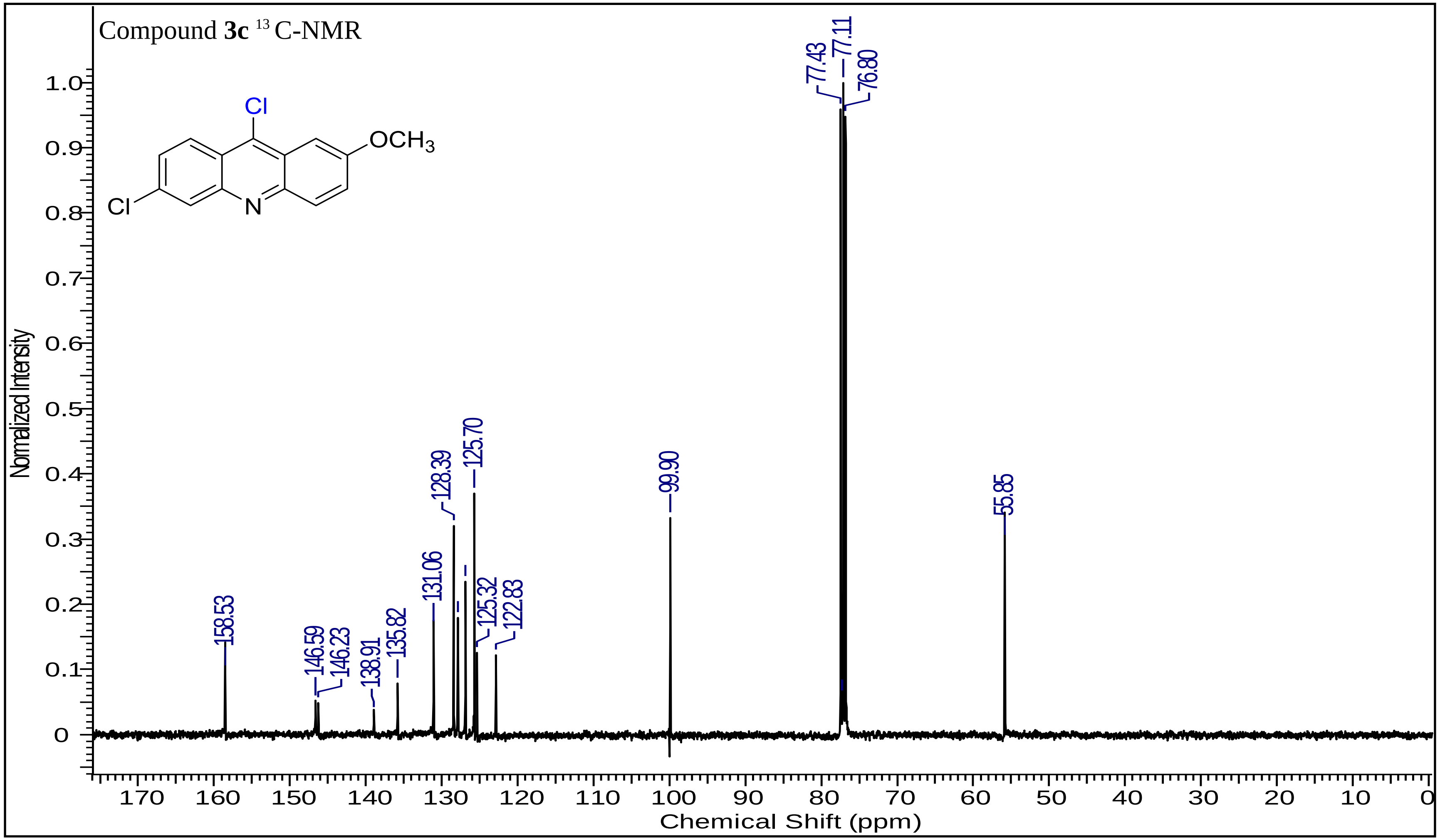
Compound 3c 13 C-NMR
Cl
OCH3
Cl
N
Normalized Intensity
1.0
0.9
0.8
0.7
0.6
0.5
0.4
0.3
0.2
0.1
0
158.53
146.59
146.23
138.91
135.82
131.06
128.39
125.70
125.32
122.83
99.90
77.43
77.11
76.80
55.85
170 160 150 140 130 120 110 100 90 80 70 60 50 40 30 20 10 0
Chemical Shift (ppm)

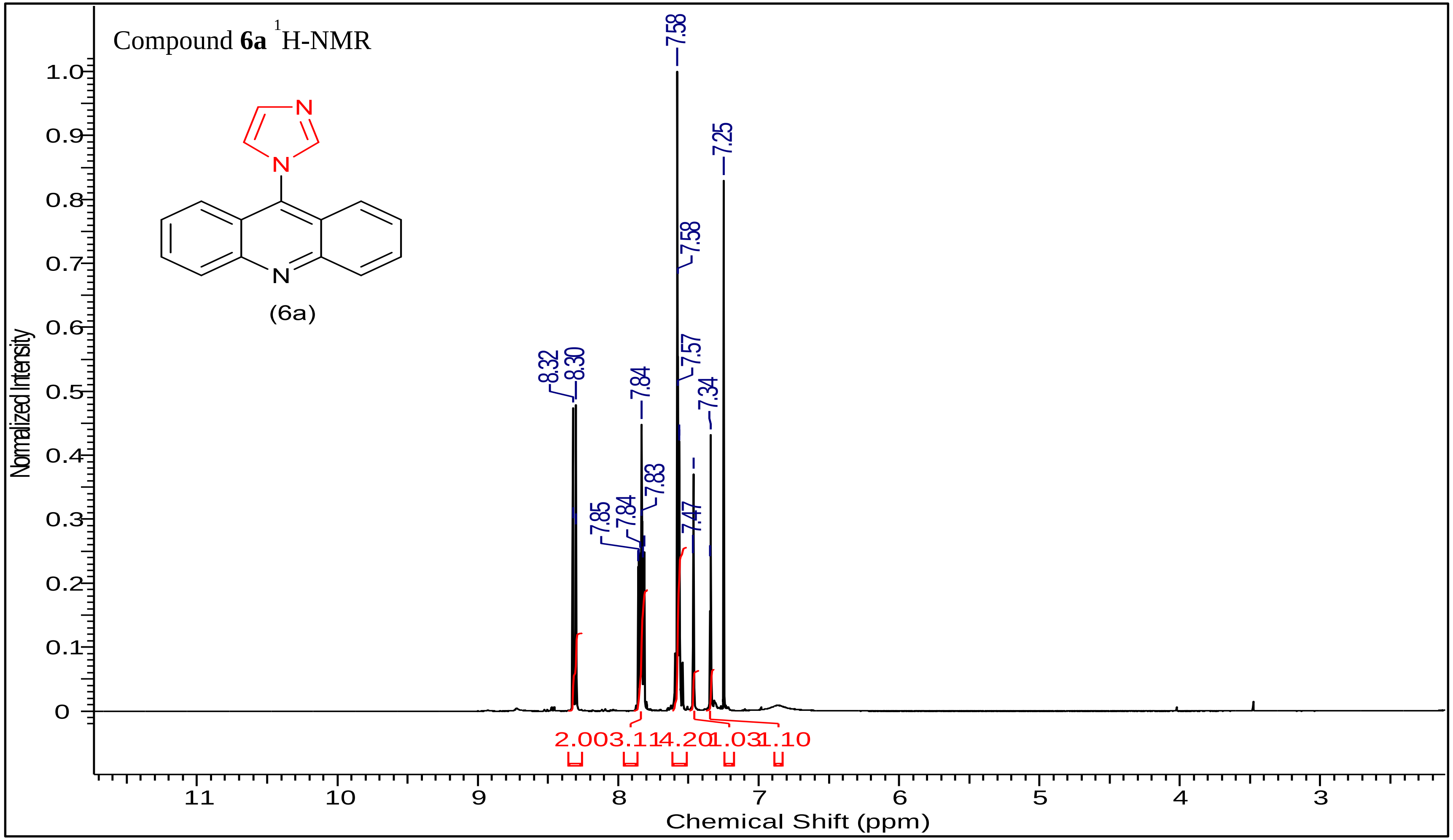
Compound 6a 1H-NMR
N
N
N
(6a)
Normalized Intensity
1.0
0.9
0.8
0.7
0.6
0.5
0.4
0.3
0.2
0.1
0
8.32
8.30
7.85
7.84
7.84
7.83
7.58
7.58
7.57
7.47
7.34
7.25
2.00
3.11
4.20
1.03
1.10
11
10
9
8
7
6
5
4
3
Chemical Shift (ppm)

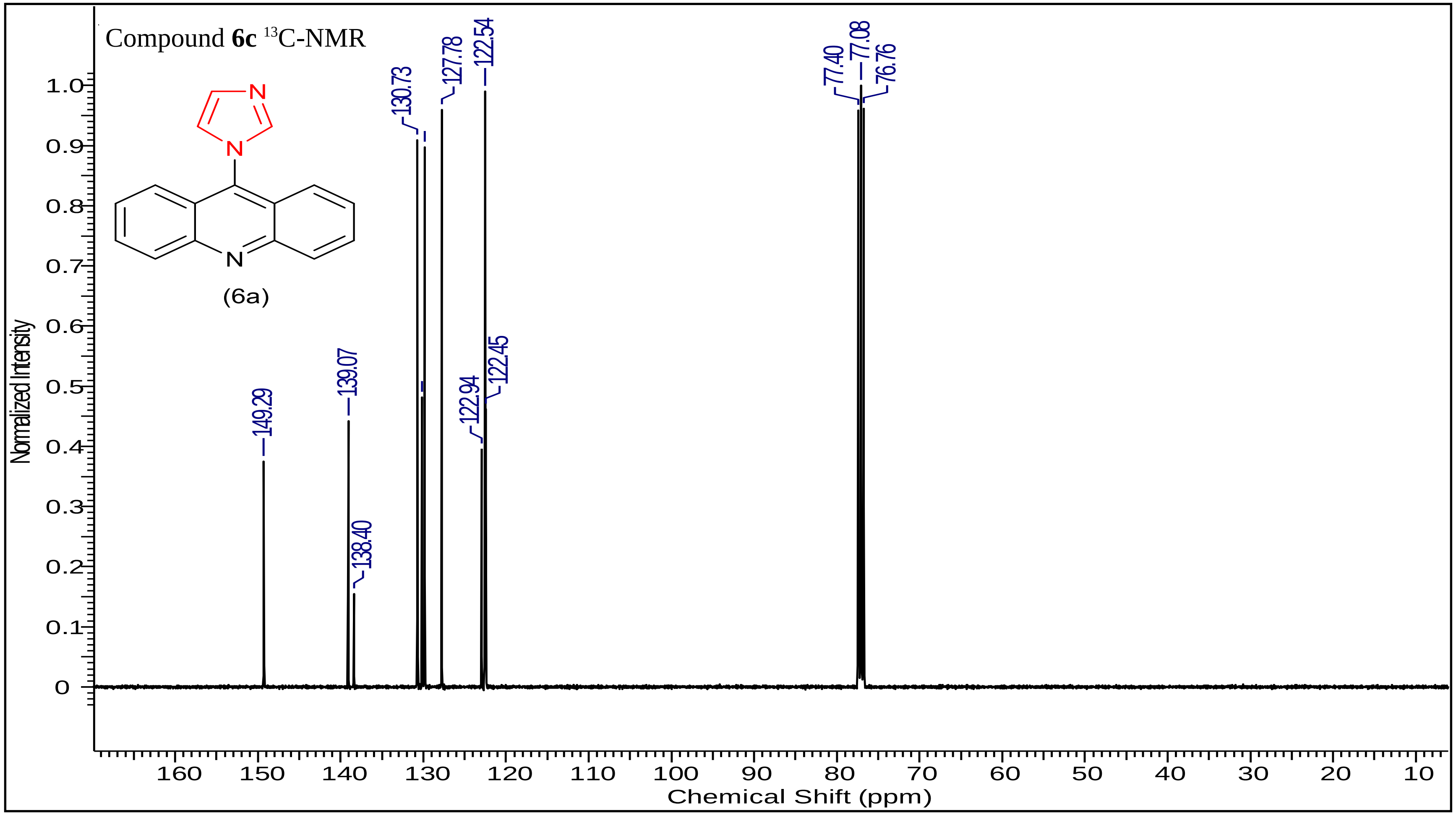
Compound 6c 13C-NMR
(6a)
149.29
139.07
138.40
130.73
127.78
122.94
122.54
122.45
77.40
77.08
76.76
Normalized Intensity
Chemical Shift (ppm)

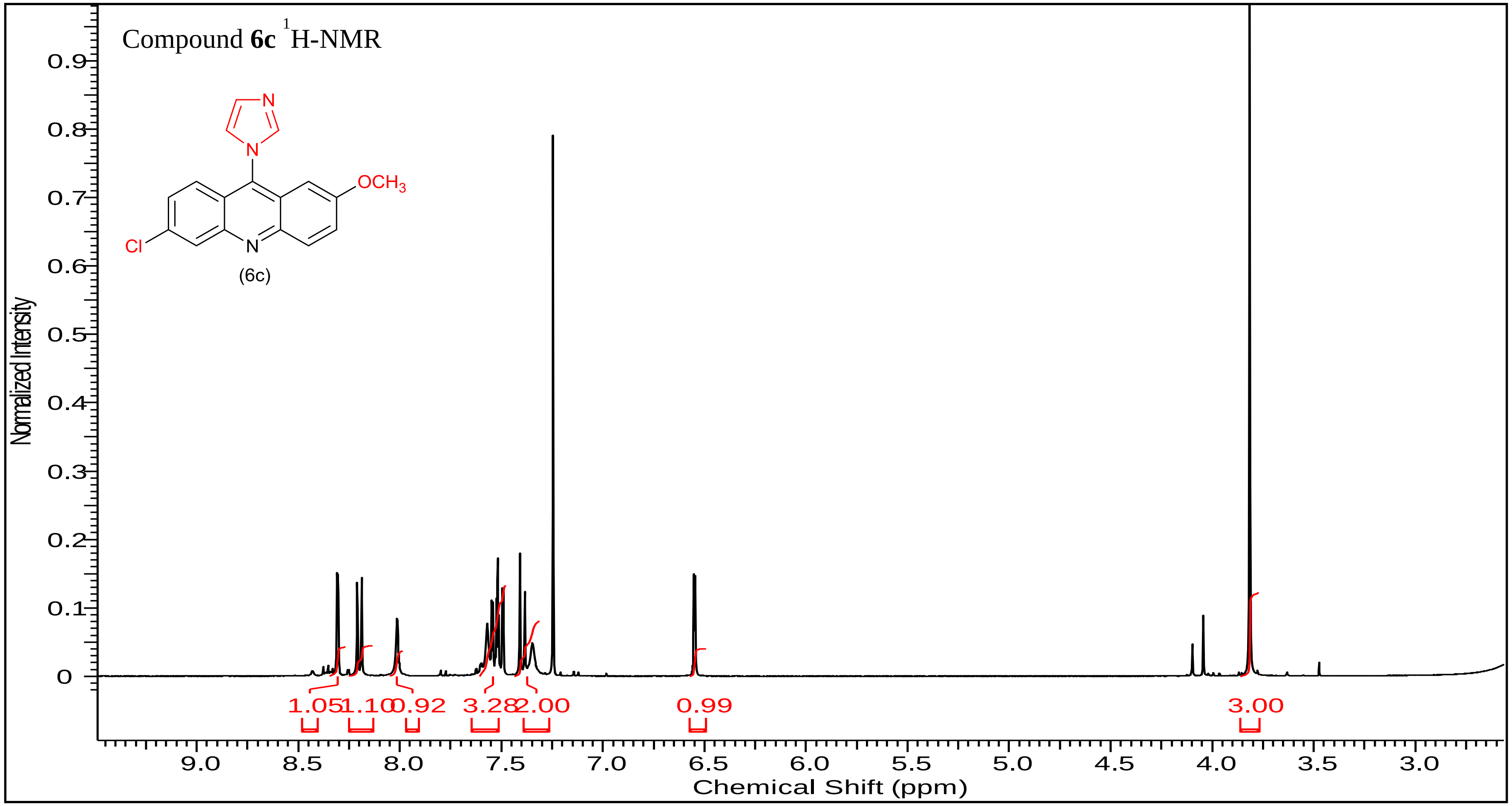
Compound 6c 1H-NMR
N
N
OCH3
Cl
N
(6c)
Normalized Intensity
0.9
0.8
0.7
0.6
0.5
0.4
0.3
0.2
0.1
0
1.05
1.10
0.92
3.28
2.00
0.99
3.00
9.0
8.5
8.0
7.5
7.0
6.5
6.0
5.5
5.0
4.5
4.0
3.5
3.0
Chemical Shift (ppm)

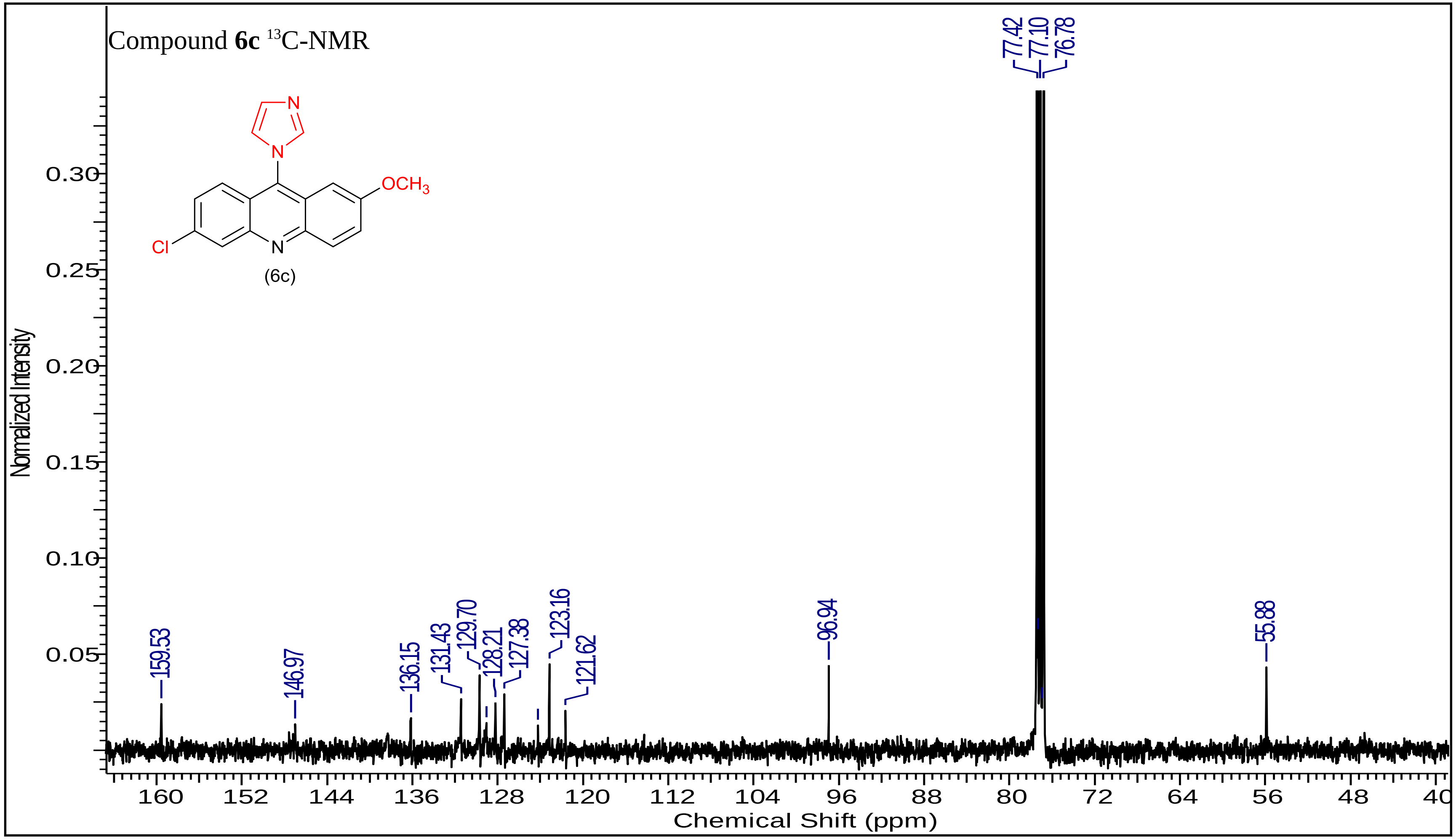

Compound 6c 13C-NMR
OCH3
Cl
N
(6c)
Normalized Intensity
Chemical Shift (ppm)
159.53
146.97
136.15
131.43
129.70
128.21
127.38
123.16
121.62
96.94
77.42
77.10
76.78
55.88

Compound **7** ^{1}H-NMR

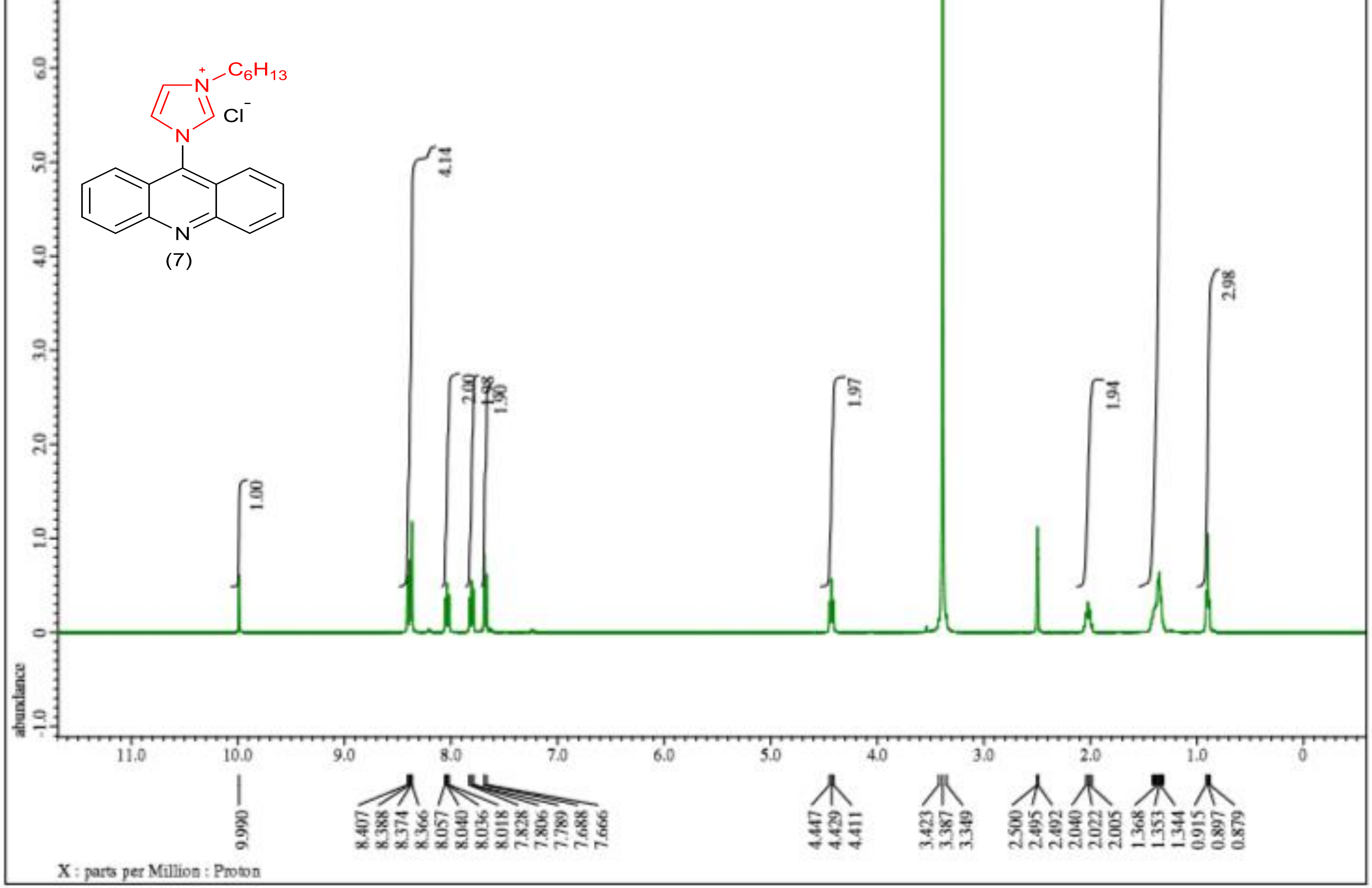

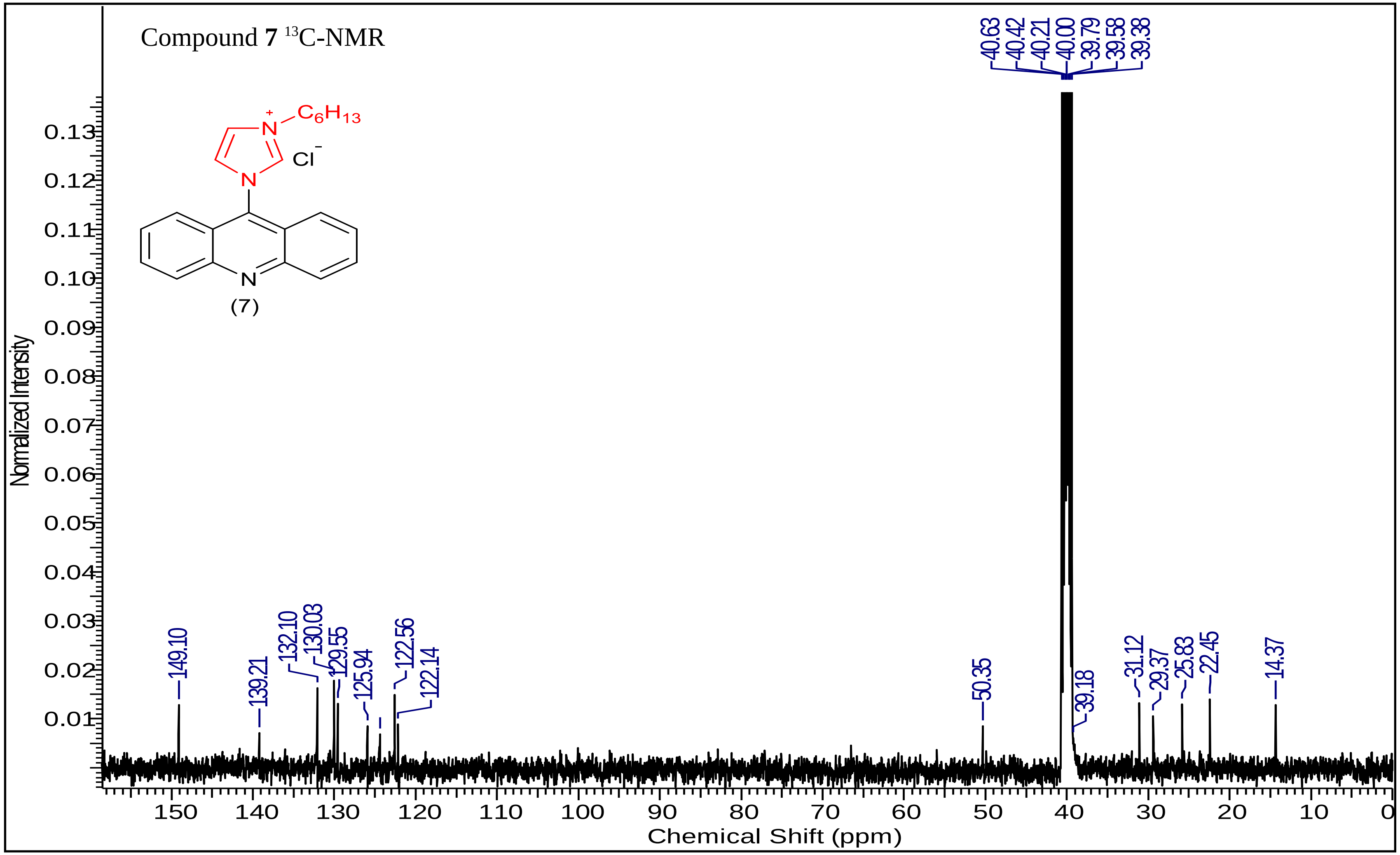

Compound 7 13C-NMR
C6H13
Cl
N
(7)
Normalized Intensity
Chemical Shift (ppm)
149.10
139.21
132.10
130.03
129.55
125.94
122.56
122.14
50.35
40.63
40.42
40.21
40.00
39.79
39.58
39.38
39.18
31.12
29.37
25.83
22.45
14.37

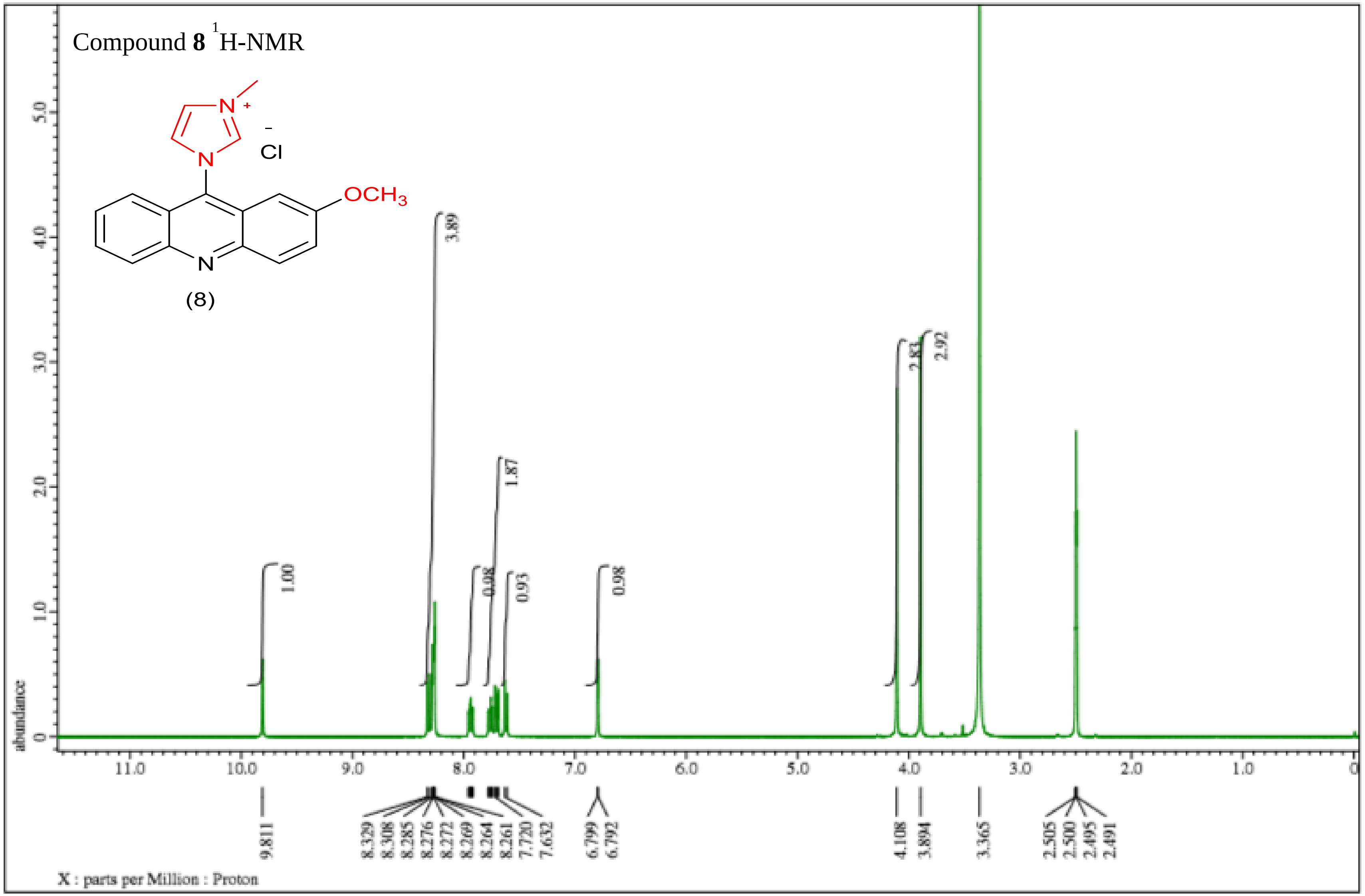
Compound 8 1H-NMR
OCH3
Cl
(8)
abundance
X : parts per Million : Proton
9.811
8.329
8.308
8.285
8.276
8.272
8.269
8.264
8.261
7.720
7.632
6.799
6.792
4.108
3.894
3.365
2.505
2.500
2.495
2.491
1.00
3.89
0.98
1.87
0.93
0.98
2.83
2.92

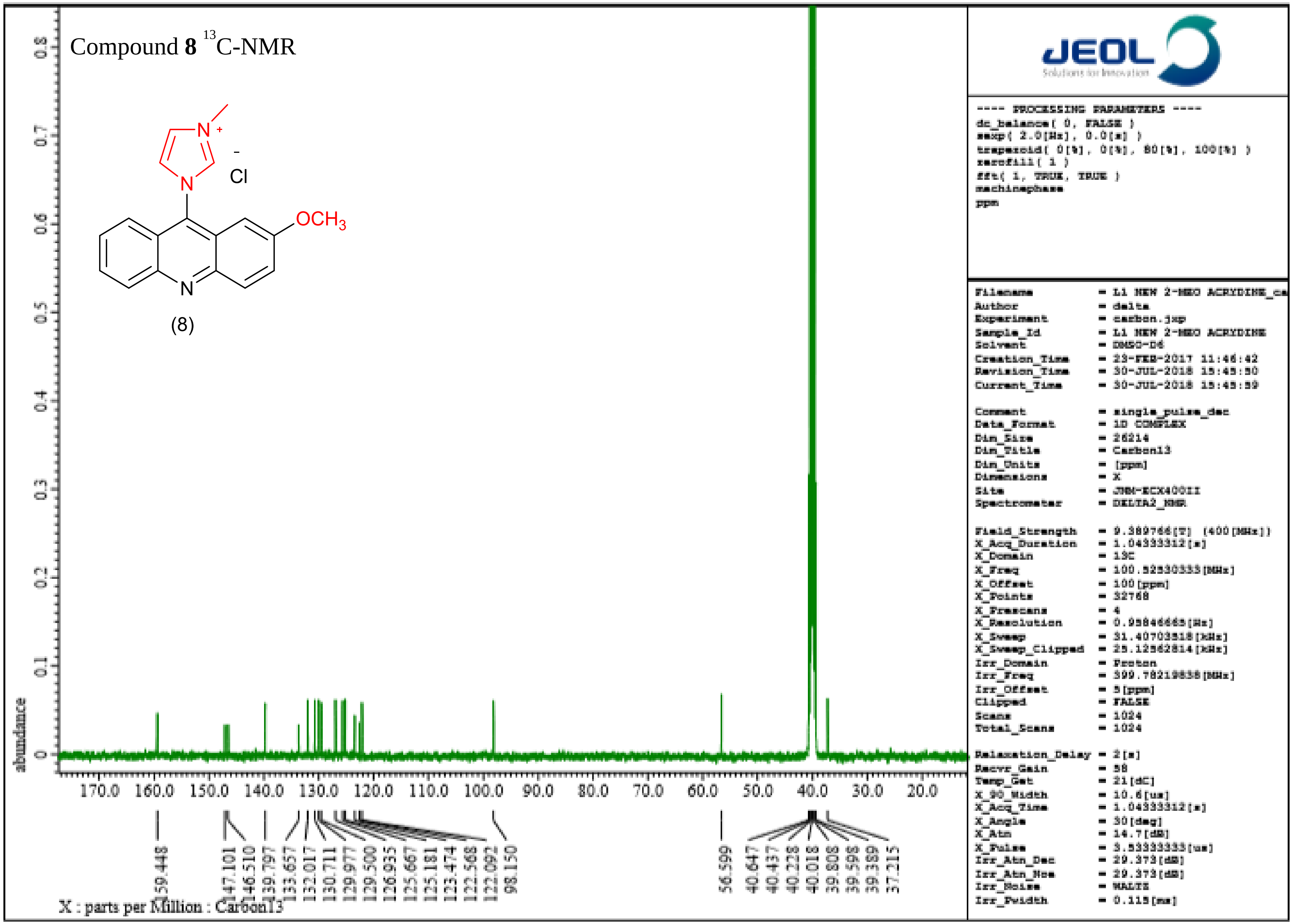
Compound 8 13C-NMR
N
N
Cl
OCH3
N
(8)
abundance
X : parts per Million : Carbon13
159.448
147.101
146.510
139.797
133.657
132.017
130.711
129.977
129.500
126.935
125.667
125.181
123.474
122.568
122.092
98.150
56.599
40.647
40.437
40.228
40.018
39.808
39.598
39.389
37.215
JEOL
---- PROCESSING PARAMETERS ----
dc_balance(0, FALSE)
sexp(2.0[Hz], 0.0[s])
trapezoid(0[%], 0[%], 80[%], 100[%])
zerofill(1)
fft(1, TRUE, TRUE)
machinephase
ppm
Filename = L1 NEW 2-MEO ACRYDINE_ca
Author = delta
Experiment = carbon.jxp
Sample_Id = L1 NEW 2-MEO ACRYDINE
Solvent = DMSO-D6
Creation_Time = 23-FEB-2017 11:46:42
Revision_Time = 30-JUL-2018 15:45:50
Current_Time = 30-JUL-2018 15:45:59
Comment = single_pulse_dec
Data_Format = 1D COMPLEX
Dim_Size = 26214
Dim_Title = Carbon13
Dim_Units = [ppm]
Dimensions = X
Site = JNM-ECX400II
Spectrometer = DELTA2_NMR
Field_Strength = 9.389766[T] (400[MHz])
X_Acq_Duration = 1.04333312[s]
X_Domain = 13C
X_Freq = 100.52530333[MHz]
X_Offset = 100[ppm]
X_Points = 32768
X_Prescans = 4
X_Resolution = 0.95846665[Hz]
X_Sweep = 31.40703518[kHz]
X_Sweep_Clipped = 25.12562814[kHz]
Irr_Domain = Proton
Irr_Freq = 399.78219838[MHz]
Irr_Offset = 5[ppm]
Clipped = FALSE
Scans = 1024
Total_Scans = 1024
Relaxation_Delay = 2[s]
Recvr_Gain = 58
Temp_Get = 21[dC]
X_90_Width = 10.6[us]
X_Acq_Time = 1.04333312[s]
X_Angle = 30[deg]
X_Atn = 14.7[dB]
X_Pulse = 3.53333333[us]
Irr_Atn_Dec = 29.373[dB]
Irr_Atn_Noe = 29.373[dB]
Irr_Noise = WALTZ
Irr_Pwidth = 0.115[ms]

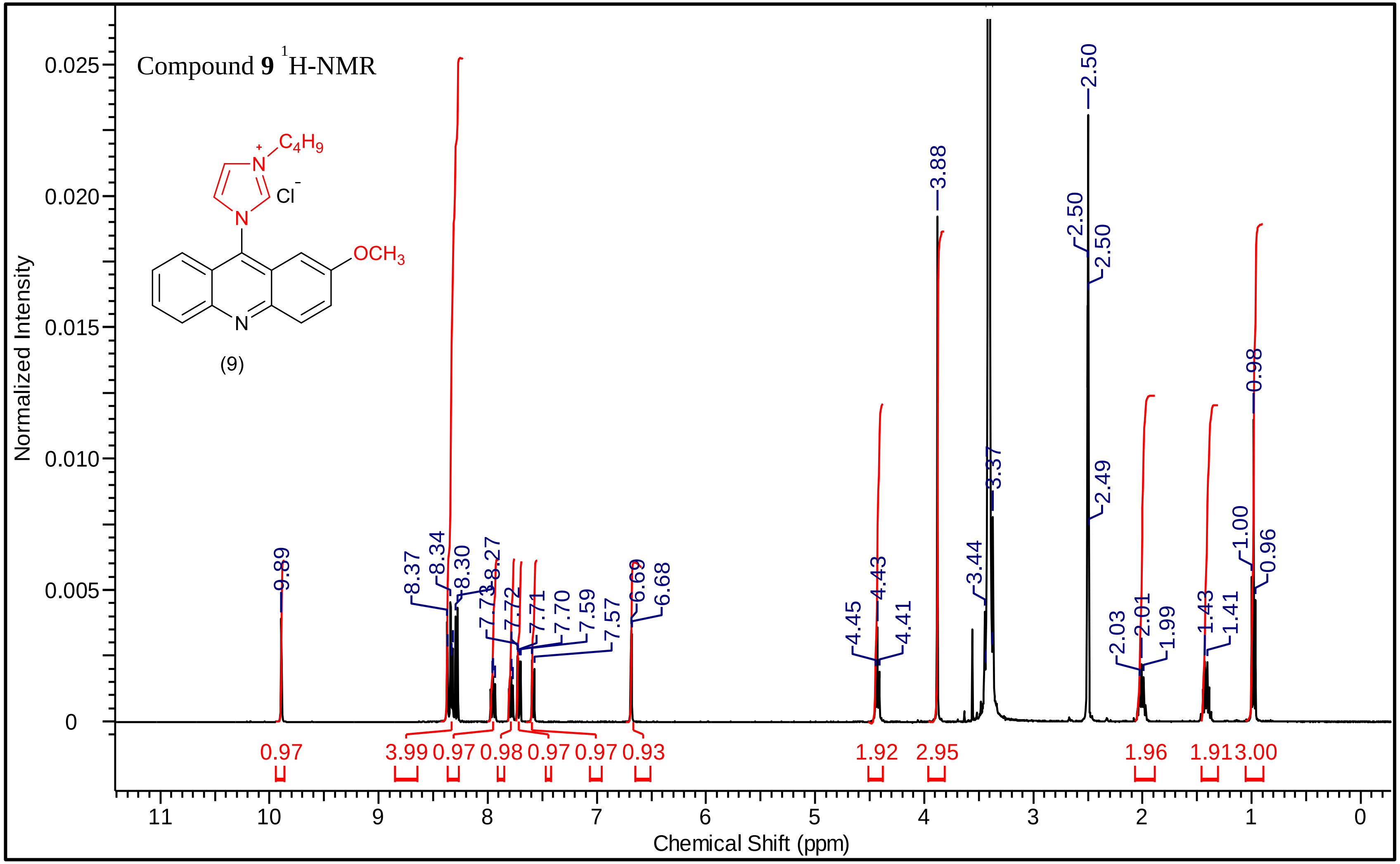

Compound 9 1H-NMR
(9)
Normalized Intensity
Chemical Shift (ppm)

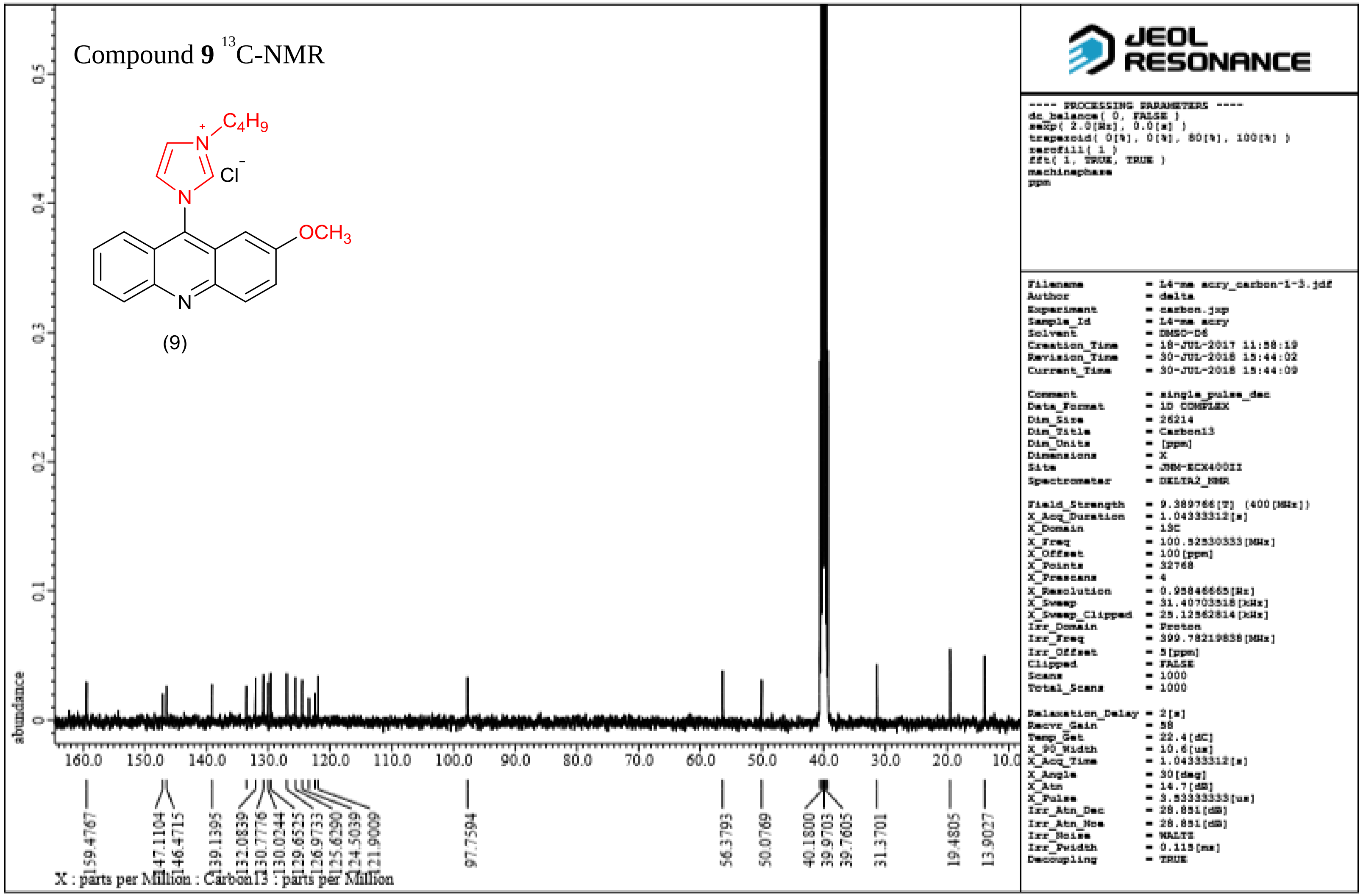
Compound 9 13C-NMR
C4H9
Cl-
N
N
OCH3
N
(9)
abundance
0
0.1
0.2
0.3
0.4
0.5
160.0
150.0
140.0
130.0
120.0
110.0
100.0
90.0
80.0
70.0
60.0
50.0
40.0
30.0
20.0
10.0
159.4767
147.1104
146.4715
139.1395
132.0839
130.7776
130.0244
129.6525
126.9733
125.6290
124.5039
121.9009
97.7594
56.3793
50.0769
40.1800
39.9703
39.7605
31.3701
19.4805
13.9027
X : parts per Million : Carbon13 : parts per Million
JEOL RESONANCE
---- PROCESSING PARAMETERS ----
dc_balance(0, FALSE)
sexp(2.0[Hz], 0.0[s])
trapezoid(0[%], 0[%], 80[%], 100[%])
zerofill(1)
fft(1, TRUE, TRUE)
machinephase
ppm
Filename = L4-me acry_carbon-1-3.jdf
Author = delta
Experiment = carbon.jxp
Sample_Id = L4-me acry
Solvent = DMSO-D6
Creation_Time = 18-JUL-2017 11:58:19
Revision_Time = 30-JUL-2018 15:44:02
Current_Time = 30-JUL-2018 15:44:09
Comment = single_pulse_dec
Data_Format = 1D COMPLEX
Dim_Size = 26214
Dim_Title = Carbon13
Dim_Units = [ppm]
Dimensions = X
Site = JNM-ECX400II
Spectrometer = DELTA2_NMR
Field_Strength = 9.389766[T] (400[MHz])
X_Acq_Duration = 1.04333312[s]
X_Domain = 13C
X_Freq = 100.52530333[MHz]
X_Offset = 100[ppm]
X_Points = 32768
X_Prescans = 4
X_Resolution = 0.95846665[Hz]
X_Sweep = 31.40703518[kHz]
X_Sweep_Clipped = 25.12562814[kHz]
Irr_Domain = Proton
Irr_Freq = 399.78219838[MHz]
Irr_Offset = 5[ppm]
Clipped = FALSE
Scans = 1000
Total_Scans = 1000
Relaxation_Delay = 2[s]
Recvr_Gain = 58
Temp_Get = 22.4[dC]
X_90_Width = 10.6[us]
X_Acq_Time = 1.04333312[s]
X_Angle = 30[deg]
X_Atn = 14.7[dB]
X_Pulse = 3.53333333[us]
Irr_Atn_Dec = 28.851[dB]
Irr_Atn_Noe = 28.851[dB]
Irr_Noise = WALTZ
Irr_Pwidth = 0.115[ms]
Decoupling = TRUE

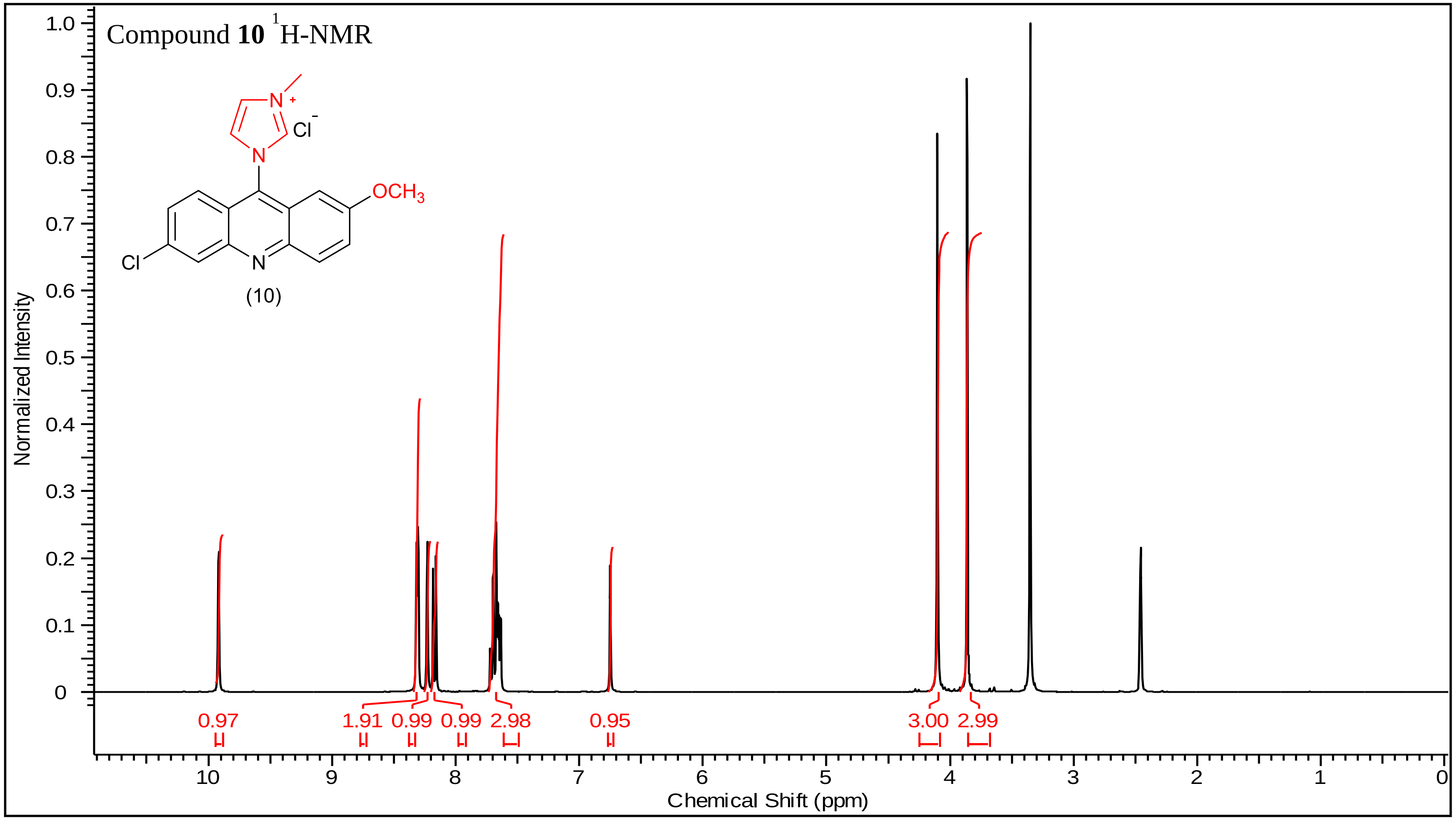

Compound 10 1H-NMR
N+
Cl-
N
OCH3
Cl
N
(10)
Normalized Intensity
0.97
1.91
0.99
0.99
2.98
0.95
3.00
2.99
Chemical Shift (ppm)

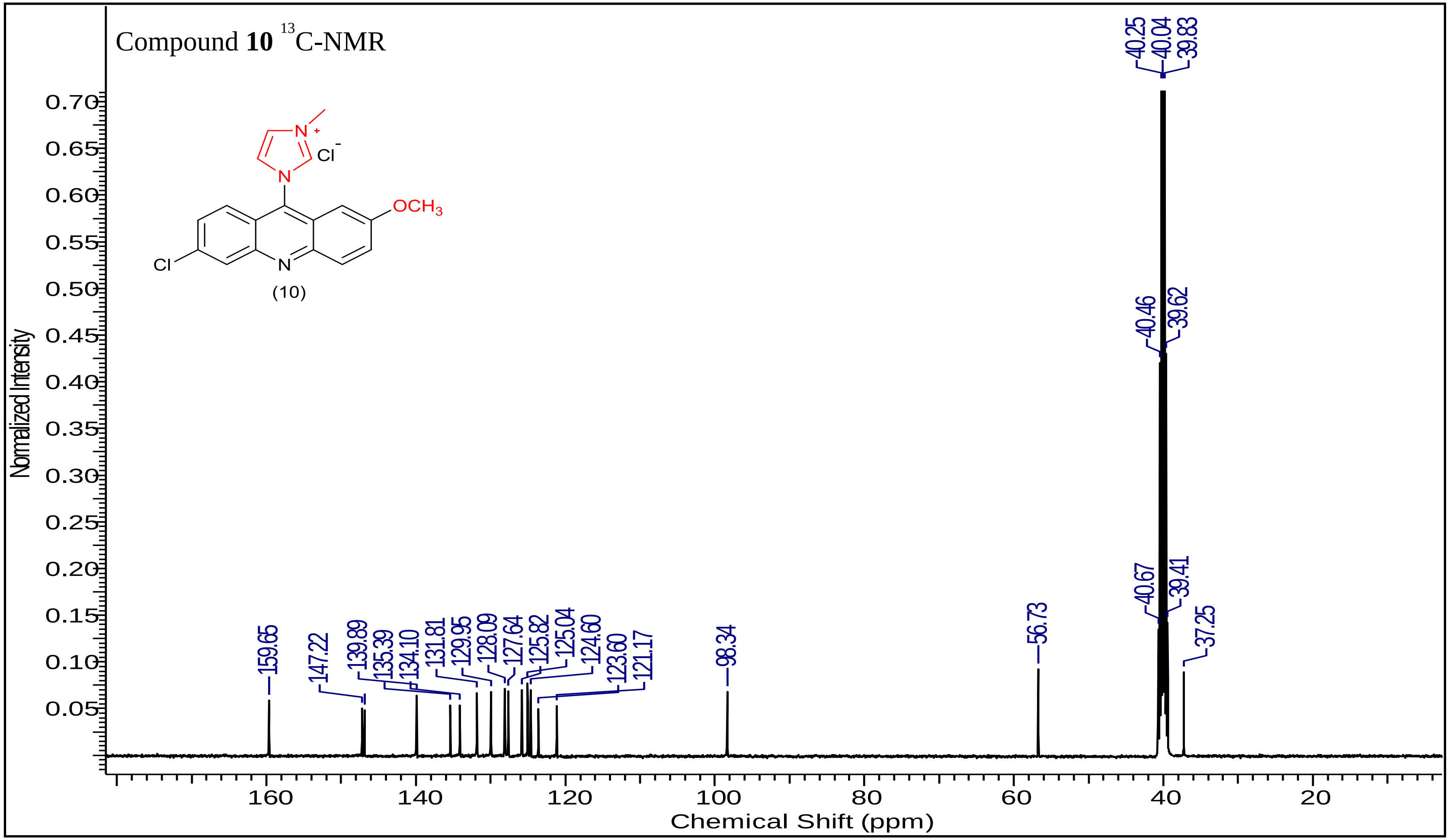

Compound **10** ^{13}C-NMR
Cl
N
N
N^+
Cl^-
OCH_3
(10)
Normalized Intensity
Chemical Shift (ppm)
159.65
147.22
139.89
135.39
134.10
131.81
129.95
128.09
127.64
125.82
125.04
124.60
123.60
121.17
98.34
56.73
40.67
40.46
40.25
40.04
39.83
39.62
39.41
37.25

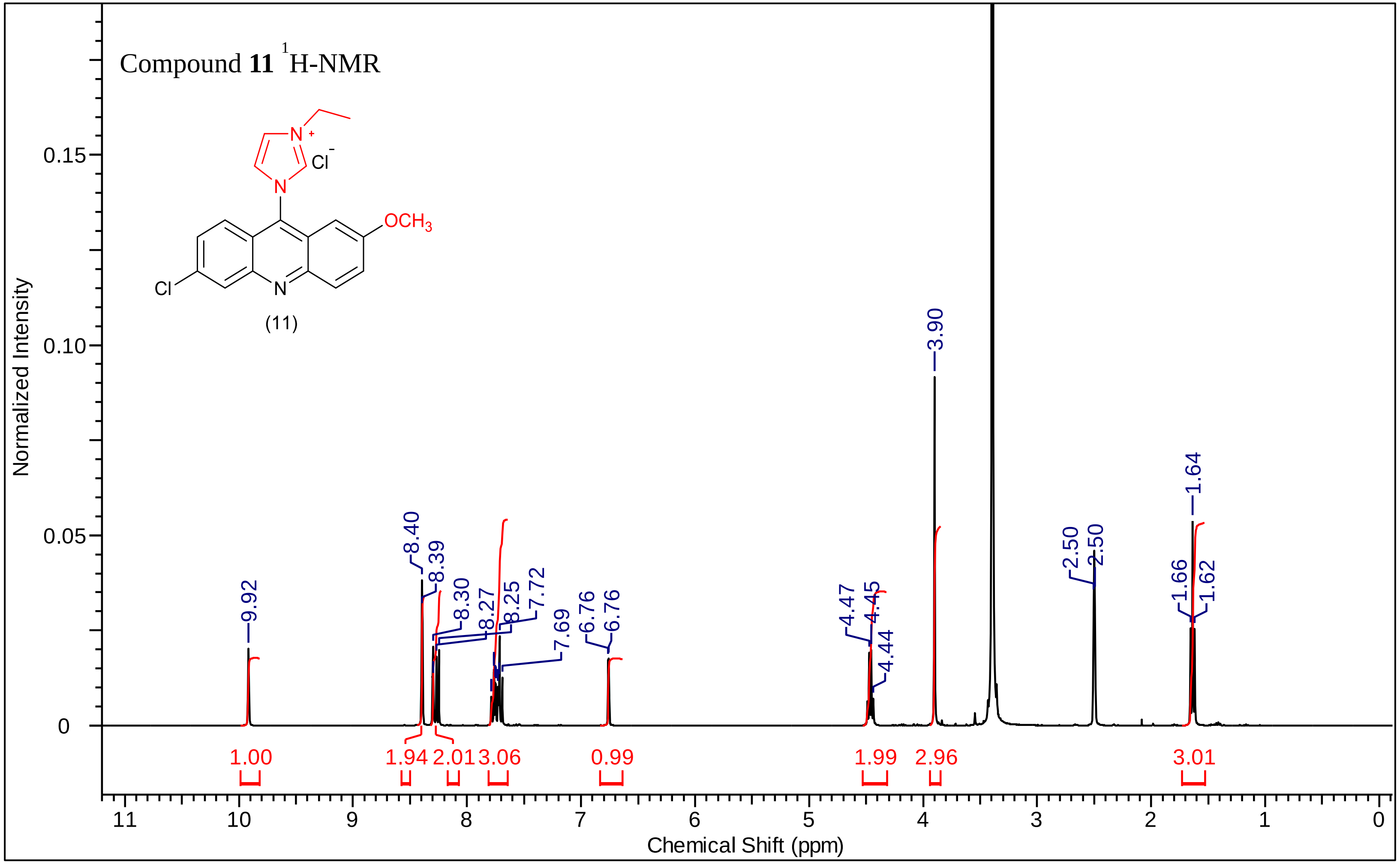

Compound 11 1H-NMR
N+
Cl-
N
OCH3
Cl
N
(11)
Normalized Intensity
0.15
0.10
0.05
0
9.92
8.40
8.39
8.30
8.27
8.25
7.72
7.69
6.76
6.76
4.47
4.45
4.44
3.90
2.50
2.50
1.66
1.64
1.62
1.00
1.94
2.01
3.06
0.99
1.99
2.96
3.01
11
10
9
8
7
6
5
4
3
2
1
0
Chemical Shift (ppm)

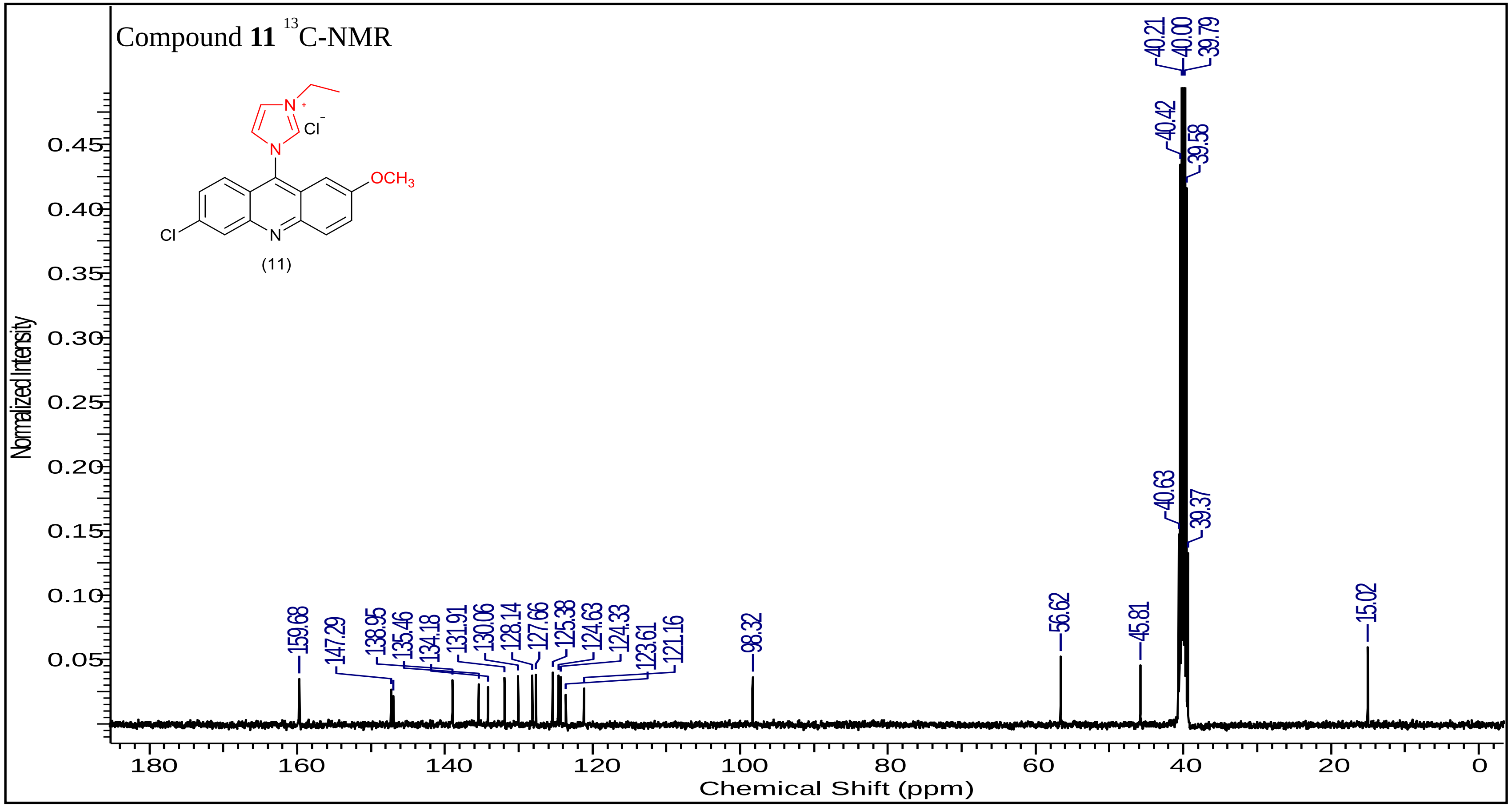
Compound 11 13C-NMR
(11)
Normalized Intensity
Chemical Shift (ppm)
159.68
147.29
138.95
135.46
134.18
131.91
130.06
128.14
127.66
125.38
124.63
124.33
123.61
121.16
98.32
56.62
45.81
40.63
40.42
40.21
40.00
39.79
39.58
39.37
15.02

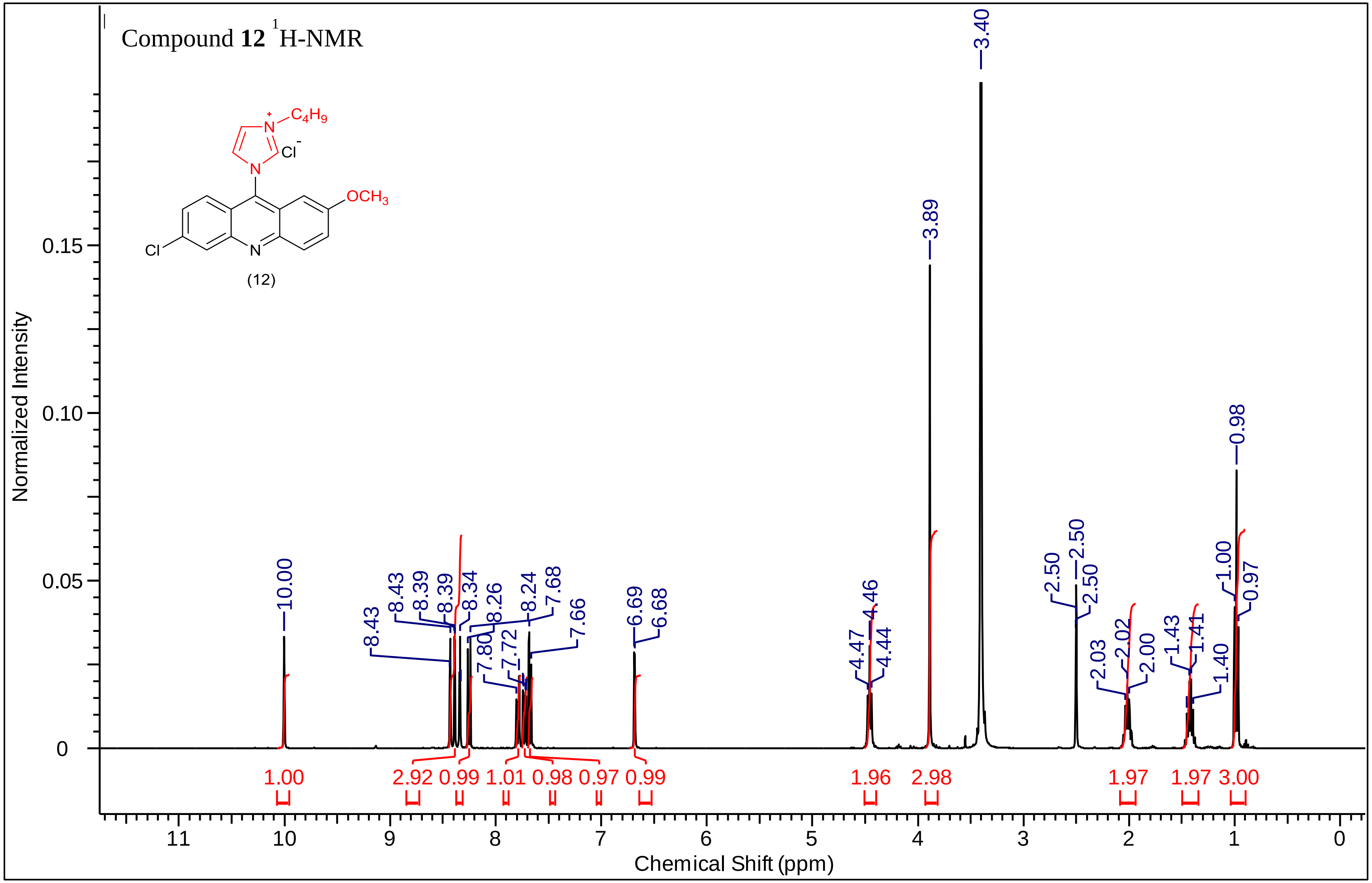
Compound 12 1H-NMR
(12)
Normalized Intensity
Chemical Shift (ppm)
10.00
8.43
8.43
8.39
8.39
8.34
8.26
8.24
7.80
7.72
7.68
7.66
6.69
6.68
4.47
4.46
4.44
3.89
3.40
2.50
2.50
2.50
2.03
2.02
2.00
1.43
1.41
1.40
1.00
0.98
0.97
1.00
2.92
0.99
1.01
0.98
0.97
0.99
1.96
2.98
1.97
1.97
3.00

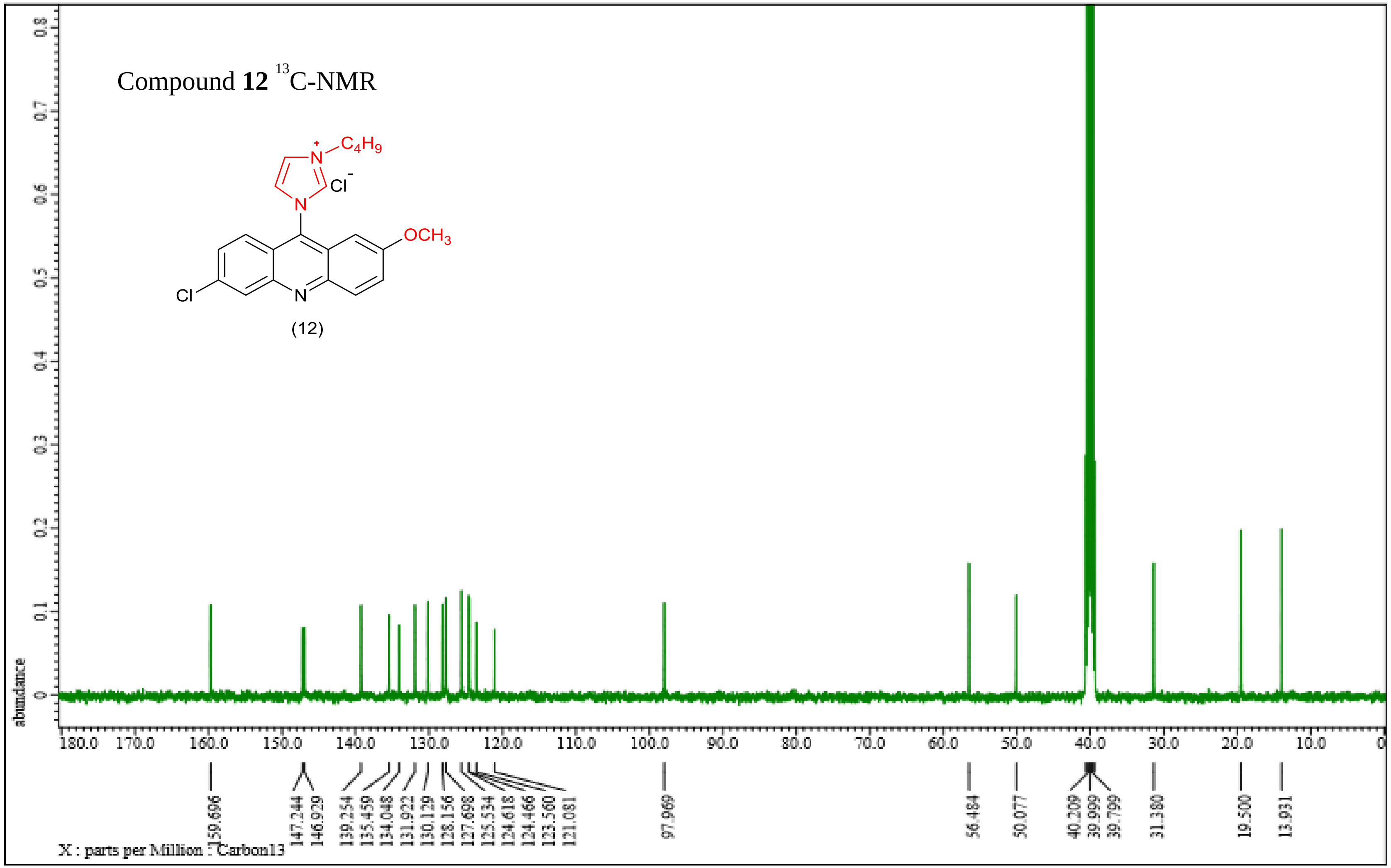
Compound 12 ^{13}C-NMR
C4H9
Cl-
OCH3
Cl
N
(12)
abundance
X : parts per Million : Carbon13
159.696
147.244
146.929
139.254
135.459
134.048
131.922
130.129
128.156
127.698
125.534
124.618
124.466
123.560
121.081
97.969
56.484
50.077
40.209
39.999
39.799
31.380
19.500
13.931

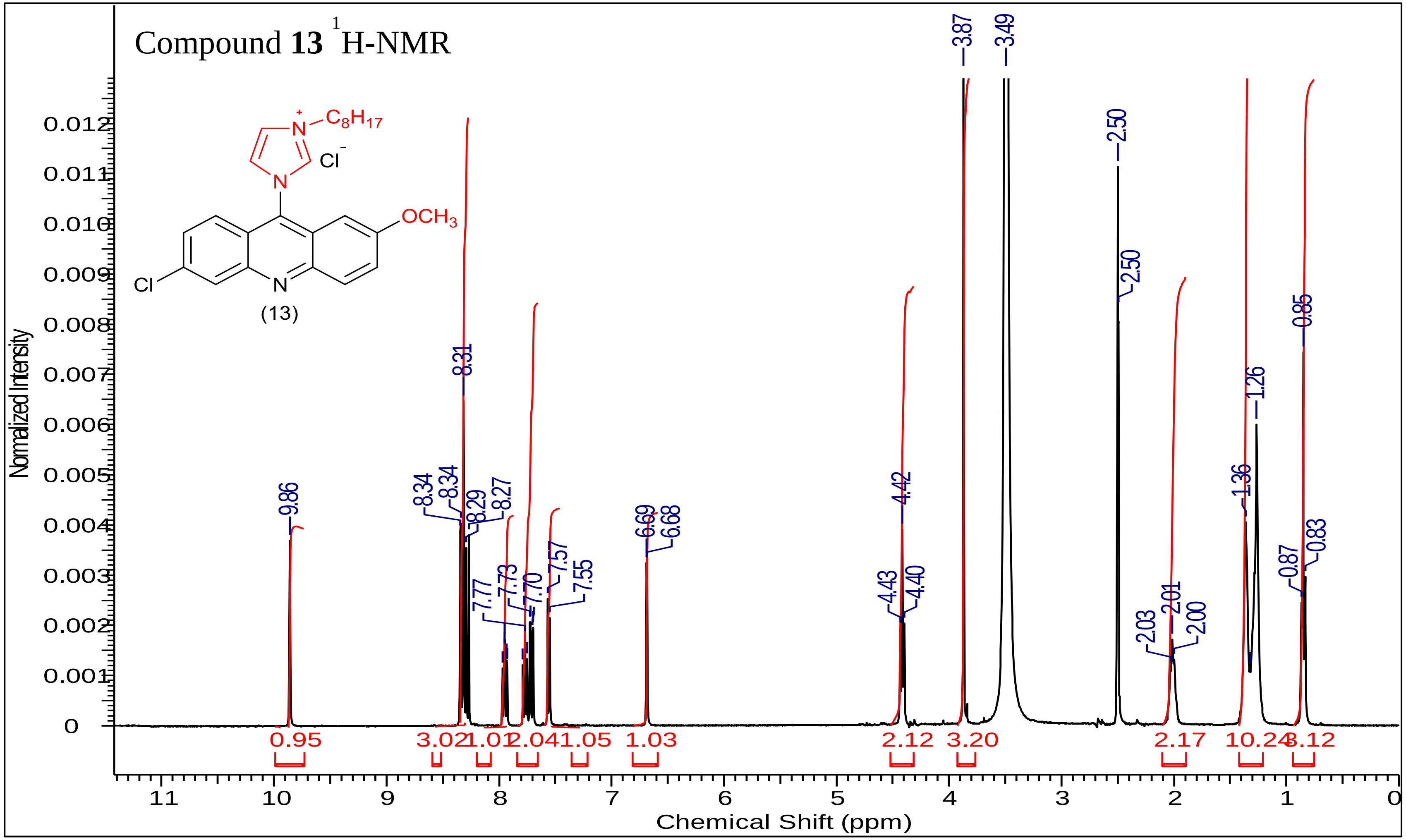
Compound 13 1H-NMR
C8H17
Cl-
OCH3
Cl
N
(13)
Normalized Intensity
Chemical Shift (ppm)
9.86
8.34
8.34
8.31
8.29
8.27
7.77
7.73
7.70
7.57
7.55
6.69
6.68
4.43
4.42
4.40
3.87
3.49
2.50
2.50
2.03
2.01
2.00
1.36
1.26
0.87
0.85
0.83
0.95
3.02
1.01
2.04
1.05
1.03
2.12
3.20
2.17
10.24
3.12

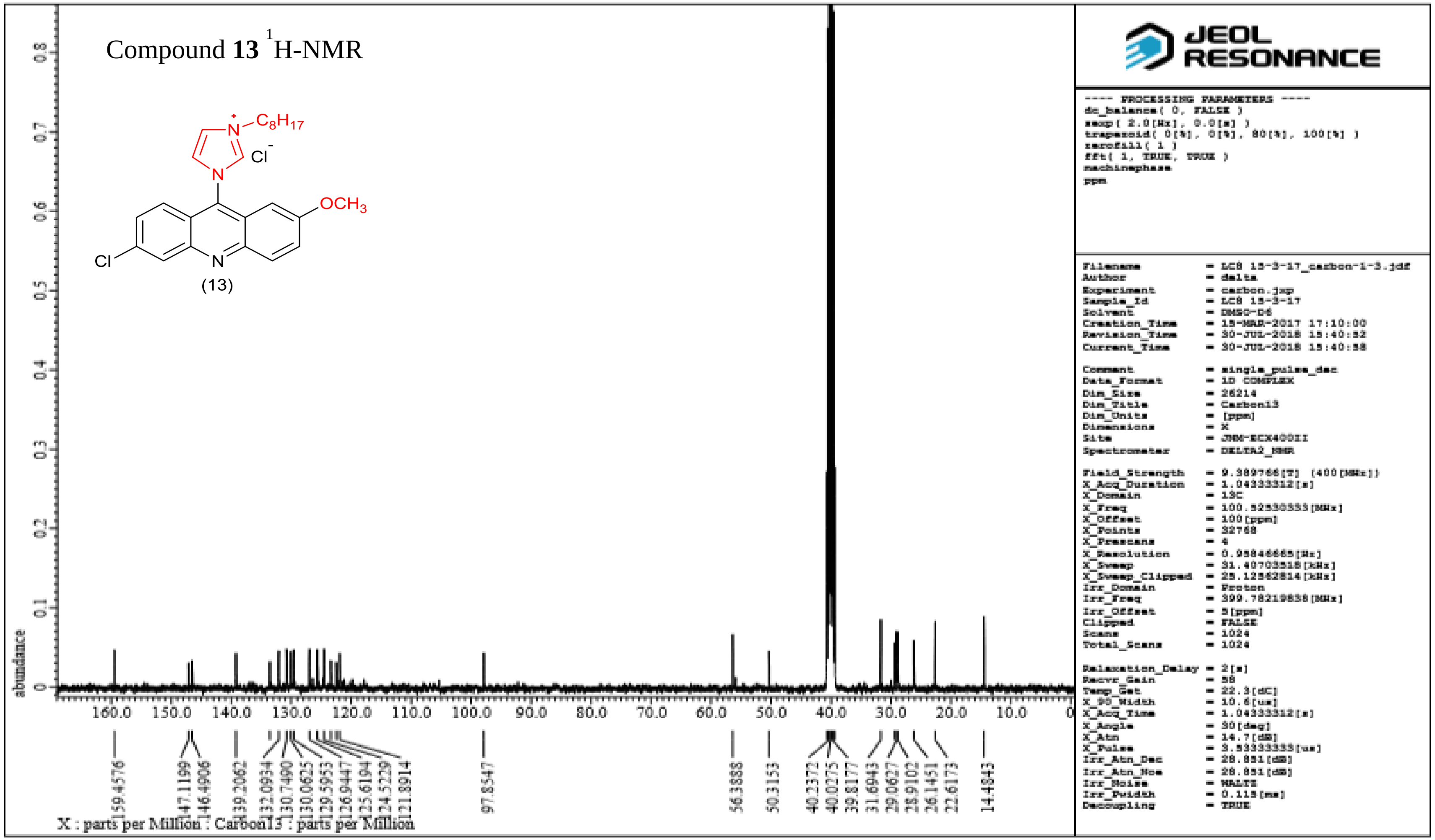
Compound 13 1H-NMR
C8H17
Cl-
OCH3
Cl
N
(13)
159.4576
147.1199
146.4906
139.2062
132.0934
130.7490
130.0625
129.5953
126.9447
125.6194
124.5229
121.8914
97.8547
56.3888
50.3153
40.2372
40.0275
39.8177
31.6943
29.0627
28.9102
26.1451
22.6173
14.4843
X : parts per Million : Carbon13 : parts per Million
abundance
JEOL RESONANCE

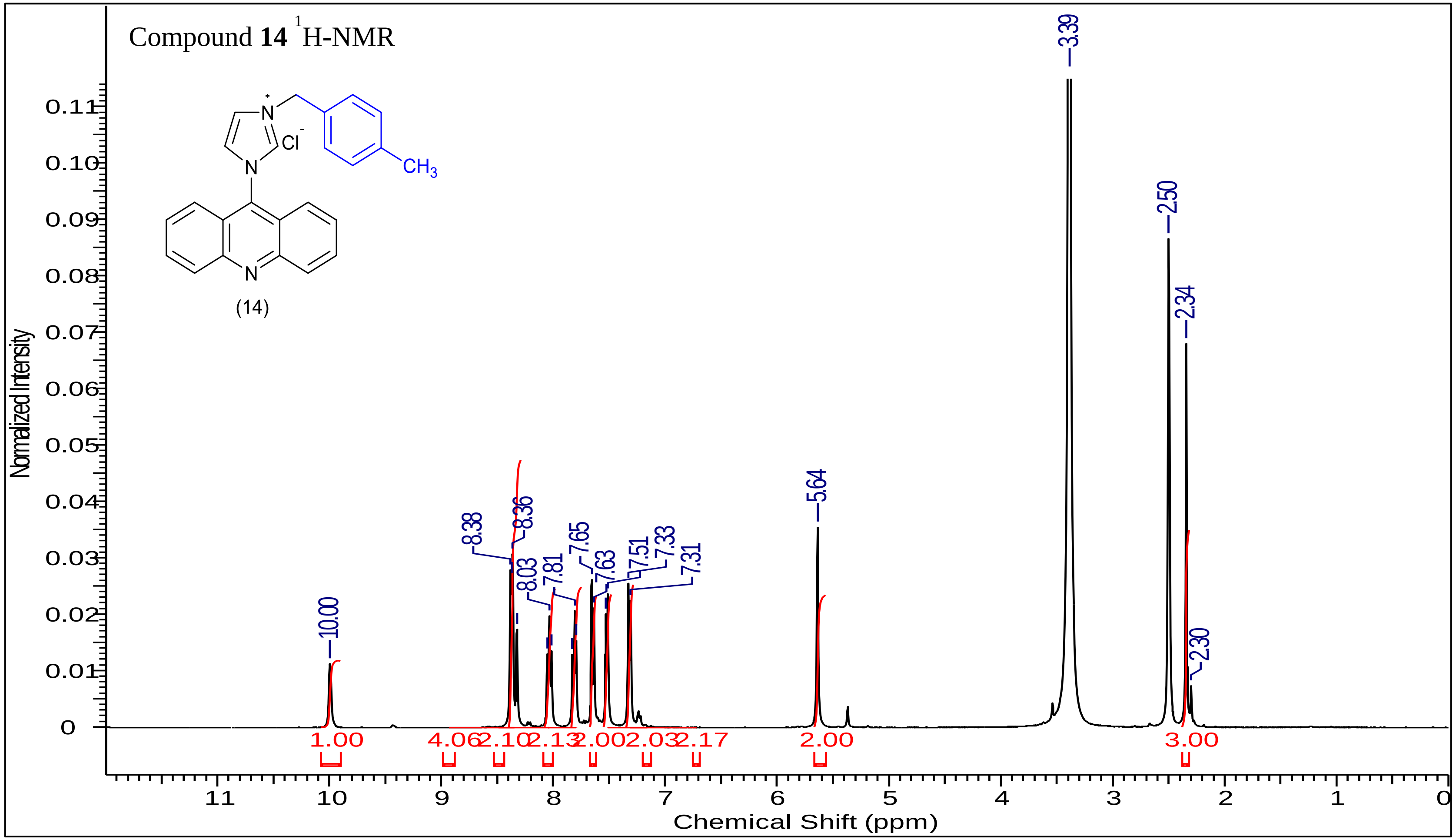
Compound 14 1H-NMR
Cl-
N
CH3
(14)
Normalized Intensity
Chemical Shift (ppm)
10.00
8.38
8.36
8.03
7.81
7.65
7.63
7.51
7.33
7.31
5.64
3.39
2.50
2.34
2.30
1.00
4.06
2.10
2.13
2.00
2.03
2.17
2.00
3.00

Compound **14** ^{1}H-NMR

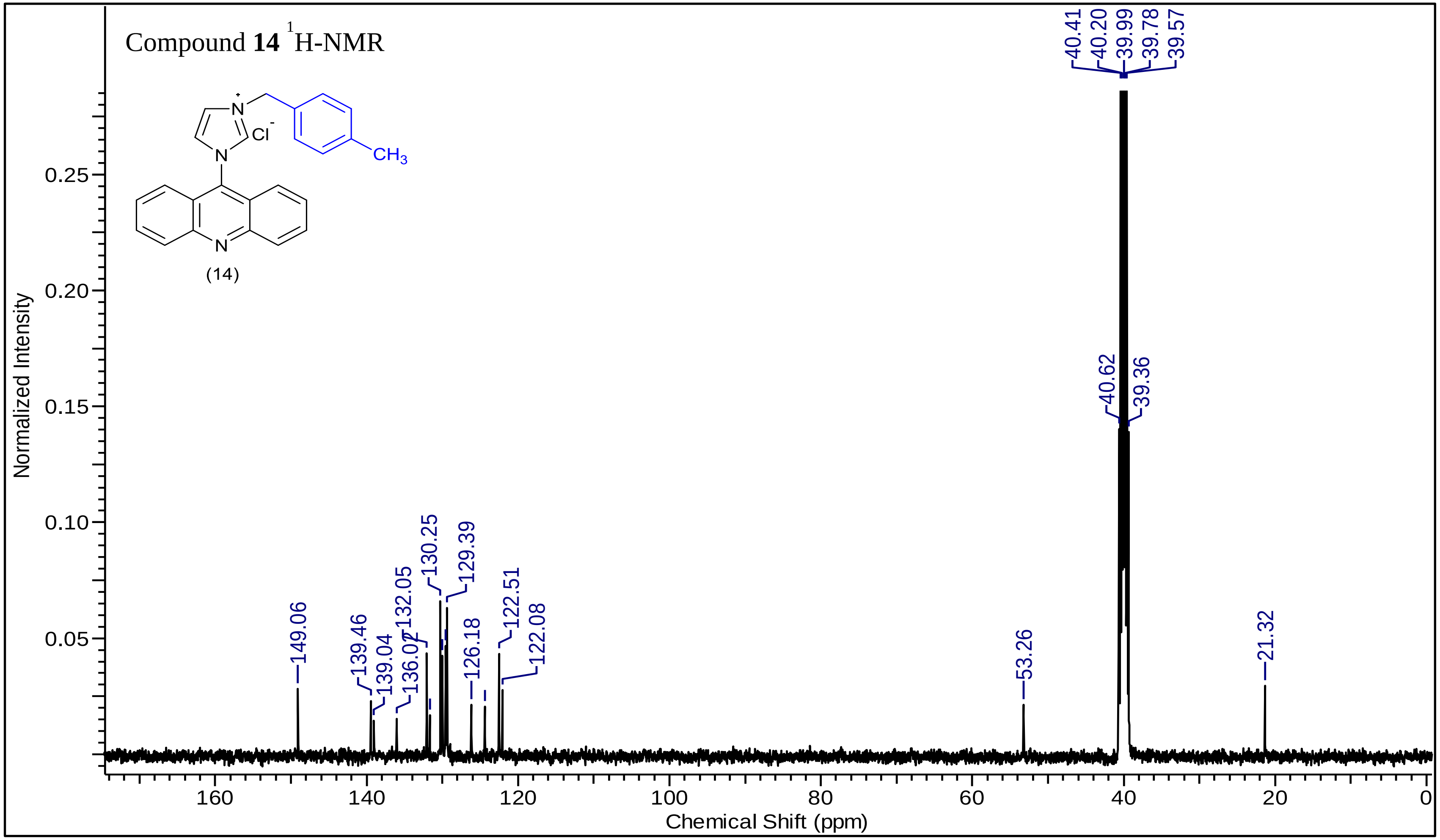

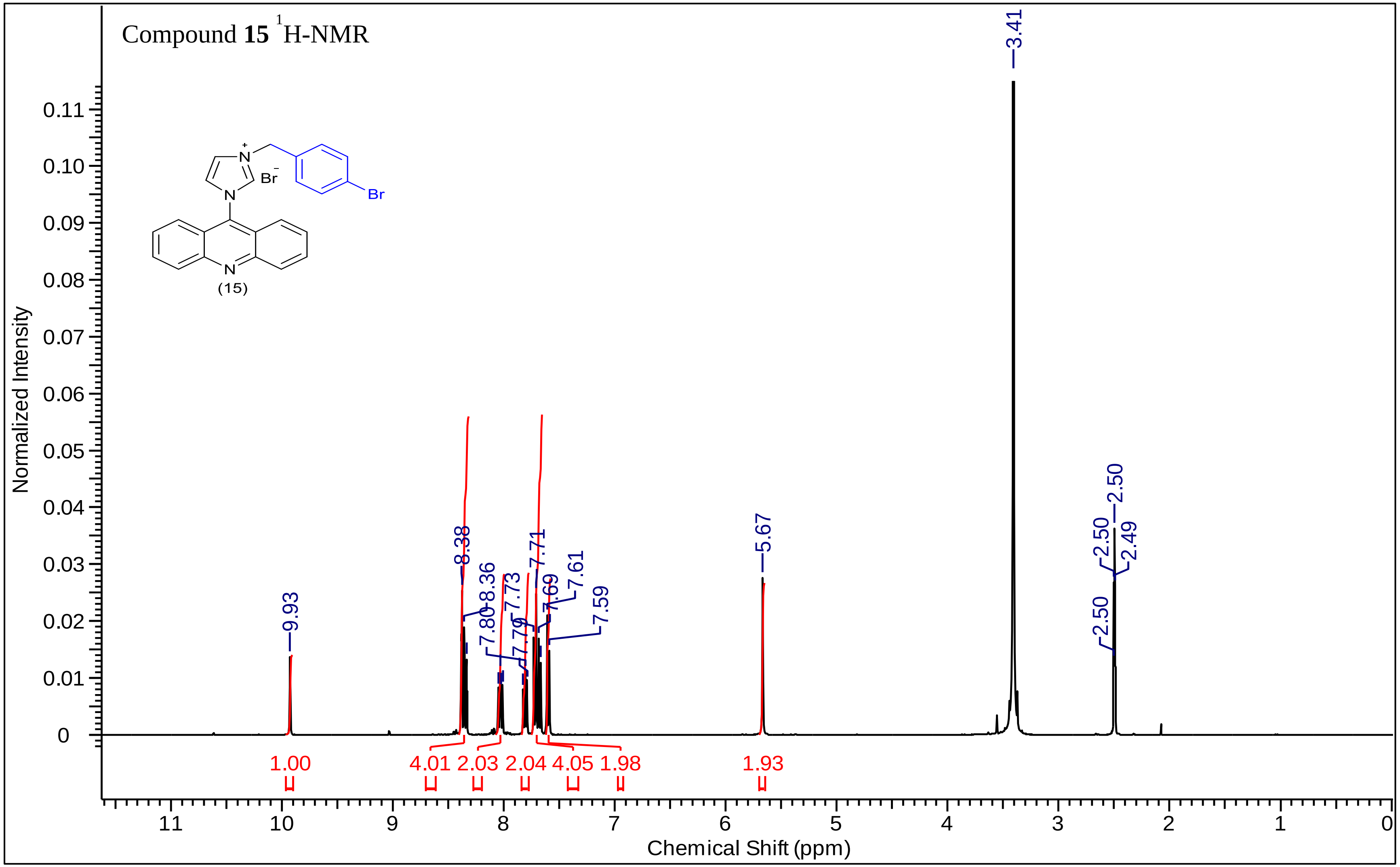
Compound 15 1H-NMR
N
N
Br
Br
(15)
Normalized Intensity
Chemical Shift (ppm)
0.11
0.10
0.09
0.08
0.07
0.06
0.05
0.04
0.03
0.02
0.01
0
11
10
9
8
7
6
5
4
3
2
1
0
9.93
8.38
8.36
7.80
7.79
7.73
7.71
7.69
7.61
7.59
5.67
3.41
2.50
2.50
2.50
2.49
1.00
4.01
2.03
2.04
4.05
1.98
1.93

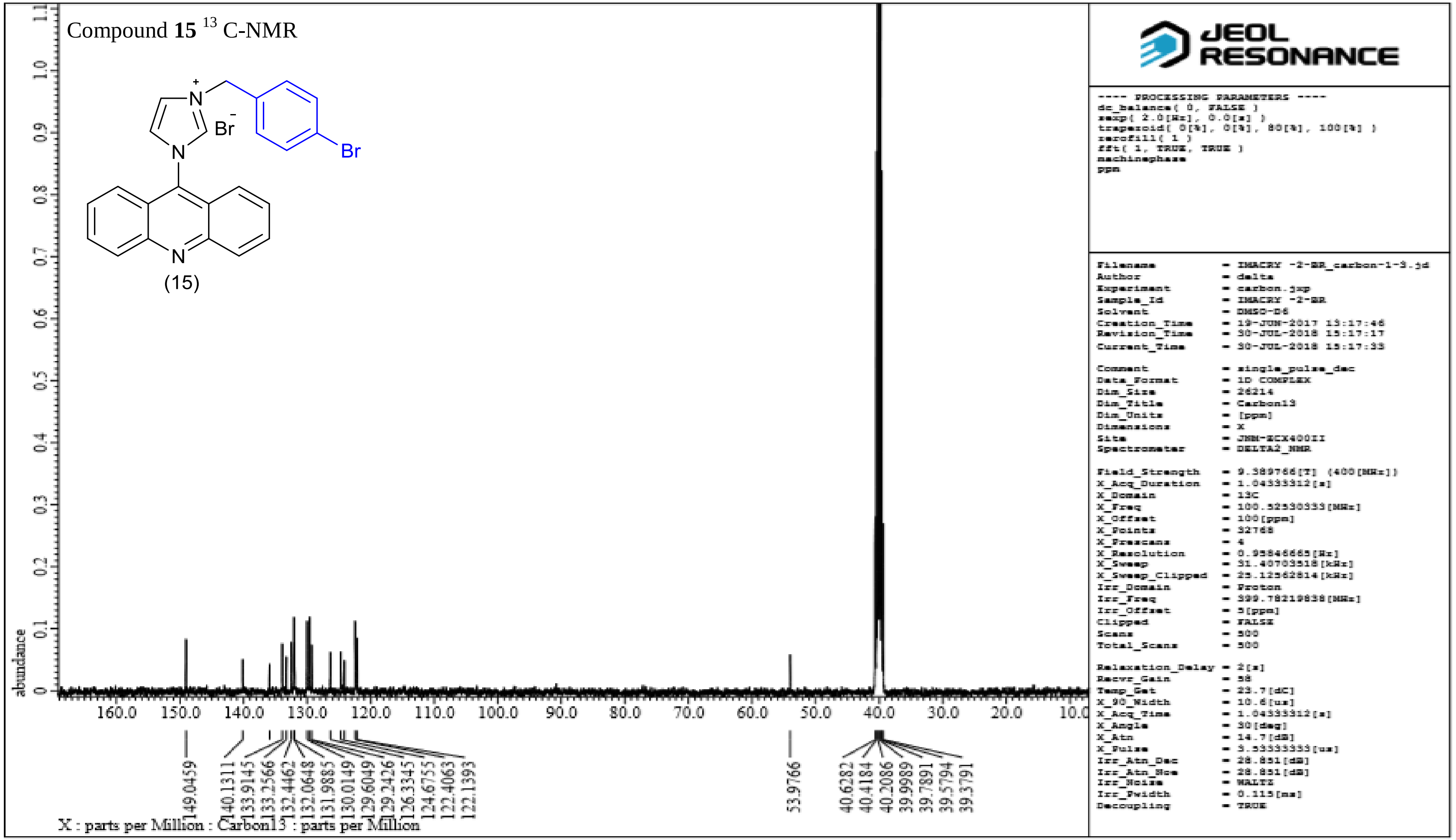
Compound 15 13 C-NMR
N+
Br-
Br
N
N
(15)
abundance
0
0.1
0.2
0.3
0.4
0.5
0.6
0.7
0.8
0.9
1.0
1.1
160.0
150.0
140.0
130.0
120.0
110.0
100.0
90.0
80.0
70.0
60.0
50.0
40.0
30.0
20.0
10.0
149.0459
140.1311
133.9145
133.2566
132.4462
132.0648
131.9885
130.0149
129.6049
129.2426
126.3345
124.6755
122.4063
122.1393
53.9766
40.6282
40.4184
40.2086
39.9989
39.7891
39.5794
39.3791
X : parts per Million : Carbon13 : parts per Million
JEOL RESONANCE
---- PROCESSING PARAMETERS ----
dc_balance(0, FALSE)
sexp(2.0[Hz], 0.0[s])
trapezoid(0[%], 0[%], 80[%], 100[%])
zerofill(1)
fft(1, TRUE, TRUE)
machinephase
ppm
Filename = IMACRY -2-BR_carbon-1-3.jd
Author = delta
Experiment = carbon.jxp
Sample_Id = IMACRY -2-BR
Solvent = DMSO-D6
Creation_Time = 19-JUN-2017 13:17:46
Revision_Time = 30-JUL-2018 13:17:17
Current_Time = 30-JUL-2018 13:17:33
Comment = single_pulse_dec
Data_Format = 1D COMPLEX
Dim_Size = 26214
Dim_Title = Carbon13
Dim_Units = [ppm]
Dimensions = X
Site = JNM-ECX400II
Spectrometer = DELTA2_NMR
Field_Strength = 9.389766[T] (400[MHz])
X_Acq_Duration = 1.04333312[s]
X_Domain = 13C
X_Freq = 100.52530333[MHz]
X_Offset = 100[ppm]
X_Points = 32768
X_Prescans = 4
X_Resolution = 0.95846665[Hz]
X_Sweep = 31.40703518[kHz]
X_Sweep_Clipped = 25.12562814[kHz]
Irr_Domain = Proton
Irr_Freq = 399.78219838[MHz]
Irr_Offset = 5[ppm]
Clipped = FALSE
Scans = 300
Total_Scans = 300
Relaxation_Delay = 2[s]
Recvr_Gain = 58
Temp_Get = 23.7[dC]
X_90_Width = 10.6[us]
X_Acq_Time = 1.04333312[s]
X_Angle = 30[deg]
X_Atn = 14.7[dB]
X_Pulse = 3.53333333[us]
Irr_Atn_Dec = 28.851[dB]
Irr_Atn_Noe = 28.851[dB]
Irr_Noise = WALTZ
Irr_Pwidth = 0.115[ms]
Decoupling = TRUE

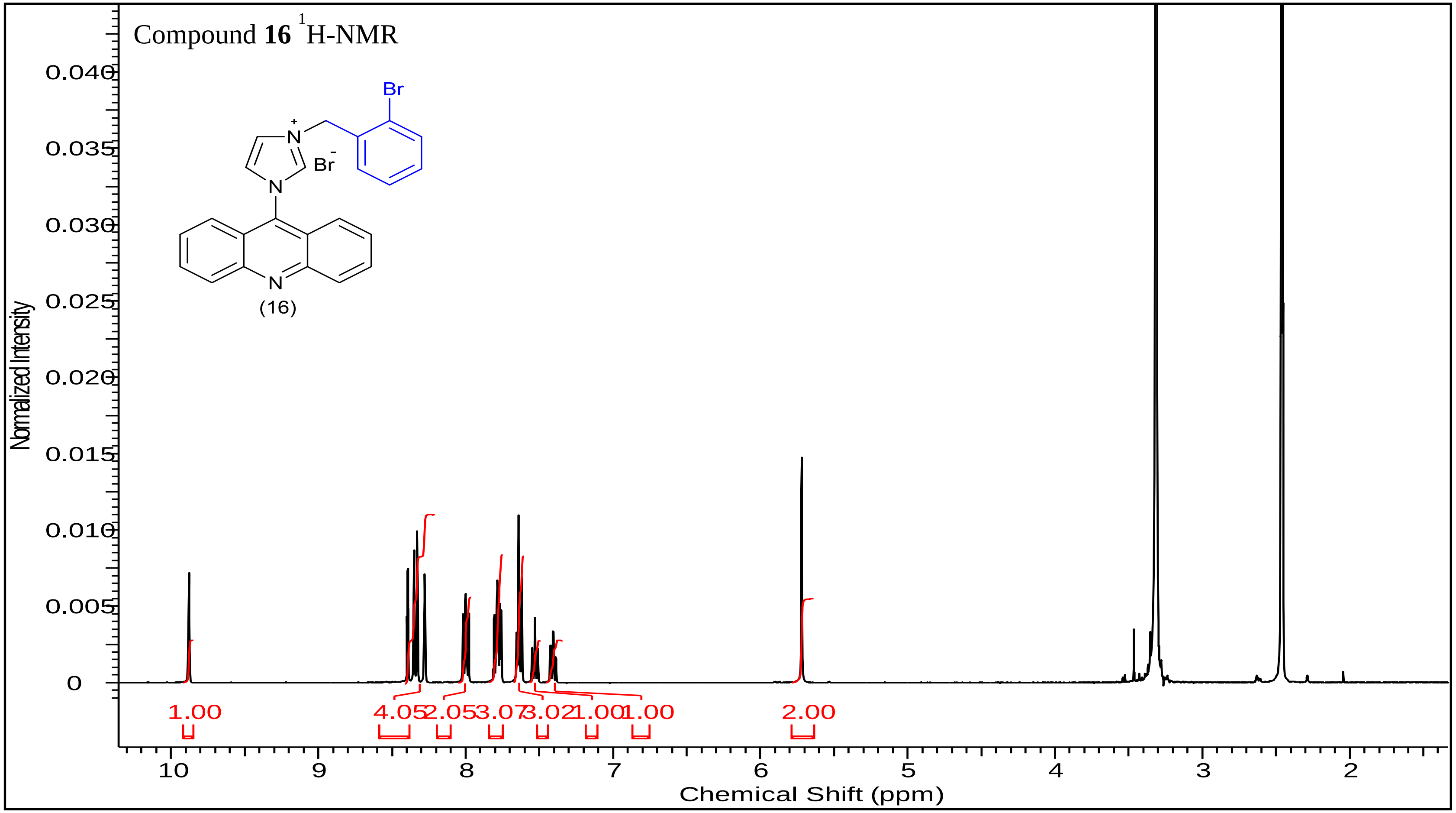
Compound 16 1H-NMR
Br
N
Br
N
N
(16)
0.040
0.035
0.030
0.025
0.020
0.015
0.010
0.005
0
Normalized Intensity
1.00
4.05
2.05
3.07
3.02
1.00
1.00
2.00
10
9
8
7
6
5
4
3
2
Chemical Shift (ppm)

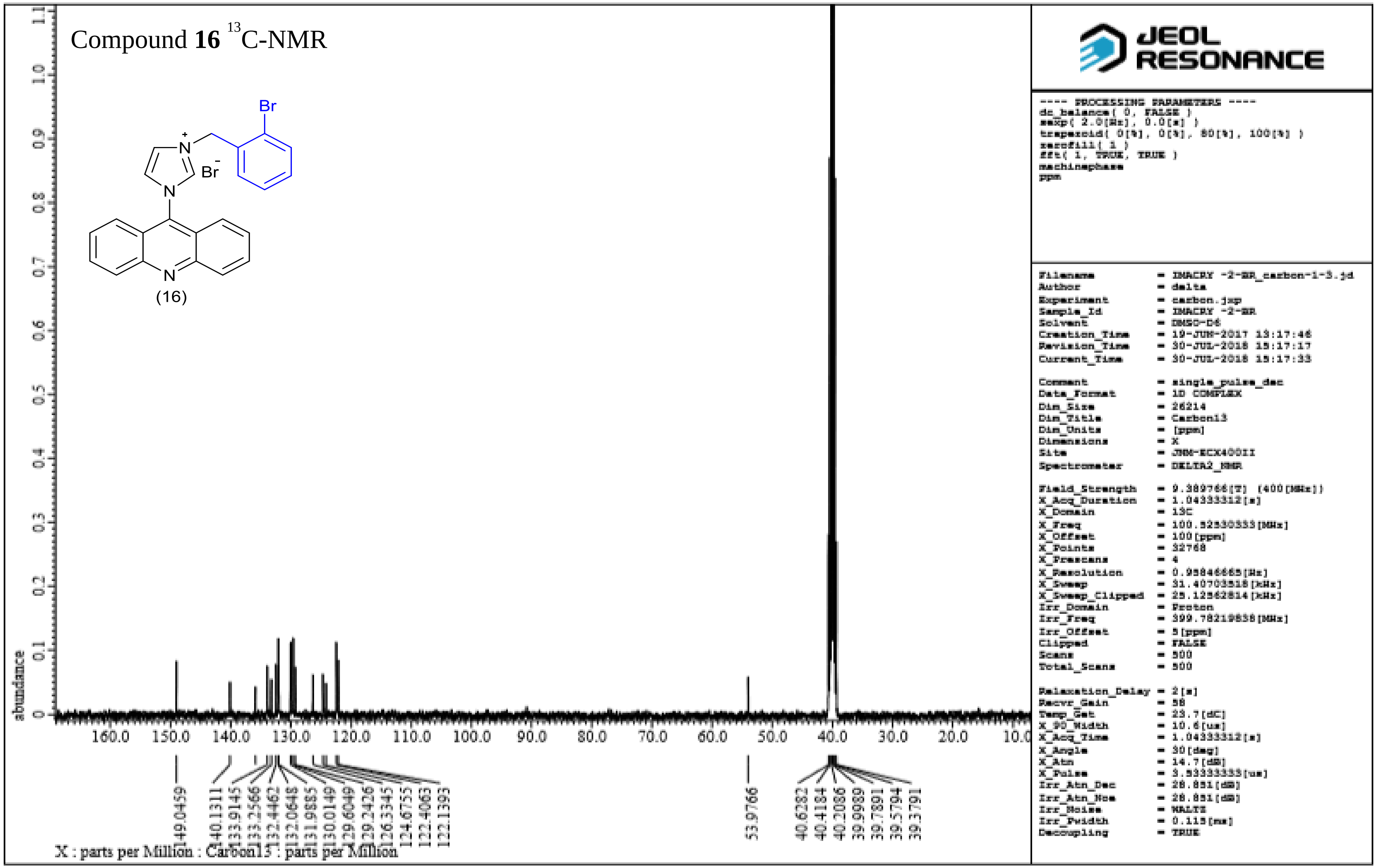
Compound 16 13C-NMR
Br
N+
Br-
N
N
(16)
abundance
X : parts per Million : Carbon13 : parts per Million
149.0459
140.1311
133.9145
133.2566
132.4462
132.0648
131.9885
130.0149
129.6049
129.2426
126.3345
124.6755
122.4063
122.1393
53.9766
40.6282
40.4184
40.2086
39.9989
39.7891
39.5794
39.3791
JEOL RESONANCE
---- PROCESSING PARAMETERS ----
dc_balance(0, FALSE)
sexp(2.0[Hz], 0.0[s])
trapezoid(0[%], 0[%], 80[%], 100[%])
zerofill(1)
fft(1, TRUE, TRUE)
machinephase
ppm
Filename = IMACRY -2-BR_carbon-1-3.jd
Author = delta
Experiment = carbon.jxp
Sample_Id = IMACRY -2-BR
Solvent = DMSO-D6
Creation_Time = 19-JUN-2017 13:17:46
Revision_Time = 30-JUL-2018 15:17:17
Current_Time = 30-JUL-2018 15:17:33
Comment = single_pulse_dec
Data_Format = 1D COMPLEX
Dim_Size = 26214
Dim_Title = Carbon13
Dim_Units = [ppm]
Dimensions = X
Site = JNM-ECX400II
Spectrometer = DELTA2_NMR
Field_Strength = 9.389766[T] (400[MHz])
X_Acq_Duration = 1.04333312[s]
X_Domain = 13C
X_Freq = 100.52530333[MHz]
X_Offset = 100[ppm]
X_Points = 32768
X_Prescans = 4
X_Resolution = 0.95846665[Hz]
X_Sweep = 31.40703518[kHz]
X_Sweep_Clipped = 25.12562814[kHz]
Irr_Domain = Proton
Irr_Freq = 399.78219838[MHz]
Irr_Offset = 5[ppm]
Clipped = FALSE
Scans = 500
Total_Scans = 500
Relaxation_Delay = 2[s]
Recvr_Gain = 58
Temp_Get = 23.7[dC]
X_90_Width = 10.6[us]
X_Acq_Time = 1.04333312[s]
X_Angle = 30[deg]
X_Atn = 14.7[dB]
X_Pulse = 3.53333333[us]
Irr_Atn_Dec = 28.851[dB]
Irr_Atn_Noe = 28.851[dB]
Irr_Noise = WALTZ
Irr_Pwidth = 0.115[ms]
Decoupling = TRUE

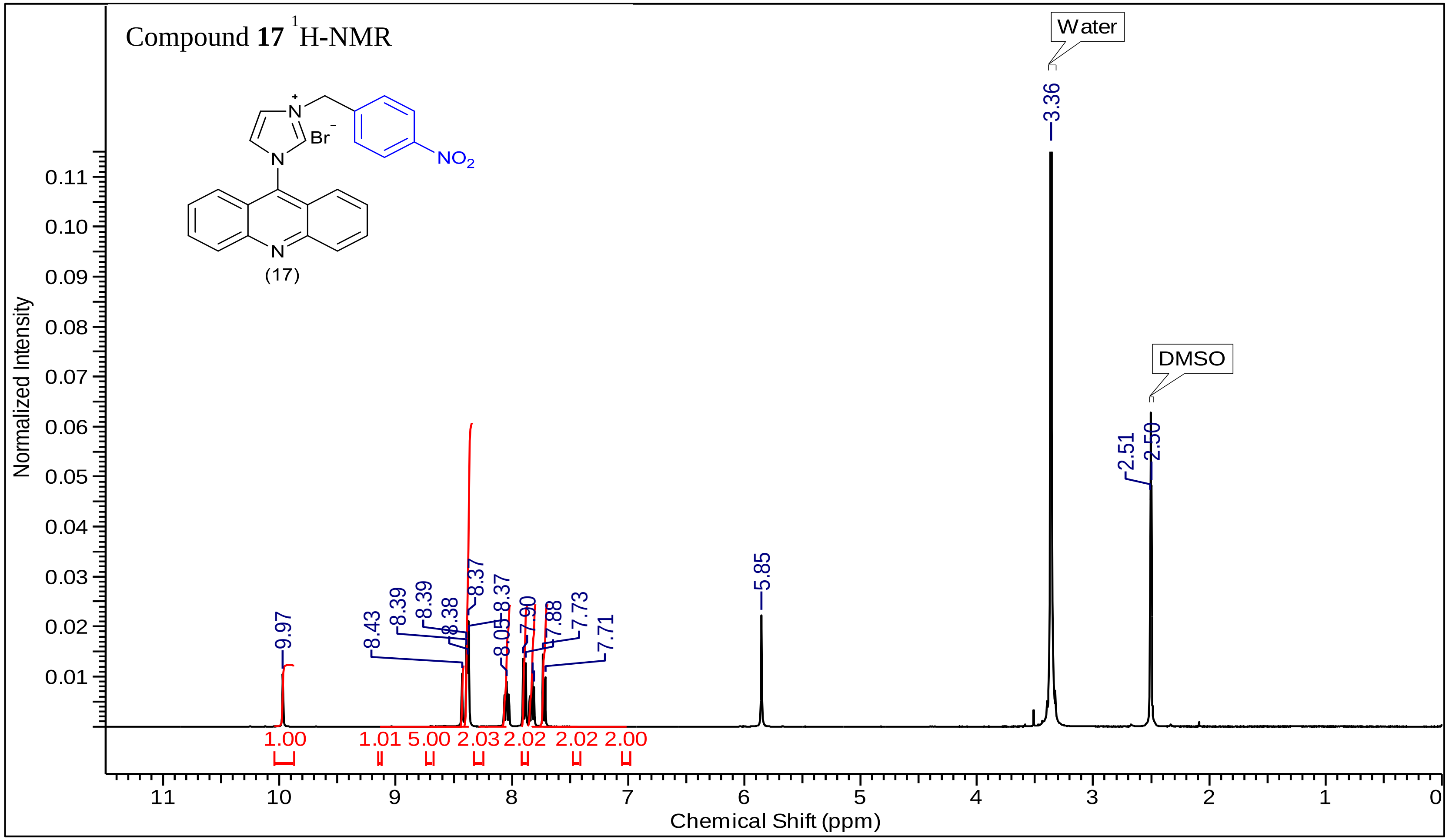

Compound **17** ^{1}H-NMR
Br⁻
NO_2
N
N
N
(17)
Water
DMSO
3.36
2.51
2.50
5.85
9.97
8.43
8.39
8.39
8.38
8.37
8.37
8.05
7.90
7.88
7.73
7.71
1.00
1.01
5.00
2.03
2.02
2.02
2.00
Normalized Intensity
Chemical Shift (ppm)

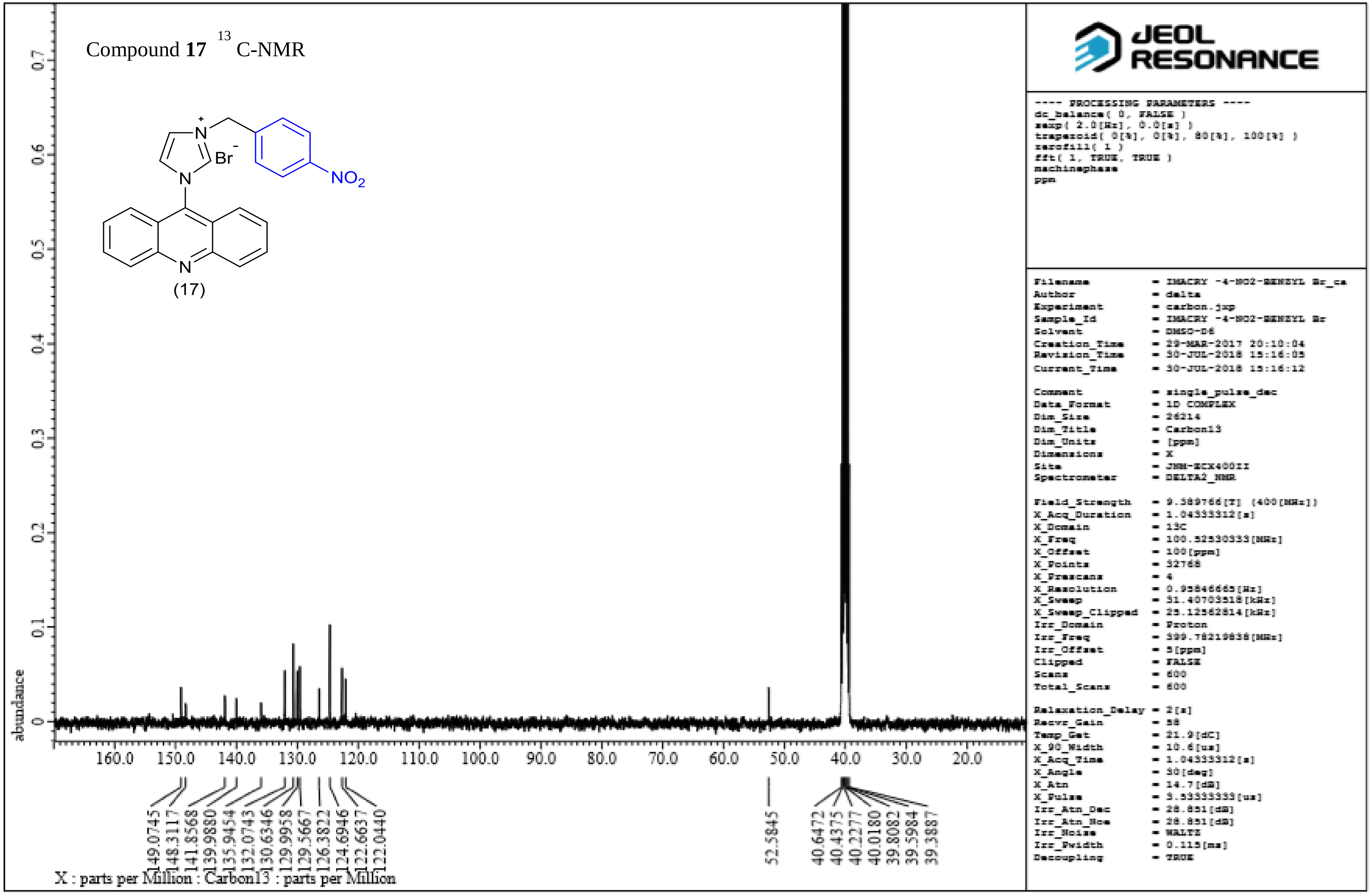

Compound 17 13C-NMR
Br-
NO2
(17)
JEOL RESONANCE
---- PROCESSING PARAMETERS ----
dc_balance(0, FALSE)
sexp(2.0[Hz], 0.0[s])
trapezoid(0[%], 0[%], 80[%], 100[%])
zerofill(1)
fft(1, TRUE, TRUE)
machinephase
ppm
Filename = IMACRY -4-NO2-BENZYL Br_ca
Author = delta
Experiment = carbon.jxp
Sample_Id = IMACRY -4-NO2-BENZYL Br
Solvent = DMSO-D6
Creation_Time = 29-MAR-2017 20:10:04
Revision_Time = 30-JUL-2018 15:16:05
Current_Time = 30-JUL-2018 15:16:12
Comment = single_pulse_dec
Data_Format = 1D COMPLEX
Dim_Size = 26214
Dim_Title = Carbon13
Dim_Units = [ppm]
Dimensions = X
Site = JNM-ECX400II
Spectrometer = DELTA2_NMR
Field_Strength = 9.389766[T] (400[MHz])
X_Acq_Duration = 1.04333312[s]
X_Domain = 13C
X_Freq = 100.52530333[MHz]
X_Offset = 100[ppm]
X_Points = 32768
X_Prescans = 4
X_Resolution = 0.95846665[Hz]
X_Sweep = 31.40703518[kHz]
X_Sweep_Clipped = 25.12562814[kHz]
Irr_Domain = Proton
Irr_Freq = 399.78219838[MHz]
Irr_Offset = 5[ppm]
Clipped = FALSE
Scans = 600
Total_Scans = 600
Relaxation_Delay = 2[s]
Recvr_Gain = 58
Temp_Get = 21.9[dC]
X_90_Width = 10.6[us]
X_Acq_Time = 1.04333312[s]
X_Angle = 30[deg]
X_Atn = 14.7[dB]
X_Pulse = 3.53333333[us]
Irr_Atn_Dec = 28.851[dB]
Irr_Atn_Noe = 28.851[dB]
Irr_Noise = WALTZ
Irr_Pwidth = 0.115[ms]
Decoupling = TRUE
abundance
0.7
0.6
0.5
0.4
0.3
0.2
0.1
0
160.0
150.0
140.0
130.0
120.0
110.0
100.0
90.0
80.0
70.0
60.0
50.0
40.0
30.0
20.0
149.0745
148.3117
141.8568
139.9880
135.9454
132.0743
130.6346
129.9958
129.5667
126.3822
124.6946
122.6637
122.0440
52.5845
40.6472
40.4375
40.2277
40.0180
39.8082
39.5984
39.3887
X : parts per Million : Carbon13 : parts per Million

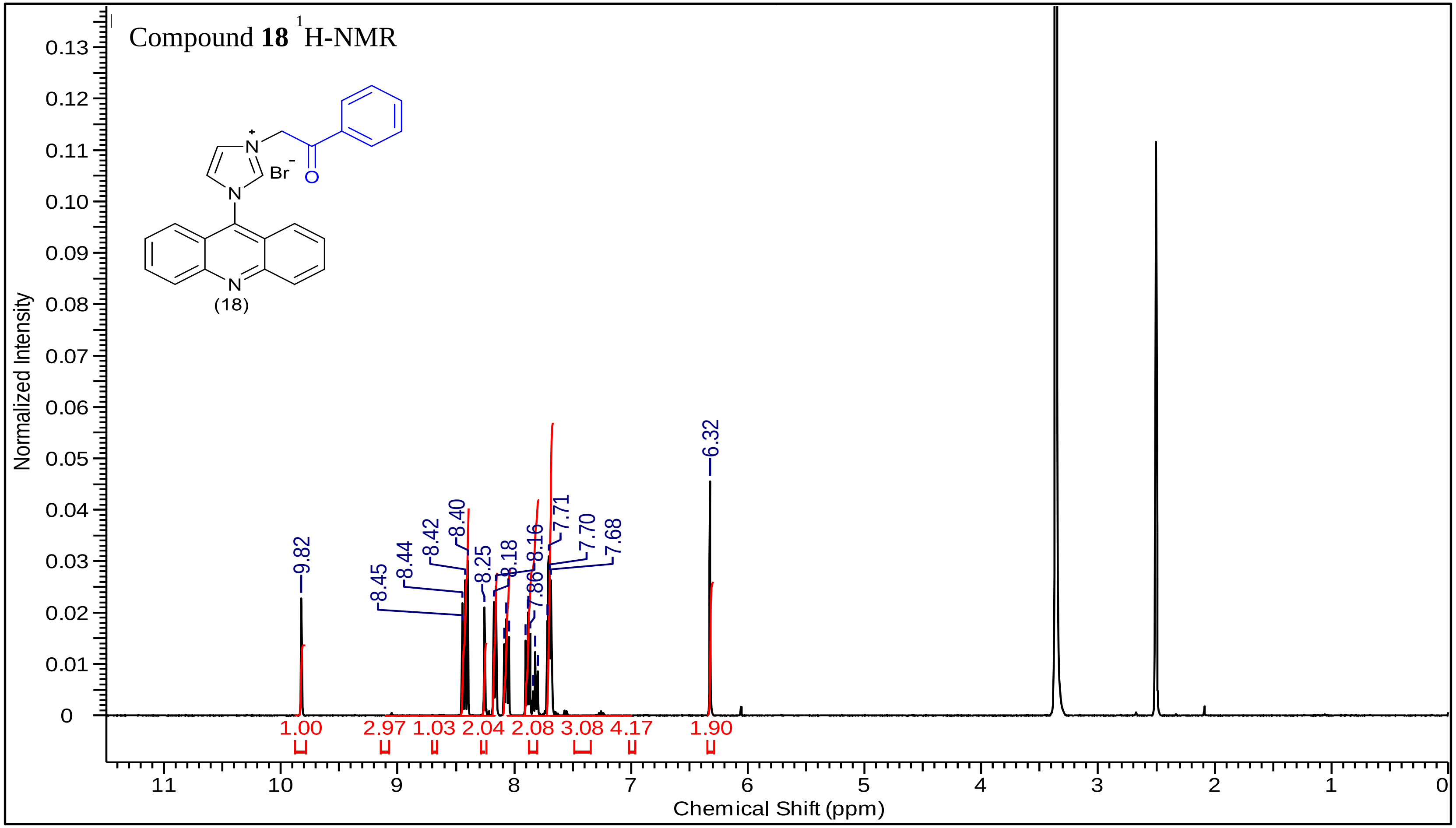
Compound 18 1H-NMR
Br-
N
O
(18)
9.82
8.45
8.44
8.42
8.40
8.25
8.18
8.16
7.86
7.71
7.70
7.68
6.32
1.00
2.97
1.03
2.04
2.08
3.08
4.17
1.90
Normalized Intensity
Chemical Shift (ppm)

Compound **18** ^{13}C-NMR

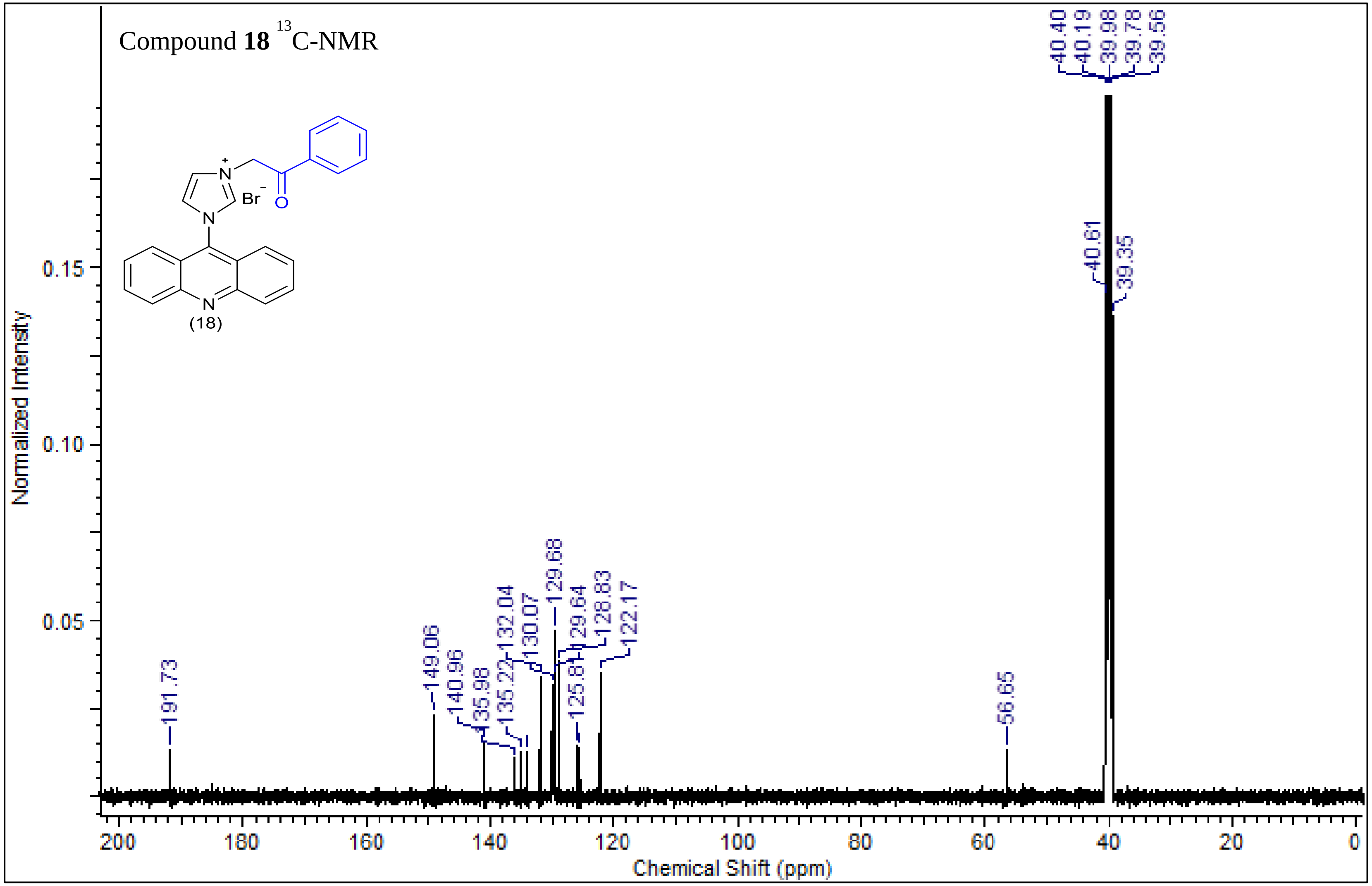

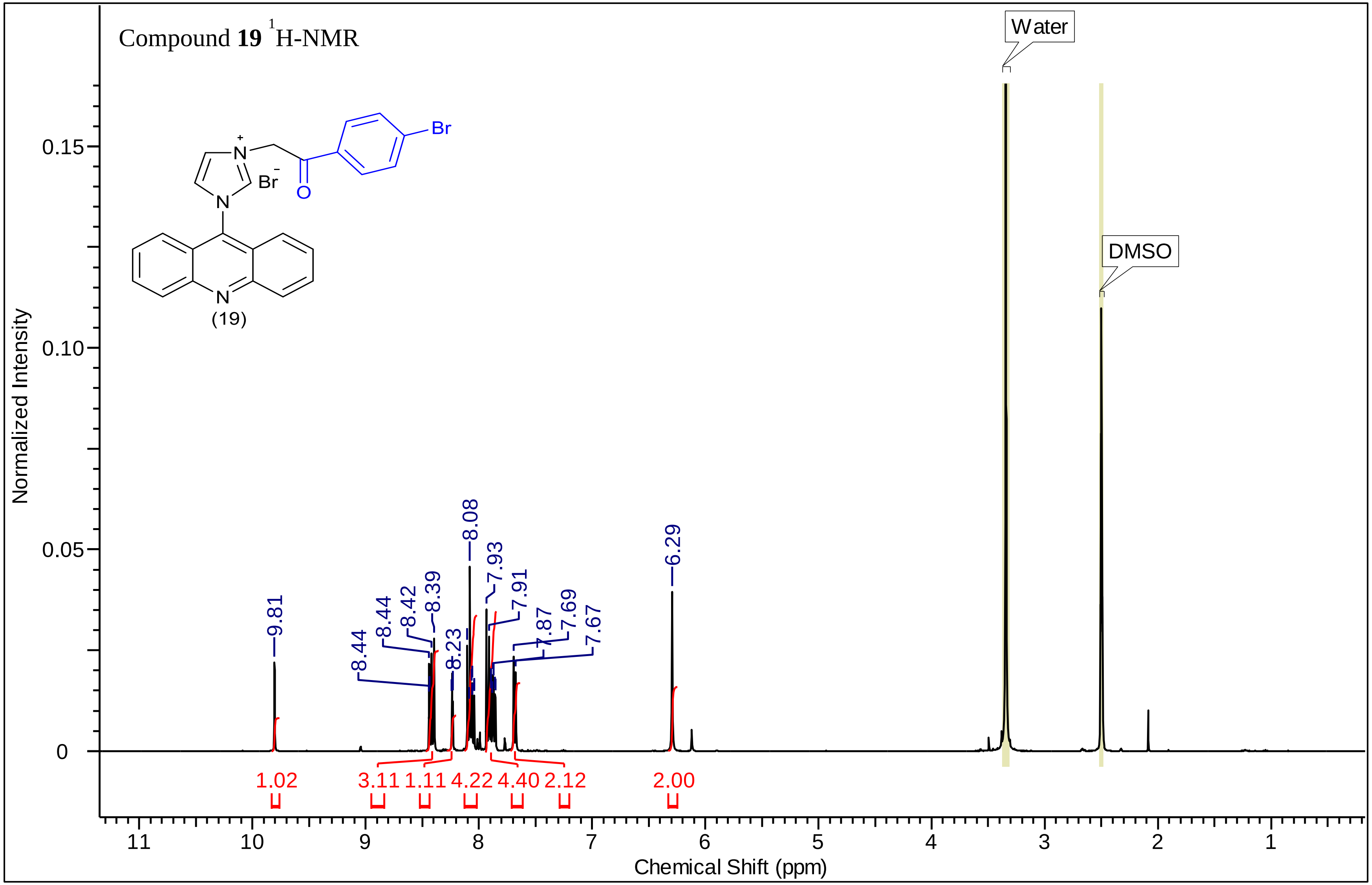
Compound 19 1H-NMR
Br
N
Br
N
O
N
(19)
Water
DMSO
9.81
8.44
8.44
8.42
8.39
8.23
8.08
7.93
7.91
7.87
7.69
7.67
6.29
1.02
3.11
1.11
4.22
4.40
2.12
2.00
Normalized Intensity
Chemical Shift (ppm)

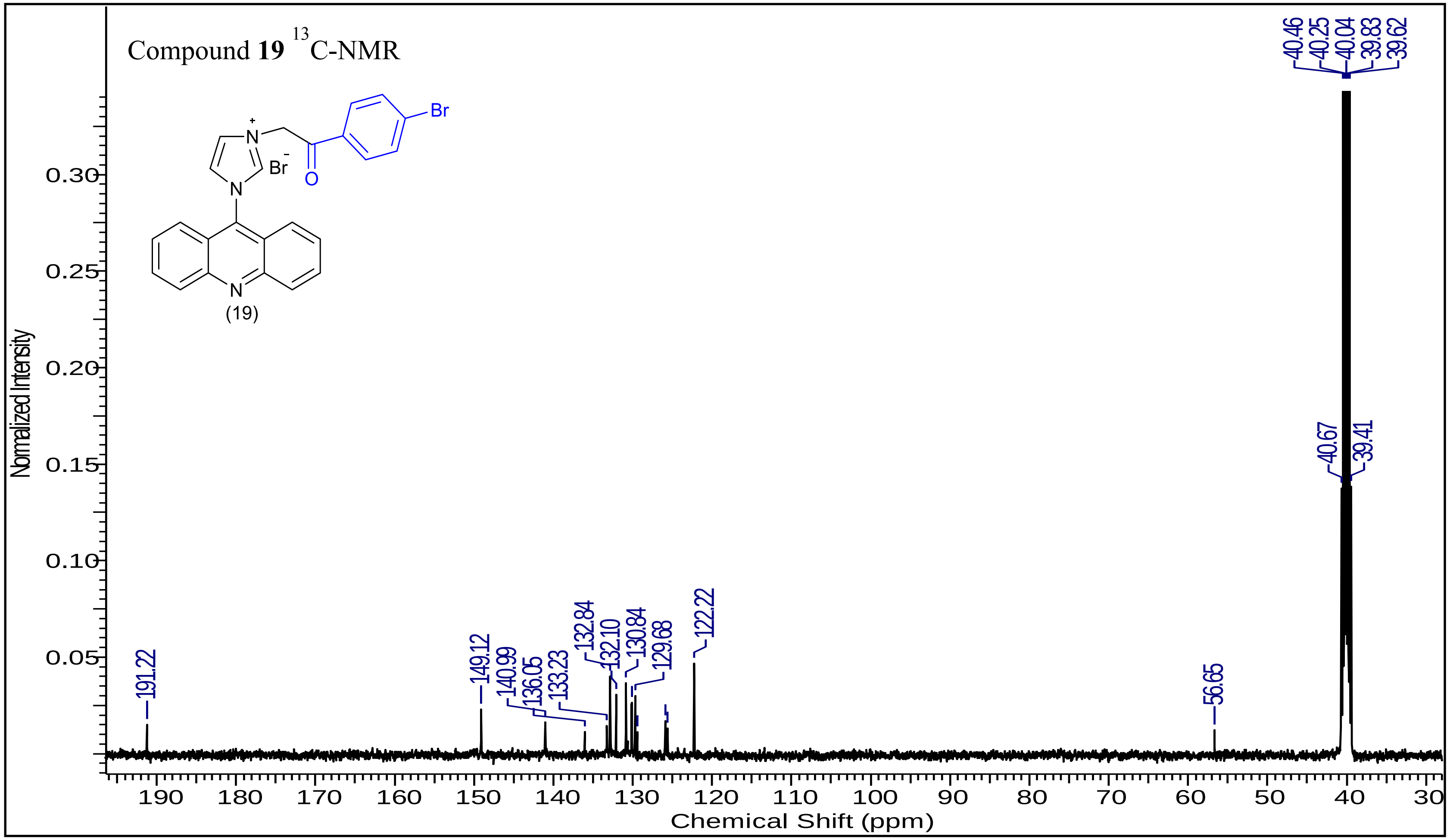

Compound 19 13C-NMR
Br
Br
O
N
N
N
(19)
40.46
40.25
40.04
39.83
39.62
40.67
39.41
191.22
149.12
140.99
136.05
133.23
132.84
132.10
130.84
129.68
122.22
56.65
0.30
0.25
0.20
0.15
0.10
0.05
Normalized Intensity
190 180 170 160 150 140 130 120 110 100 90 80 70 60 50 40 30
Chemical Shift (ppm)

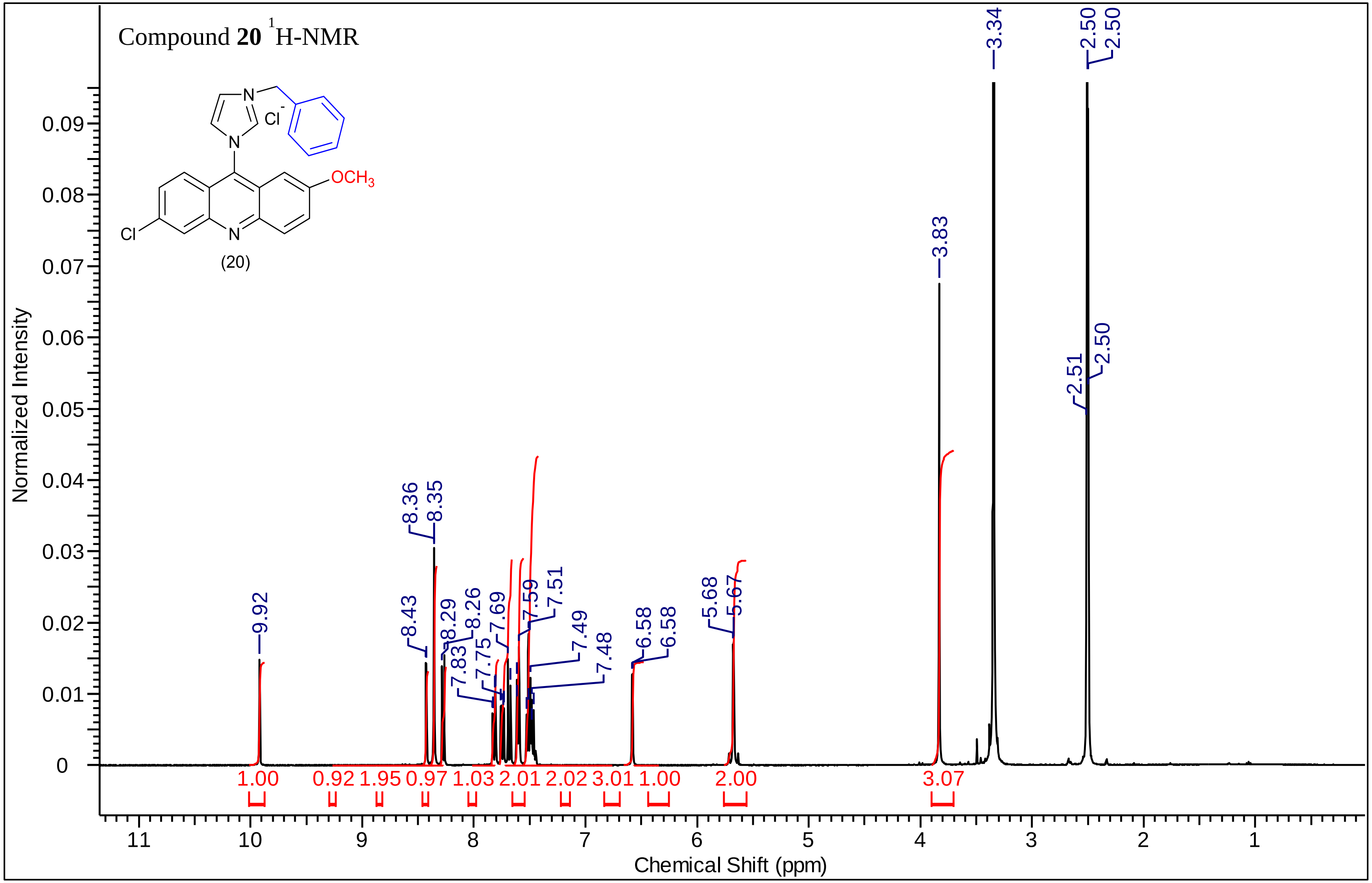

Compound 20 1H-NMR
N
N
Cl-
OCH3
Cl
N
(20)
Normalized Intensity
Chemical Shift (ppm)
0.09
0.08
0.07
0.06
0.05
0.04
0.03
0.02
0.01
0
11
10
9
8
7
6
5
4
3
2
1
9.92
8.43
8.36
8.35
8.29
8.26
7.83
7.75
7.69
7.59
7.51
7.49
7.48
6.58
6.58
5.68
5.67
3.83
3.34
2.51
2.50
2.50
2.50
1.00
0.92
1.95
0.97
1.03
2.01
2.02
3.01
1.00
2.00
3.07

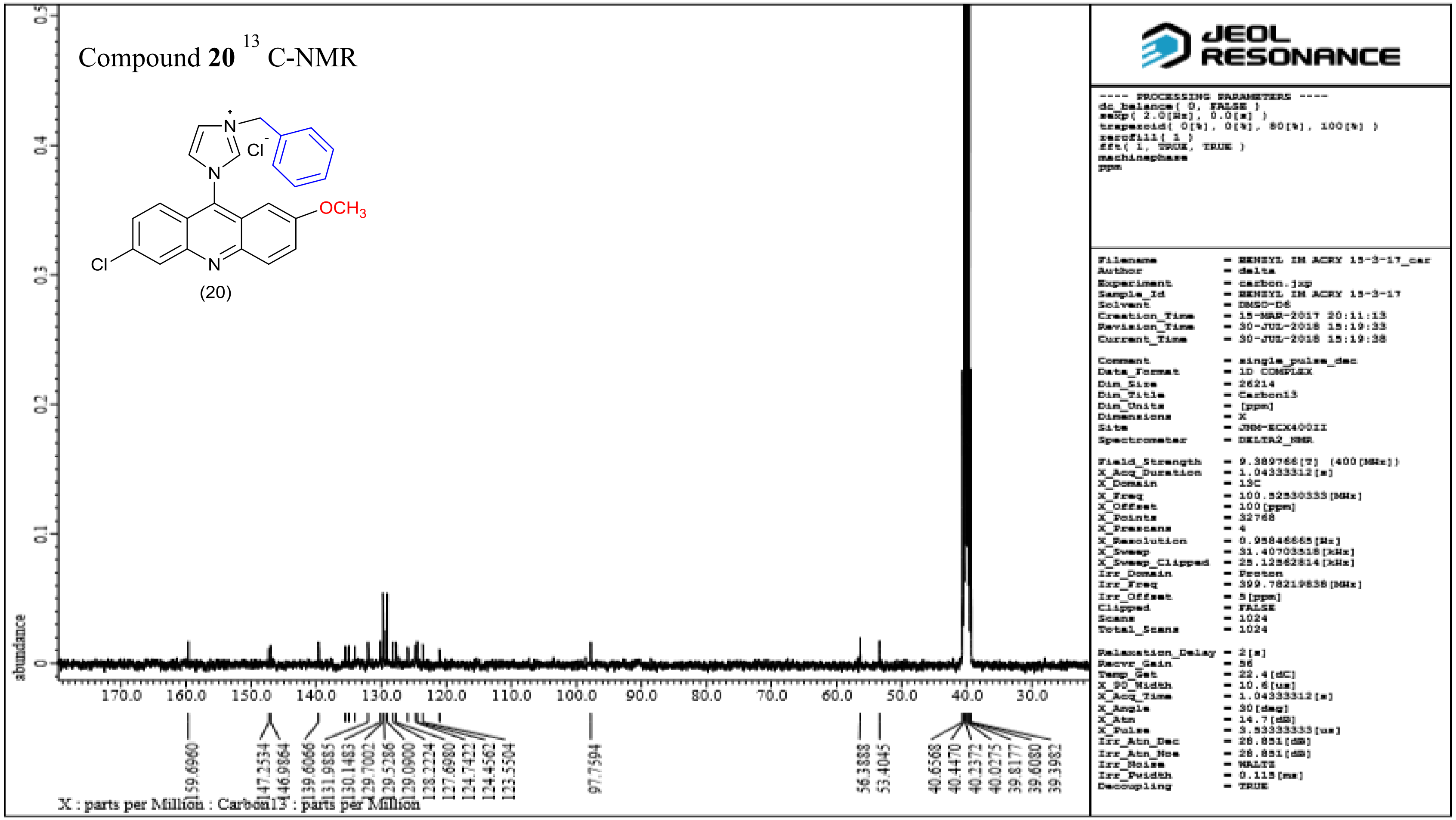
Compound 20 13 C-NMR
(20)
OCH3
Cl
N
Cl-
JEOL RESONANCE
abundance
X : parts per Million : Carbon13 : parts per Million
159.6960
147.2534
146.9864
139.6066
131.9885
130.1483
129.7002
129.5286
129.0900
128.2224
127.6980
124.7422
124.4562
123.5504
97.7594
56.3888
53.4045
40.6568
40.4470
40.2372
40.0275
39.8177
39.6080
39.3982

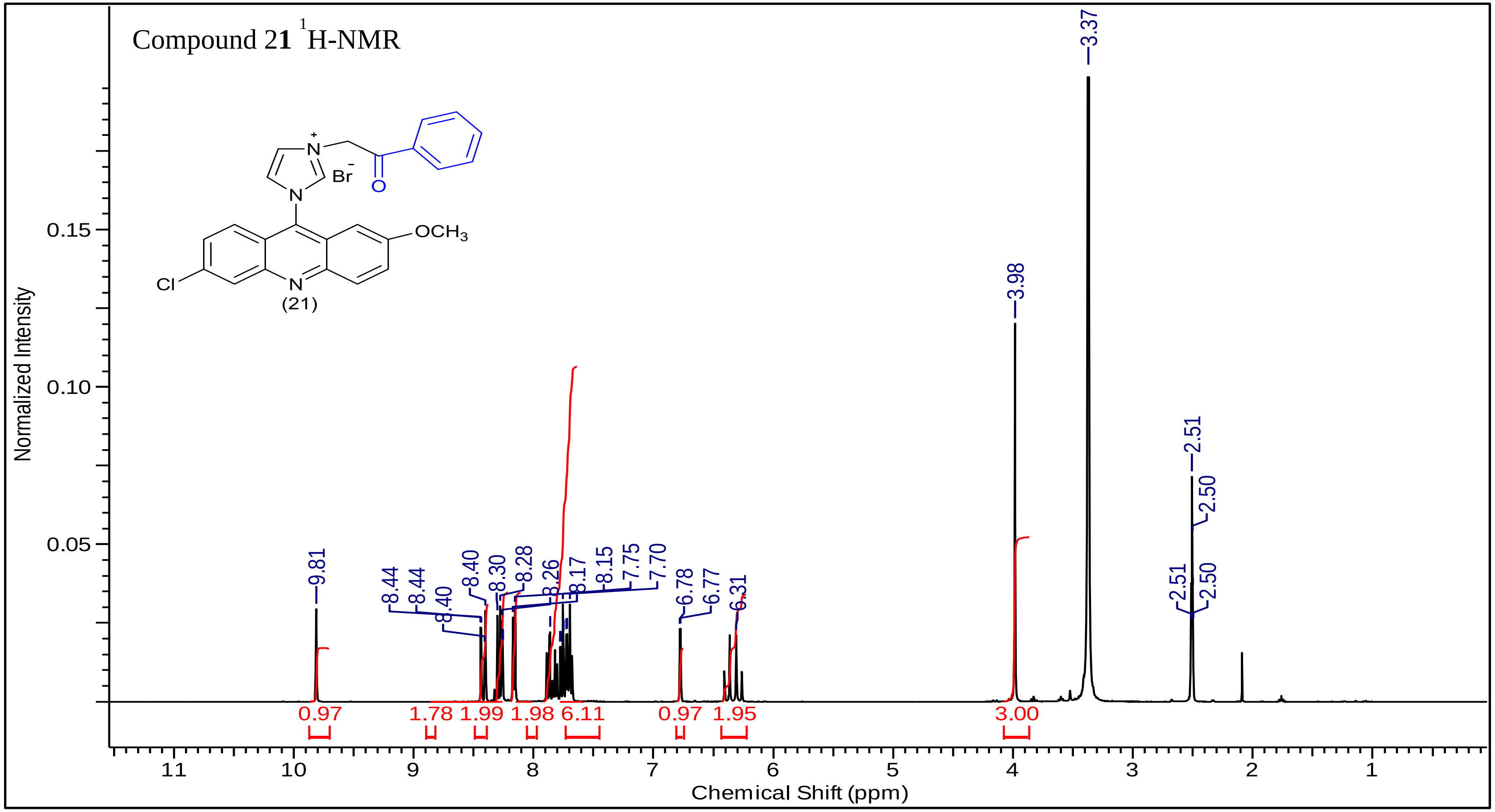
Compound 21 1H-NMR
Normalized Intensity
0.15
0.10
0.05
Chemical Shift (ppm)
11 10 9 8 7 6 5 4 3 2 1
9.81
8.44
8.44
8.40
8.40
8.30
8.28
8.26
8.17
8.15
7.75
7.70
6.78
6.77
6.31
3.98
3.37
2.51
2.50
2.51
2.50
0.97
1.78
1.99
1.98
6.11
0.97
1.95
3.00
Br
OCH3
Cl
N
(21)

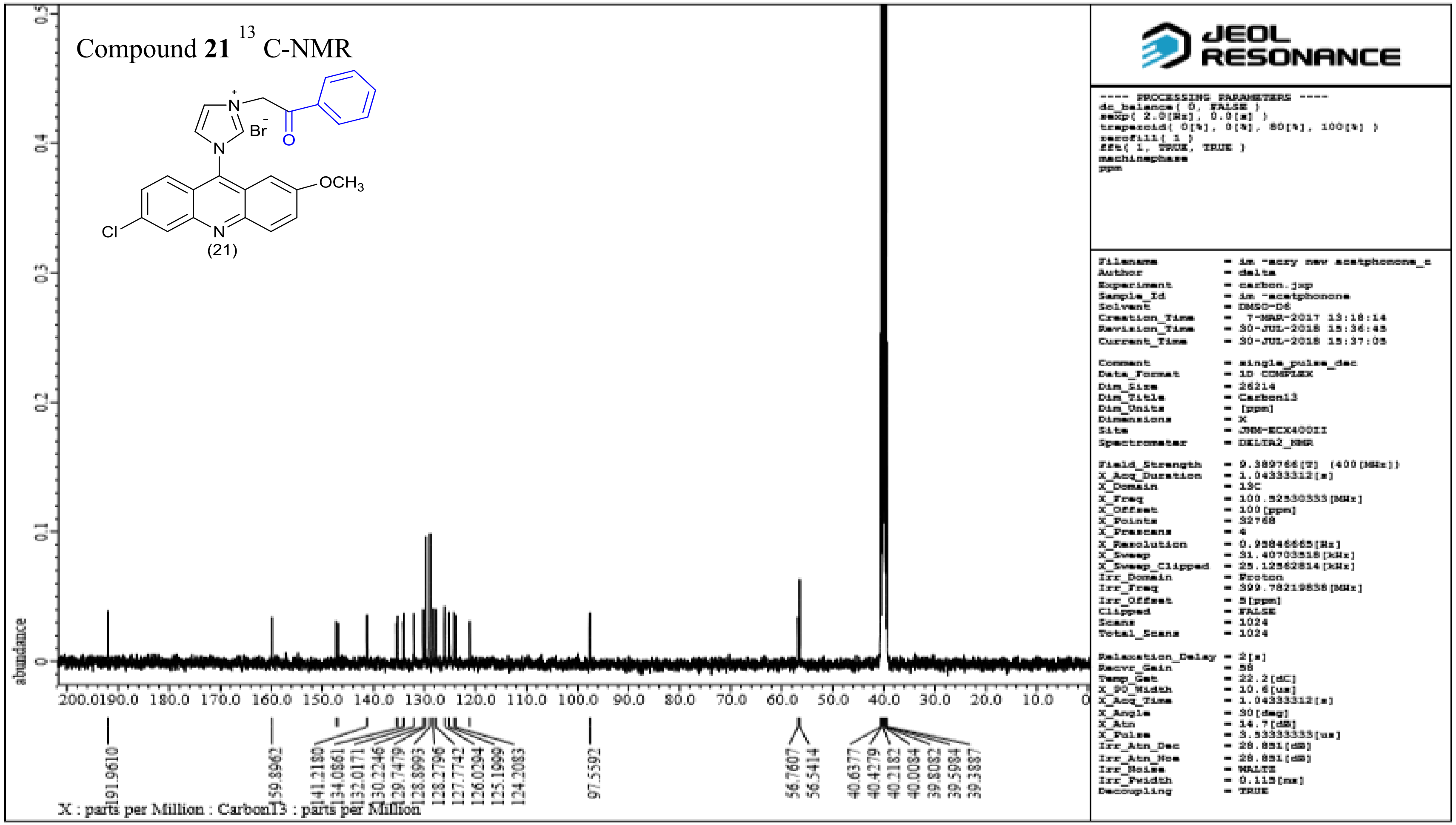

Compound 21 13C-NMR
Br⁻
O
OCH3
Cl
N
(21)
JEOL RESONANCE
---- PROCESSING PARAMETERS ----
dc_balance(0, FALSE)
sexp(2.0[Hz], 0.0[s])
trapezoid(0[%], 0[%], 80[%], 100[%])
zerofill(1)
fft(1, TRUE, TRUE)
machinephase
ppm
Filename = im -acry new acetphonone_c
Author = delta
Experiment = carbon.jxp
Sample_Id = im -acetphonone
Solvent = DMSO-D6
Creation_Time = 7-MAR-2017 13:18:14
Revision_Time = 30-JUL-2018 15:36:45
Current_Time = 30-JUL-2018 15:37:05
Comment = single_pulse_dec
Data_Format = 1D COMPLEX
Dim_Size = 26214
Dim_Title = Carbon13
Dim_Units = [ppm]
Dimensions = X
Site = JNM-ECX400II
Spectrometer = DELTA2_NMR
Field_Strength = 9.389766[T] (400[MHz])
X_Acq_Duration = 1.04333312[s]
X_Domain = 13C
X_Freq = 100.52530333[MHz]
X_Offset = 100[ppm]
X_Points = 32768
X_Prescans = 4
X_Resolution = 0.95846665[Hz]
X_Sweep = 31.40703518[kHz]
X_Sweep_Clipped = 25.12562814[kHz]
Irr_Domain = Proton
Irr_Freq = 399.78219838[MHz]
Irr_Offset = 5[ppm]
Clipped = FALSE
Scans = 1024
Total_Scans = 1024
Relaxation_Delay = 2[s]
Recvr_Gain = 58
Temp_Get = 22.2[dC]
X_90_Width = 10.6[us]
X_Acq_Time = 1.04333312[s]
X_Angle = 30[deg]
X_Atn = 14.7[dB]
X_Pulse = 3.53333333[us]
Irr_Atn_Dec = 28.851[dB]
Irr_Atn_Noe = 28.851[dB]
Irr_Noise = WALTZ
Irr_Pwidth = 0.115[ms]
Decoupling = TRUE
abundance
0.5
0.4
0.3
0.2
0.1
0
200.0 190.0 180.0 170.0 160.0 150.0 140.0 130.0 120.0 110.0 100.0 90.0 80.0 70.0 60.0 50.0 40.0 30.0 20.0 10.0 0
191.9610
159.8962
141.2180
134.0861
132.0171
130.2246
129.7479
128.8993
128.2796
127.7742
126.0294
125.1999
124.2083
97.5592
56.7607
56.5414
40.6377
40.4279
40.2182
40.0084
39.8082
39.5984
39.3887
X : parts per Million : Carbon13 : parts per Million

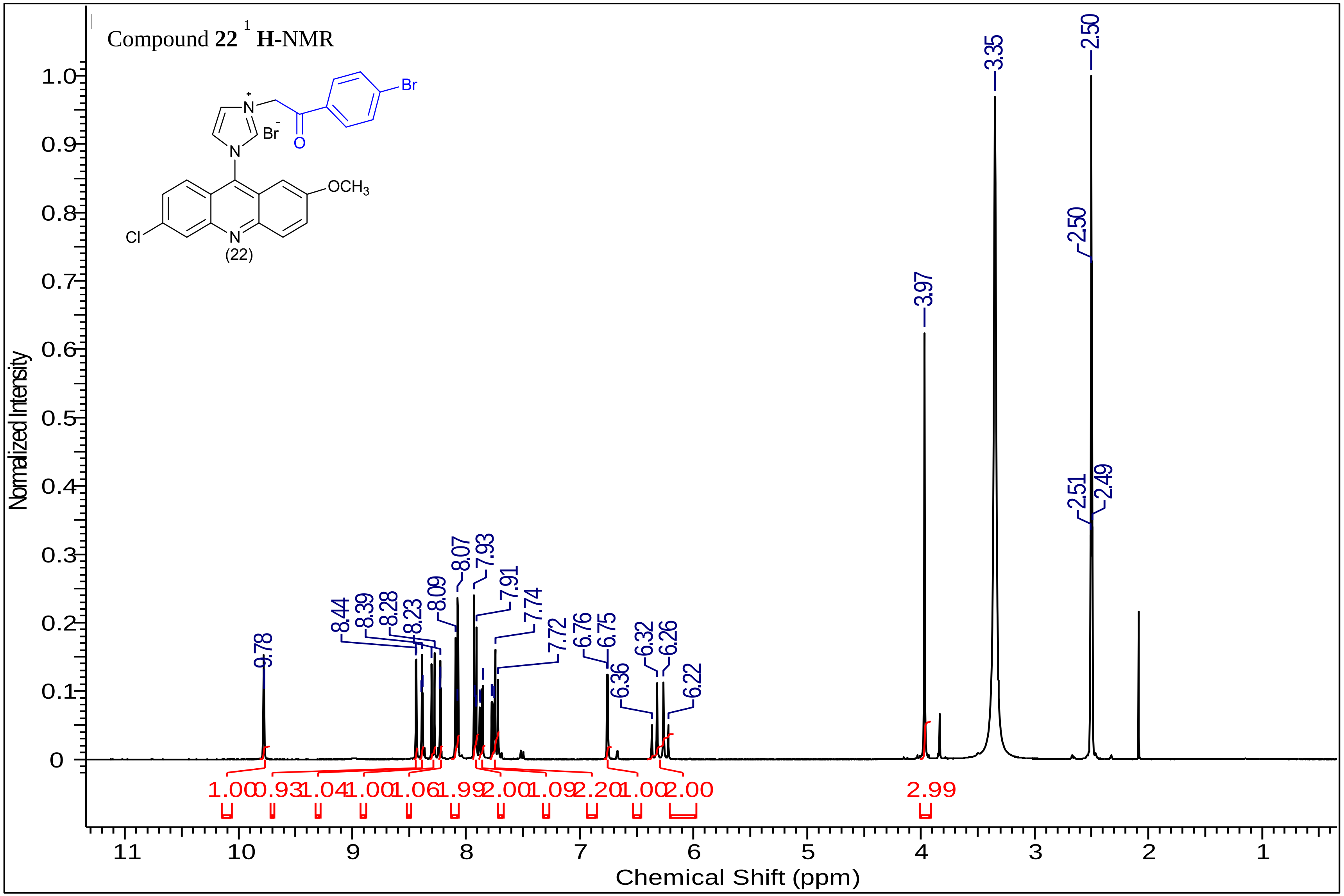
Compound 22 ¹H-NMR
Br
Br-
O
OCH3
Cl
N
(22)
Normalized Intensity
Chemical Shift (ppm)
9.78
8.44
8.39
8.28
8.23
8.09
8.07
7.93
7.91
7.74
7.72
6.76
6.75
6.36
6.32
6.26
6.22
3.97
3.35
2.50
2.50
2.51
2.49
1.00
0.93
1.04
1.00
1.06
1.99
2.00
1.09
2.20
1.00
2.00
2.99

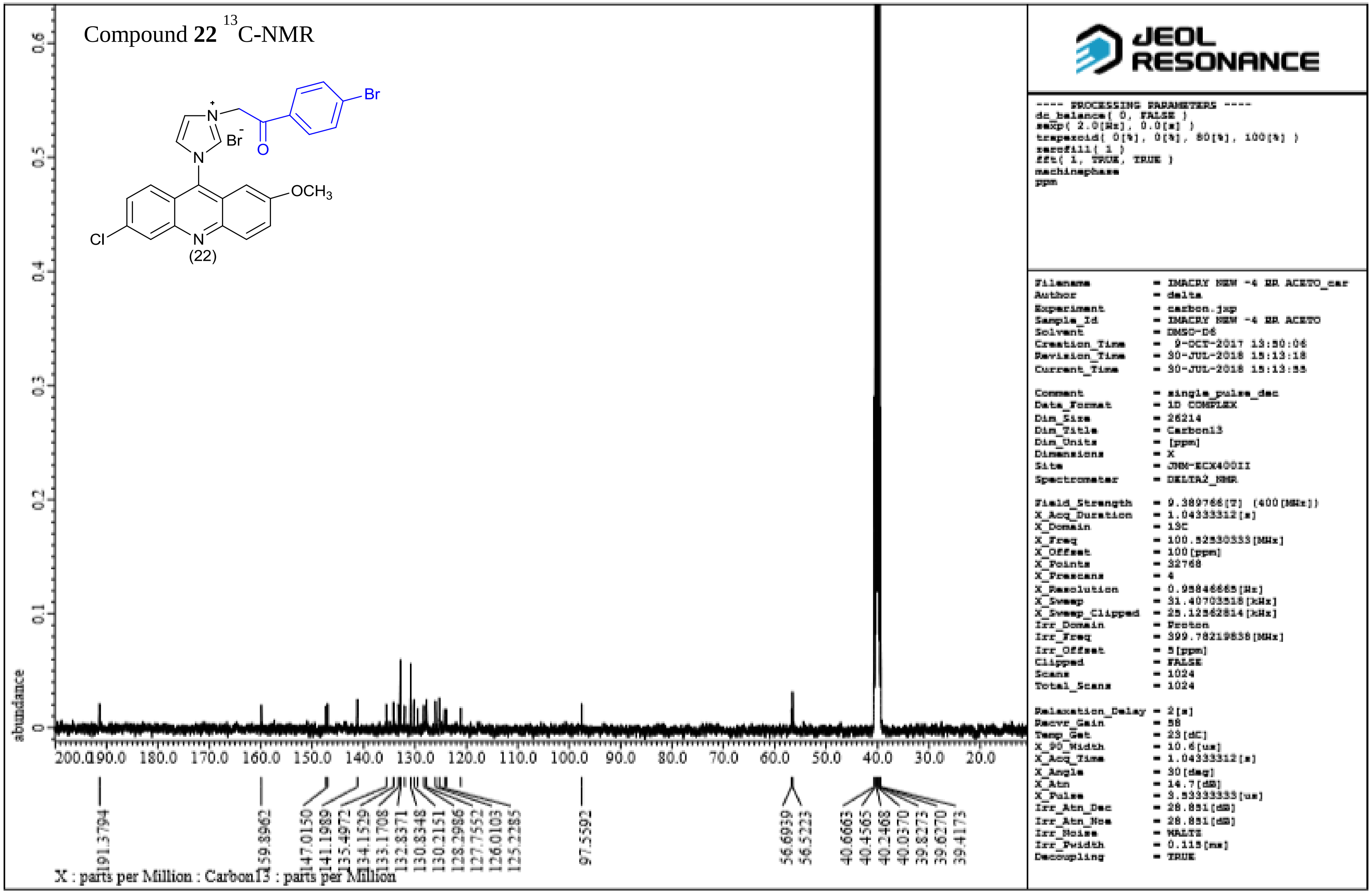
Compound 22 13C-NMR
Br
Br-
O
N+
N
OCH3
Cl
N
(22)
JEOL RESONANCE
---- PROCESSING PARAMETERS ----
dc_balance(0, FALSE)
sexp(2.0[Hz], 0.0[s])
trapezoid(0[%], 0[%], 80[%], 100[%])
zerofill(1)
fft(1, TRUE, TRUE)
machinephase
ppm
Filename = IMACRY NEW -4 BR ACETO_car
Author = delta
Experiment = carbon.jxp
Sample_Id = IMACRY NEW -4 BR ACETO
Solvent = DMSO-D6
Creation_Time = 9-OCT-2017 13:50:06
Revision_Time = 30-JUL-2018 15:13:18
Current_Time = 30-JUL-2018 15:13:55
Comment = single_pulse_dec
Data_Format = 1D COMPLEX
Dim_Size = 26214
Dim_Title = Carbon13
Dim_Units = [ppm]
Dimensions = X
Site = JNM-ECX400II
Spectrometer = DELTA2_NMR
Field_Strength = 9.389766[T] (400[MHz])
X_Acq_Duration = 1.04333312[s]
X_Domain = 13C
X_Freq = 100.52530333[MHz]
X_Offset = 100[ppm]
X_Points = 32768
X_Prescans = 4
X_Resolution = 0.95846665[Hz]
X_Sweep = 31.40703518[kHz]
X_Sweep_Clipped = 25.12562814[kHz]
Irr_Domain = Proton
Irr_Freq = 399.78219838[MHz]
Irr_Offset = 5[ppm]
Clipped = FALSE
Scans = 1024
Total_Scans = 1024
Relaxation_Delay = 2[s]
Recvr_Gain = 58
Temp_Get = 23[dC]
X_90_Width = 10.6[us]
X_Acq_Time = 1.04333312[s]
X_Angle = 30[deg]
X_Atn = 14.7[dB]
X_Pulse = 3.53333333[us]
Irr_Atn_Dec = 28.851[dB]
Irr_Atn_Noe = 28.851[dB]
Irr_Noise = WALTZ
Irr_Pwidth = 0.115[ms]
Decoupling = TRUE
abundance
0.6
0.5
0.4
0.3
0.2
0.1
0
200.0
190.0
180.0
170.0
160.0
150.0
140.0
130.0
120.0
110.0
100.0
90.0
80.0
70.0
60.0
50.0
40.0
30.0
20.0
191.3794
159.8962
147.0150
141.1989
135.4972
134.1529
133.1708
132.8371
130.8348
130.2151
128.2986
127.7552
126.0103
125.2285
97.5592
56.6939
56.5223
40.6663
40.4565
40.2468
40.0370
39.8273
39.6270
39.4173
X : parts per Million : Carbon13 : parts per Million

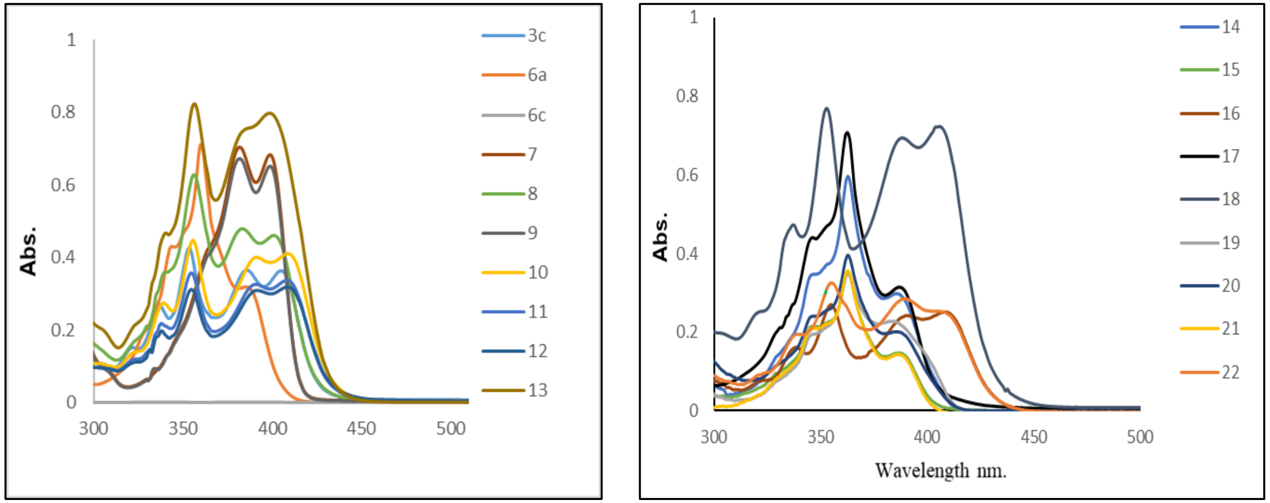

Figure. S 51. UV-Vis spectrum of compounds
